# Reduction of arenediazonium salts by tetrakis(dimethylamino)ethylene (TDAE): Efficient formation of products derived from aryl radicals

**DOI:** 10.3762/bjoc.5.1

**Published:** 2009-01-12

**Authors:** Mohan Mahesh, John A Murphy, Franck LeStrat, Hans Peter Wessel

**Affiliations:** 1 WestCHEM, Department of Pure and Applied Chemistry, University of Strathclyde, 295, Cathedral Street, Glasgow G1 1XL, U.K.; 2 Pharma Research, Discovery Chemistry, F. Hoffmann-La Roche Ltd., Basel, CH-4070, Switzerland

**Keywords:** cyclization, electron transfer, indole, indoline, radical

## Abstract

Tetrakis(dimethylamino)ethylene (TDAE **1**), has been exploited for the first time as a mild reagent for the reduction of arenediazonium salts to aryl radical intermediates through a single electron transfer (SET) pathway. Cyclization of the aryl radicals produced in this way led, in appropriate substrates, to syntheses of indolines and indoles. Cascade radical cyclizations of aryl radicals derived from arenediazonium salts are also reported. The relative ease of removal of the oxidized by-products of TDAE from the reaction mixture makes the methodology synthetically attractive.

## Introduction

**Scheme 1:**
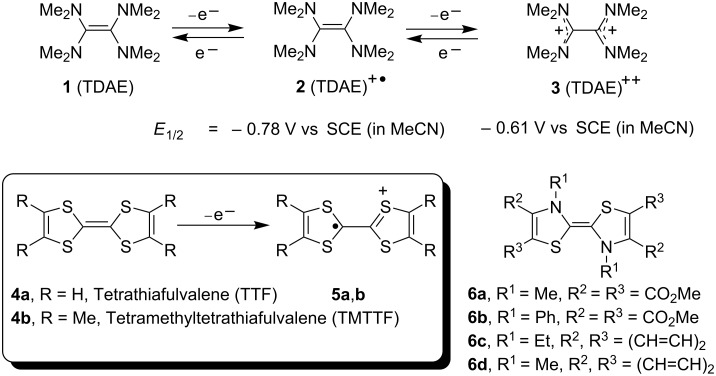
Aza- and thia-substituted electron donors.

**Scheme 2:**
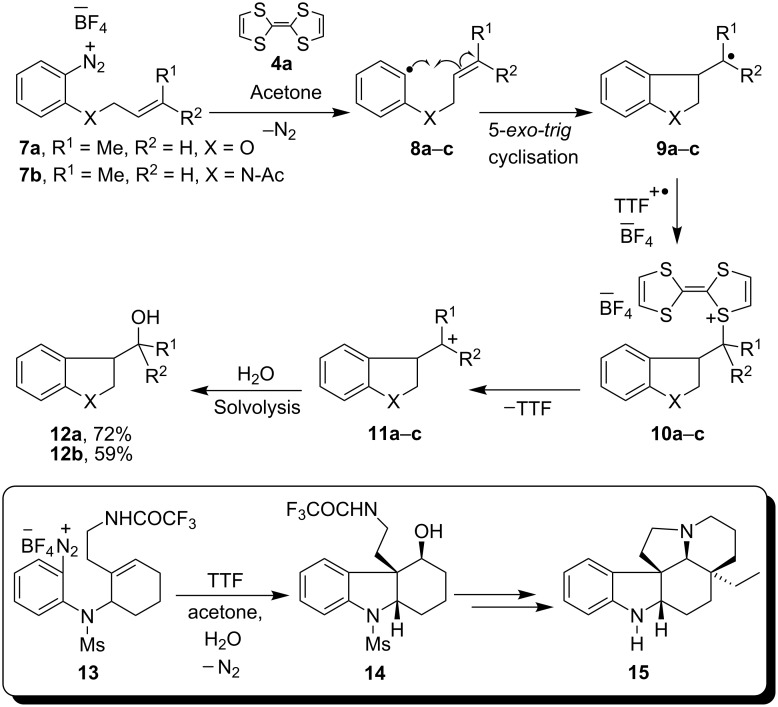
Radical-polar crossover reaction of arenediazonium salts by TTF.

Arenediazonium salts have long proved useful as sources of aryl radicals in many reactions featuring carbon-carbon (e.g., Meerwein [1], Pschorr [2–3], Gomberg [3] reactions) and carbon-heteroatom bond (e.g., Sandmeyer [4]) formation. The radical-polar crossover reaction [5–15] of arenediazonium salts, developed in our group since 1993, also features aryl radical intermediates and is a more recent addition to these reactions. It involves a novel splicing of radical and polar reactions in one pot, employing tetrathiafulvalene (TTF, **4a**, Scheme 1) as electron donor. A number of functionalised heterocycles [5–17] such as dihydrobenzofurans, indolines and indoles have been synthesized using this methodology and the radical-polar methodology has been employed successfully in the total synthesis [10] of aspidospermidine (**15**), the alkaloid of the *Aspidosperma* genus (Scheme 2). In line with our interests in generating aryl radicals by reduction of arenediazonium salts with tetrathiafulvalenes [5–16] **4** and dithiadiazafulvalenes [16–17] (DTDAF) **6** (Scheme 1) and later by electrochemical means [18], we were keen to compare the outcomes of these reactions of diazonium salts with those arising from the use of alternative neutral organic electron donors [19–20]. An interesting member of these alternative reagents is the commercially available and economically attractive tetrakis(dimethylamino)ethylene (TDAE, **1**). This paper describes the results of our investigations on reactions of TDAE as a neutral organic electron donor with arenediazonium salts.

TDAE (**1**), has been widely exploited as a strong electron donor [21–59] to electron-poor aliphatic and benzylic halides, notably those derived from organofluorine sources. Burkholder, Dolbier and Médebielle reported [28] that the electrochemical oxidation of TDAE in acetonitrile occurs in two reversible one-electron oxidation steps, to TDAE^+•^ **2** and TDAE^++^ **3** at −0.78 V and −0.61 V *vs* saturated calomel electrode (SCE). Recently, TDAE-promoted reduction of electron-deficient *o*- and *p*-nitrobenzyl chlorides [44–47], 1,2-bis(bromomethyl)arenes [48], mono and trichloromethyl azaheterocycles [49–50], 2-(dibromomethyl)quinoxaline [51], α-bromoketones [52] have been reported. Vanelle and co-workers recently reported a photoinduced reduction of *p*-nitrobenzaldehyde in the presence of TDAE [53].

The utility of TDAE as a strong electron donor in specific organometallic reactions, such as the chromium-mediated allylation of aldehydes and ketones [54–55] and the palladium-catalyzed reductive homo-coupling of aryl halides to afford the corresponding biaryls [56–59] illustrate further versatility of the reagent.

The fact that formation of aryl radicals had never been reported using TDAE meant that we were keen to compare its reactions with those of the structurally related TTF (**4a**). Thus, as shown in Scheme 2, the radical-cation of TTF intercepts intermediates with the formation of C-S bonds in the radical-polar crossover reaction; would the analogous chemistry be seen with TDAE where no sulfur atoms are present?

The experiments were of heightened interest because of the recent report by Andrieux and Pinson [60] on the standard reduction potential of the phenyl radical (formed by electrochemical reduction of the arenediazonium cation) to the phenyl anion (+0.05 V vs SCE). Thus, it had long been noted that cyclic voltammetry of aryl halides, particularly iodides, can give rise to a single two-electron wave in the reductive part of the cycle. The first electron converts the aryl iodide to the corresponding aryl radical, while the second electron transforms the aryl radical to an aryl anion. The two-electron single reductive wave arises because the transfer of the second electron is easier than the first. Andrieux and Pinson reasoned that in order to determine the potential for the conversion of aryl radical to aryl anion, a substrate other than an aryl halide would need to be used. As the one-electron reduction of an arenediazonium salt occurs [60] at much more positive potentials (*E*_p_ 0.16 V vs SCE) than for aryl iodides [61] (*E*_p_ −2.2 V vs SCE), this gives a much better chance to observe a second reductive peak in cyclic voltammetry and to determine the potential for conversion of aryl radicals to aryl anions. In the event, their study [60] showed two reductive peaks for benzenediazonium tetrafluoroborate (*E*_p_ 0.16 V and −0.64 V vs SCE). Through detailed analysis, Andrieux and Pinson showed that this second peak was consistent with reduction of the aryl radical to aryl anion and derived a value for the *standard* potential of this step as *E*^0^ = +0.05 V.

The reduction potentials determined by Andrieux and Pinson would be consistent with the chemistry that we had observed using TTF, in that TTF had been able to achieve the easier step of reducing arenediazonium salts to aryl radicals, but not the more difficult step (aryl radicals to aryl anions). The redox potentials associated with TTF are +0.32 V and +0.71 V vs SCE [62] so, even transferring one electron to the diazonium salts would superficially appear difficult, but it is well known that electron-transfer by a mediator in solution [63] is frequently more easily achieved (less negative potential) than would be expected from the bare electrochemical data. In the light of these facts, and given that TDAE is a much more powerful donor than TTF (by about 1.1 V for the transfer of the first electron), there is a danger that aryl radicals formed from arenediazonium salts using this reagent would be further converted into aryl anions, if the second electron transfer were sufficiently rapid. Therefore, we proposed to examine cyclization reactions of aryl radicals produced in this way to investigate this point.

## Results and Discussion

### Preparation of indolines

**Scheme 3:**
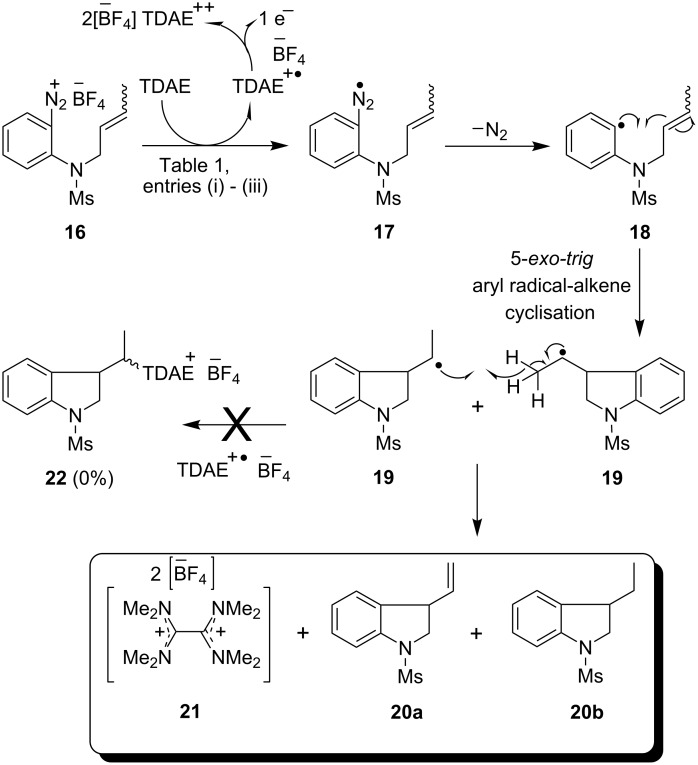
Studies on the reductive radical cyclization of arenediazonium salt **16** by TDAE.

Our initial studies reacted TDAE with simple arenediazonium salt **16** (Scheme 3). On addition of TDAE to a solution of the arenediazonium tetrafluoroborate salt **16** in acetonitrile [Table 1, entries (i) and (ii)], or, alternately, on addition of the arenediazonium salt to excess TDAE (2.5 equiv) [Table 1, entry (iii)] the reaction mixture underwent effervescence as it turned from deep red to pale orange. In each case, the reaction yielded an inseparable mixture of indolines **20a** and **20b** in approximately equal yield. In entry (iii) of that table, these yields were estimated from a calibrated NMR determination; following this, the mixture was subjected to epoxidation with *m*CPBA, leading to isolation of **20b** and the epoxide **20c** (not shown in Scheme 3) derived from **20a**. From this series of experiments, the oxidized product of TDAE, namely octamethyloxamidinium bis(tetrafluoroborate) (**21**) was isolated in up to 64% yield as an off-white powder. The structure of the salt **21** was characterized by NMR studies and also by mass spectrometry.

**Table 1:** Reductive radical cyclization of arenediazonium salt **16** by TDAE.

Entry	Equivalents of TDAE	Solvent	Temperature (°C)	Time	Yield (%) of		
					**20a**	**20b**	**21**

(i)	1.0	Acetonitrile	25	24 h	11	11	50
(ii)	1.0	Methanol	−40 to 25	24 h	4	5	64
(iii)	2.5	Acetonitrile	25	10 min	12	17	–^a^

^a^[TDAE]^++^ 2[BF_4_^−^] salt **21** was not isolated from the reaction

The formation of the indolines **20a** and **20b** could then be envisaged through an intermolecular radical disproportionation reaction of two cyclised radical intermediates **19** as explained in Scheme 3. No evidence was seen for the formation of salt **22** although the yields of the products **20** were not high. Non-observation of **22** illustrates that TDAE^+•^, unlike TTF^+•^, does not provide an efficient termination of radical processes, and the low yields of isolated compounds could be consistent with radical chemistry where efficient termination was lacking. With this as guidance to our thinking, the remaining substrates below were designed to provide internal termination routes for the radical chemistry.

**Scheme 4:**
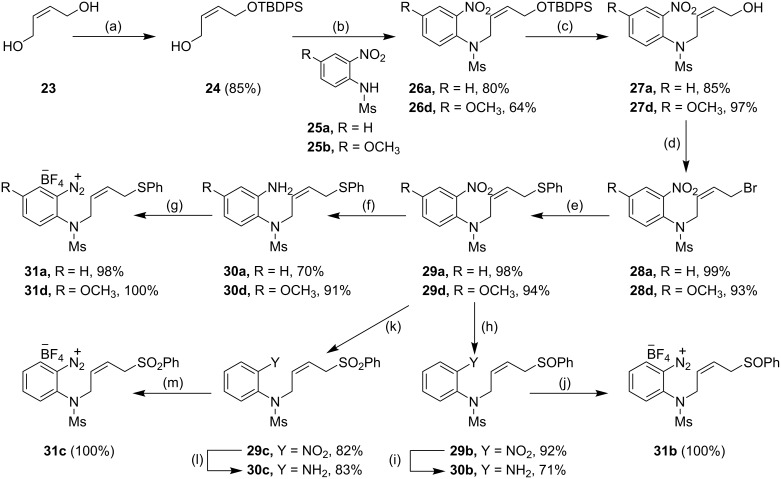
Preparation of the arenediazonium salts **31a**–**d**. *Reagents and conditions*: (a) **23**, NaH, THF, 0 °C, 0.5 h, then TBDPS-Cl, 0 °C to 25 °C, 4 h, 85%; (b) **25**, DIAD, PPh_3_, THF, 0 °C to 25 °C, 12 h, 80% (**26a**), 64% (**26d**); (c) TBAF, THF, 25 °C, 1.5 h, 85% (**27a**), 0.5 h, 97% (**27d**); (d) PBr_3_, DCM, 0 °C to 25 °C, 1 h, 99% (**28a**), 93% (**28d**); (e) PhSH, NaH, THF, 0 °C to 25 °C, 1 h, then **28**, 25 °C, 5 h, 98% (**29a**), 94% (**29d**); (f) Cu(acac)_2_, NaBH_4_, EtOH, 25 °C, 15 h, 70% (**30a**), SnCl_2_·2H_2_O, MeOH, 65 °C, 4 h, 91% (**30d**); (g) NOBF_4_, CH_2_Cl_2_, −20 °C to −10 °C, 1.5 h, 98% (**31a**), 100% (**31d**); (h) NaIO_4_, MeOH/H_2_O, r.t., 1 h 15 min, 92%; (i) SnCl_2_, MeOH, 65 °C, 4 h, 71%; (j) NOBF_4_, CH_2_Cl_2_, −20 °C to −10 °C, 1.5 h, 100%; (k) NaIO_4_, MeOH/H_2_O (1:1), r.t., 72 h, 82%; (l) SnCl_2_·2H_2_O, MeOH, 3.5 h, 83%; (m) NOBF_4_, CH_2_Cl_2_, −20 °C to −10 °C, 1.5 h, 100%.

One way to achieve clean termination of the radical process would be by providing a radical leaving group adjacent to the cyclised radical **19**. Appropriate groups might be sulfide, sulfoxide and sulfonyl groups [64–65]. Accordingly, arenediazonium salts **31a**–**d** were prepared bearing appropriate terminal radical leaving groups (Scheme 4) and treated with 1 equivalent of TDAE under different solvent conditions and temperature. As expected, the aryl radical generated from the reduction of the arenediazonium salts, underwent facile self-terminating 5-*exo*-*trig* aryl radical-alkene cyclization to afford the indolines **20a**, **32** as the sole products in very high yields (Table 2). Owing to the sensitive nature of the arenediazonium salts **31a**–**d**, they were usually generated *in situ* from the corresponding amines **30a**–**d** by treatment with nitrosonium tetrafluoroborate. One of the notable features of these cyclizations is the ease of purification of the product from the reaction mixture. The oxidized product of TDAE, namely octamethyloxamidinium bis(tetrafluoroborate) (**21**), was easily removed either by filtration or by simple work-up with water.

**Table 2:** Reductive radical cyclization of arenediazonium salts **31a**–**d** by TDAE.

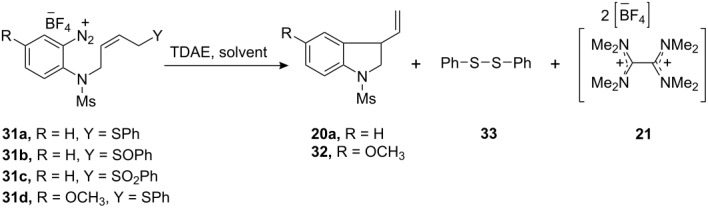								
Entry	Diazonium Salt	Equivalents of TDAE	Solvent	Temperature (°C)	Time	Isolated Yield (%) of		
						**20a**/**32**	**33**	**21**

(i)	**31a**	1.0	Acetonitrile	25	5 min	88	82	12
(ii)^a^	**31a**	0	Acetonitrile	25	5 min	0	0	0
(iii)^b,d^	**31a**	3.0	Acetonitrile	25	5 min	85	39	–^c^
(iv)	**31a**	1.0	Acetone	25	5 min	84	78	17
(v)	**31a**	1.0	Methanol	0 to 25	2 h	81	34	71
(vi)^d^	**31b**	1.0	Acetone	25	5 min	81	0	24
(vii)^d^	**31c**	1.0	Acetone	25	5 min	63	0	41
(viii)^d^	**31d**	1.0	Acetone	25	5 min	88	55	35

^a^Control reaction performed in the absence of TDAE reagent. ^b^This experiment was conducted by adding a solution of the diazonium salt in dry MeCN to a solution of TDAE in dry MeCN, while the other experiments in this table all featured addition of the TDAE to the diazonium salt. ^c^[TDAE]^++^ 2[BF_4_^−^] salt **21** was not isolated from the reaction. ^d^In these experiments, the arenediazonium salts **31a**–**d** were made *in situ* from their corresponding amines **30a**–**d**, while all the other experiments in this table featured direct use of arenediazonium salt.

### Cascade cyclizations

**Scheme 5:**
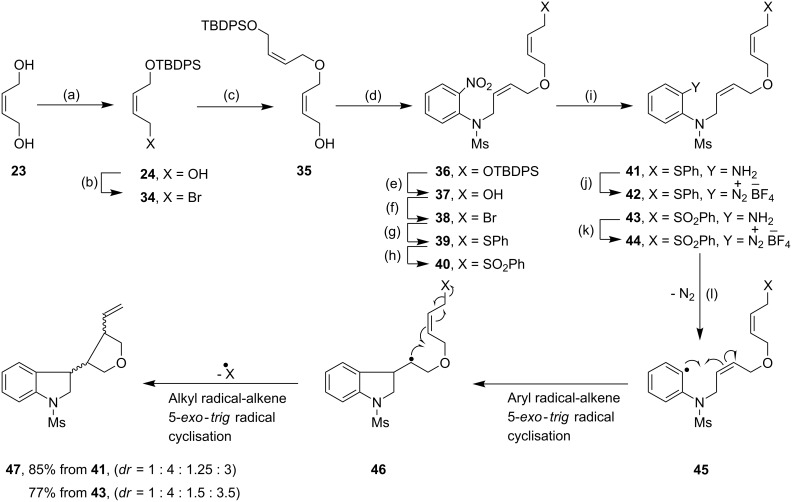
Cascade radical cyclizations of arenediazonium salts **42** and **44** by TDAE. *Reagents and conditions*: (a) **23**, NaH, THF, 0 °C, 0.5 h, then TBDPS-Cl, 0 °C to 25 °C, 4 h, 85%; (b) NBS, Me_2_S, CH_2_Cl_2_, −30 °C, 0.5 h, then **24**, −30 °C to r.t., 3 h, 95%; (c) **23**, NaH, THF, 0 °C to r.t., 1 h, then **34**, 72 h, 61%; (d) *N*-(2-nitrophenyl)methanesulfonamide (**25a**), DIAD, PPh_3_, THF, 0 °C to r.t., 1 h, 98%; (e) TBAF, THF, r.t., 20 min, 90%; (f) NBS, PPh_3_, CH_2_Cl_2_, −25 °C, 20 min, then **37**, −25 °C to r.t., 40 min, 96%; (g) PhSH, NaH, THF, 0 °C to r.t., 1 h, then **38**, r.t., 12 h, 86%; (h) NaIO_4_, H_2_O, MeOH, r.t., 76 h, 73%; (i) SnCl_2_, CH_3_OH, H_2_O, reflux, 3.5 h to 4 h, 95% (**41**), 78% (**43**); (j) NOBF_4_, CH_2_Cl_2_, −15 °C to 0 °C, 1.5 h; (k) NOBF_4_, CH_2_Cl_2_, −15 °C to 0 °C, 1.5 h; (l) TDAE (1.0 equiv), acetone, 10 min, r.t., 85% **47**, 46% **33**, 52% **21** in two steps, from **41**; 77% **47**, 46% **21** in two steps, from **43**.

To determine the scope of the TDAE-mediated reduction of arenediazonium salts, we sought to extend this methodology to more complex substrates, namely **42** and **44**. Pleasingly, the arenediazonium salts **42** and **44**, prepared *in situ* from the amines **41** and **43** respectively upon treatment with 1 equivalent of TDAE, underwent facile cascade radical cyclizations to afford the bicyclized product **47** in 85% and 77% yield respectively (Scheme 5). The ability of TDAE to mediate such efficient cascade cyclizations *via* two C-C bond formations reactions in one pot from the aryl radical **45** was significant considering the fact that our previous studies on similar substrates by TTF [5,8,16–17] and TMTTF [16] had shown competitive trapping of the intermediate alkyl radical **46** by TTF^+•^ or TMTTF^+•^.

### Preparation of indoles

**Scheme 6:**
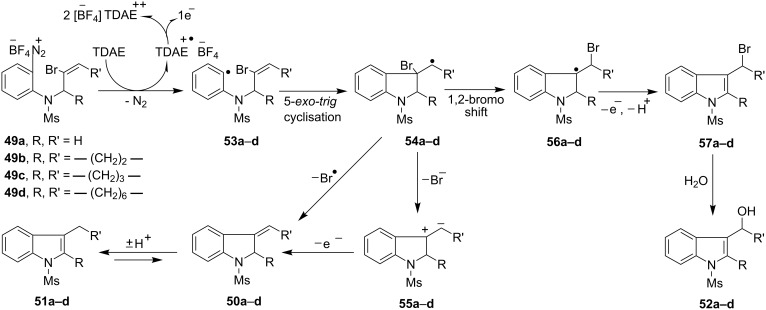
TDAE-mediated radical based addition-elimination route to indoles.

**Scheme 7:**
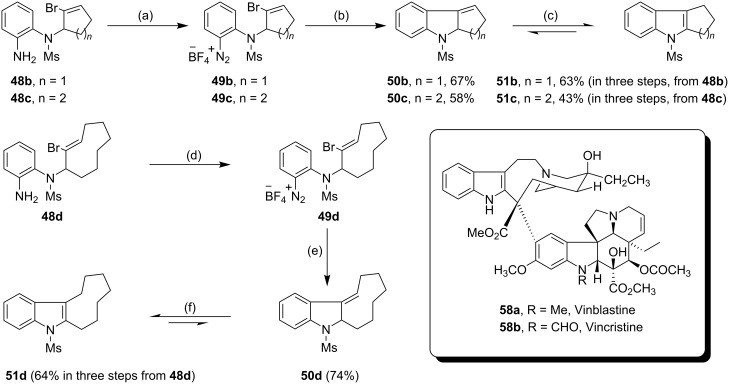
Cyclization of the arenediazonium salts **49b**–**d** by TDAE. *Reagents and conditions*: (a) NOBF_4_, CH_2_Cl_2_, −10 °C to 0 °C, 1.5 h; (b) TDAE (1.5 equiv), anhydrous DMF, r.t., 10 min; (c) *p*-toluenesulfonic acid monohydrate, CH_2_Cl_2_, r.t., 12 h, 63% (**51b**, in three steps from **48b**), 43% (**51c**, in three steps from **48c**); (d) NOBF_4_, CH_2_Cl_2_, −10 °C to 0 °C, 1.5 h; (e) TDAE (1.5 equiv), anhydrous DMF, r.t., 10 min, 74% **50d**; (f) *p*-toluenesulfonic acid monohydrate, CH_2_Cl_2_, r.t., 12 h, 64% (overall yield in three steps, from **48d**).

Following the successful implementation of the methodology on the synthesis of indolines, we next sought to harness the aryl radicals in the synthesis of indoles by a radical-based addition-elimination strategy [66–67]. However, our initial attempts in this area upon cyclization of arenediazonium salt **49a** were not fruitful as the reactions afforded a mixture of the exocyclic alkene **50a** and the alcohol **52a** [Table 3, entry (i)]. We expected that the aryl radical **53a** generated by the reduction of arenediazonium salt **49a** by TDAE would undergo 5-*exo*-*trig* radical cyclization onto the vinyl bromide to afford the alkyl radical intermediate **54a**, from which Br^•^ would be eliminated to afford the exocyclic alkene **50a** (Scheme 6). Such alkenes tautomerise easily to the corresponding indoles (in this case **51a**) in the presence of a trace of acid. Alcohol **52a** can arise by 1,2-bromine shift [66,68–69] from radical **54a** followed by either (a) loss a hydrogen atom from the resulting benzylic radical **56a** in collision with another radical or, less likely, (b) oxidation of **56a** by electron transfer to an arenediazonium cation. Loss of a proton from the cation so formed would yield bromoalkylindole **57a** and subsequent hydrolysis would result in the alcohol **52a**. However, when the same reaction was re-examined in anhydrous DMF as the solvent with 1.5 equivalents of TDAE, it afforded the unstable exocyclic alkene **50a** as the sole product, which after treatment with *p*-toluenesulfonic acid tautomerised to the indole **51a** in an overall 68% yield in three steps from **48a**. Adopting the optimized procedure, the diazonium salts **49b**–**d** on treatment with 1.5 equivalents of TDAE in anhydrous DMF yielded the indoles **51b**–**d** in 63%, 43% and 64% yields (in three steps from the corresponding aryl amines **48b**–**d**) respectively (Scheme 7). The indole **51d** bearing a fused 9-membered ring was of particular interest to us because the important anticancer drugs vinblastine (**58a**) and vincristine (**58b**) contain such a system.

**Table 3:** Initial optimization studies of cyclization of arenediazonium salt **49a**.

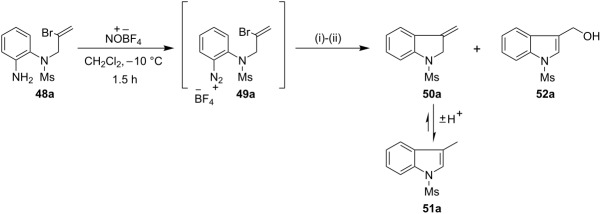							
Entry	Diazonium Salt	Equivalents of TDAE	Solvent	Temperature (°C)	Time	Isolated Yield^a^ (%) of	
						**51a**	**52a**

(i)	**49a**	1.0	Acetone	25	30 min	39^b^	40
(ii)	**49a**	1.5	DMF (anhydrous)	25	10 min	68^c^	0

^a^All isolated yields were calculated on the basis of the quantity of the starting aryl amine **48a**. ^b^The product **51a** was isolated directly from the reaction mixture after auto-tautomerisation of **50a** to indole **51a** during storage of reaction mixture prior to flash chromatography. ^c^The product **51a** was obtained by treatment of the intermediate exocyclic alkene **50a** with *p*-toluenesulfonic acid monohydrate in dichloromethane at r.t. for 12 h.

### Aryl C-C bond formation

**Scheme 8:**
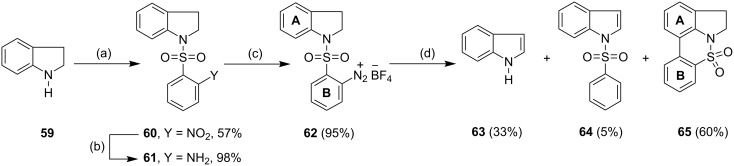
Cyclization of the arenediazonium salt **62** by TDAE. *Reagents and conditions*: (a) 2-Nitrobenzenesulfonyl chloride, DMAP, pyridine, 0 °C then 110 °C, 27 h, 57%; (b) H_2_, Pd/C, EtOAc, 3 h, 98%; (c) **61**, NOBF_4_, CH_2_Cl_2_, −10 °C, 1.5 h, 95%; (d) TDAE, CH_3_OH/acetone (1:1), 25 °C, 10 min, 33% **63**, 5% **64**, 60% **65**.

As a final extension of this methodology, we probed the feasibility of this methodology in aryl-aryl C-C bond formation reactions. Accordingly, the diazonium salt **62** was prepared from indoline (**59**), and treated with one equivalent of TDAE in acetone as solvent. The reaction mixture instantaneously turned deep red, with accompanying effervescence of nitrogen, and afforded the tetracyclic sulfonamide **65** in 60% yield along with indole (**63**) and indole sulfonamide **64** in 33% and 5% yield respectively (Scheme 8).

**Scheme 9:**
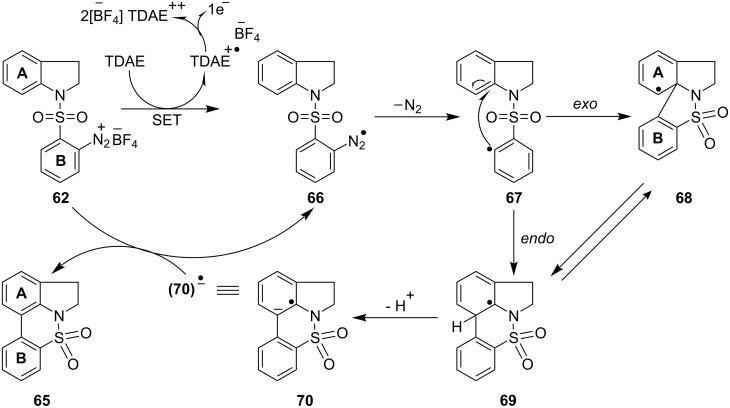
Mechanism for the formation of the tetracyclic sulfonamide **65**.

Initial SET from TDAE to the arenediazonium salt **62** afforded the aryl radical **67**, with release of molecular nitrogen. The aryl radical **67** would be expected [3,11,70] to cyclise onto the aryl ring **A** either through 5-*exo* or 6-*endo* cyclization. The radicals **68** and **69** could interconvert. Alternatively, aryl radical **67** could also undergo direct formation of the radical **69**. Rearomatization from **69** might then occur through a number of pathways; in one of these, the radical intermediate **69** would lose a proton to yield the radical anion **70** which, upon oxidation by loss of single electron to the starting diazonium salt **62**, would result in the formation of the tetracyclic sulfonamide **65** (Scheme 9).

**Scheme 10:**
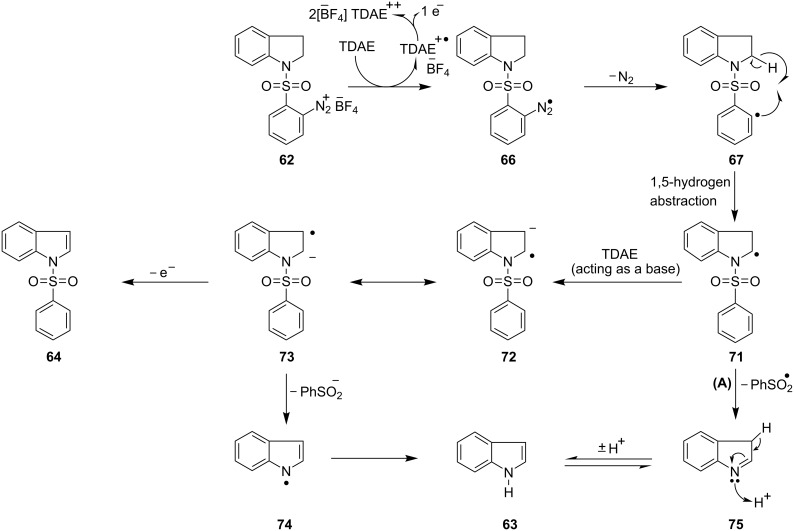
Possible mechanism for the formation of indole (**63**) and indole sulfonamide **64**.

Indole (**63**) and indole sulfonamide **64** can be formed *via* the indolinyl radical intermediate **71** (Scheme 10). The formation of the indolinyl radical **71** could be envisaged by abstraction of the hydrogen atom (1,5-hydrogen translocation) by the aryl radical **67** from the carbon atom in the α-position to the nitrogen atom of the indoline nucleus within the same molecule. The indolinyl radical **71** might follow pathway **A** and undergo radical fragmentation to the intermediate **75** which would eventually tautomerise to indole (**63**). The precedent for this radical fragmentation of the sulfonyl group comes from the previous work of our group [71], where a similar indolinyl radical underwent a radical cleavage of N-S bond to eliminate the sulfonyl group.

Indole sulfonamide **64** could be explained by deprotonation of radical **71** by TDAE to form radical-anion **72**, followed by electron loss. Alternatively, removal of hydrogen atom through reaction with another radical could afford **64**.

## Conclusion

We have reported a mild and direct method for generation of aryl radicals by reduction of arenediazonium salts using TDAE as a neutral ground-state organic electron donor. Additionally, we have described the utility of the aryl radicals in the construction of indolines and indoles by intramolecular radical cyclization of aryl radicals onto appropriately placed alkenes bearing terminal radical leaving groups. The presence of a suitable radical leaving group like a sulfide, sulfoxide or sulfone is necessary for the self-termination of the 5-*exo*-*trig* radical cyclization reactions to avoid competing intermolecular radical side-reactions. The TDAE-mediated radical-based addition-elimination route for the construction of indole ring systems warranted anhydrous reaction conditions for greater efficiency. A preliminary study on TDAE-mediated aryl-aryl C-C bond formation reaction has also been discussed. TDAE possesses a distinct advantage over other organic reducing agents as the oxidized products of TDAE are water soluble – thus the purification process is highly convenient. Further extensions of this methodology in the construction of several heterocyclic ring systems and complex synthetic targets for natural product synthesis are currently in progress in our laboratory.

## Supporting Information

File 1 Experimental data
