# Supplementary material for: Reduction of arenediazonium salts by tetrakis(dimethylamino)ethylene (TDAE): Efficient formation of products derived from aryl radicals
Source: Beilstein J Org Chem. 2009 Jan 12;5:1. doi: 10.3762/bjoc.5.1 (PMC2649425; doi:10.3762/bjoc.5.1)
Supplement: File 1 — Experimental data [file Beilstein_J_Org_Chem-05-01-s001.doc]

**Supporting Information File 1**

# Beilstein Journal of Organic Chemistry

# Beilstein Journal of Organic Chemistry

# Supporting Information to Accompany:

Reduction of arenediazonium salts by tetrakis(dimethylamino)ethylene (TDAE): Efficient formation of products derived from aryl radicals

Mohan Mahesh^1^, John A. Murphy^*,1^, Franck LeStrat^1^ and Hans Peter Wessel^2^

Address: WestCHEM, Department of Pure and Applied Chemistry, University of Strathclyde, 295, Cathedral Street, Glasgow G1 1XL, U.K. and ^2^Pharma Research, Discovery Chemistry, F. Hoffmann-La Roche Ltd., Basel, CH-4070, Switzerland

Email: John A. Murphy^*^ *-* [john.murphy@strath.ac.uk](mailto:john.murphy@strath.ac.uk)

* Corresponding author

| **Contents List** | **Page No.** |
| --- | --- |
| Table of Contents | S1 |
| Materials and Methods | S2–S4 |
| Experimental and Spectral Data | S4–S61 |
| References for the Experimental Section | S61 |

**Materials and Methods:**

Unless otherwise stated, all reactions were performed in flame-dried apparatus under a nitrogen or argon atmosphere using dry and deoxygenated solvents. A glove box (System One Glove Box, Innovative Technology Inc., U.S.A.) was used to handle extremely air-sensitive and moisture-sensitive reagents and reactions under a nitrogen atmosphere (oxygen and moisture levels were maintained at 0–2 ppm at all times). All reagents of highest available purity were purchased from commercial suppliers and used as received without further purification unless otherwise stated. As required, organic solvents were dried and/or distilled prior to use. Tetrahydrofuran was dried by distillation from sodium and benzophenone, dichloromethane was dried by distillation over calcium hydride, diethyl ether was dried over sodium wire, toluene was dried by distillation from sodium, petroleum ether was distilled at 40–60 °C and acetonitrile was dried by distillation over calcium hydride. Alternatively, tetrahydrofuran, dichloromethane, hexane, diethyl ether and toluene were dried and deoxygenated with a Pure-Solv 400 solvent purification system by Innovative Technology Inc., U.S.A. and the moisture content of the solvents was constantly monitored by employing standard methods by Karl Fischer coulometer (METTLER TOLEDO DL39). Anhydrous DMF was purchased commercially from Sigma-Aldrich Chemical Company and the residual moisture content in it (34 to 100 ppm of H_2_O level) was analyzed using standard methods by Karl Fischer coulometer (METTLER TOLEDO DL39). In experiments where sodium hydride (60% suspension in mineral oil) has been utilised as a base, it was washed with dry hexane or THF at least twice prior to use. Organic extracts were, in general, dried over anhydrous sodium sulfate (Na_2_SO_4_) or anhydrous magnesium sulfate (MgSO_4_).

Flash chromatography was performed using BDH silica gel 60 (particle size 33 µm to 70 µm, BDH 153325P). Thin layer chromatography (TLC) was performed using aluminium sheets of silica gel 60 F_254_ and was visualized under Mineralight UVGL-58 lamp (254 nm). The TLC plates were developed with aqueous potassium permanganate solution, methanolic phosphomolybdic acid (10–20% w/v), acidic *p*-anisaldehyde (10% v/v) or acidic methanolic vanillin solutions.

High performance liquid chromatography (HPLC) was performed using Gilson Model 302 pump, Gilson 802C manometric module, Milton Roy Spectrometer detector (λ = 257 nm) and a Kromasil Silica column (semi-preparative: 10 μm pore size, 100 Å particle size, 250 × 10.0 mm dimension) at a flowrate of 2 ml/min.

All Gas Chromatography Mass Spectrometry (GC–MS) was performed on Thermo Finnigan PolarisQ Ion Trap Mass Spectrometer/Trace GC instrument with ZB-5 column (30 m), at 1ml/min He gas flow rate and temperature range of 50 to 320 °C with an increment of 10 to 20 °C/min.

High and low resolution mass spectra were recorded at the EPSRC National Mass Spectrometry Service Centre, Swansea on a JLZX 102, VGZAB-E or a VG micromass instrument. The spectra were recorded using electron impact (EI), chemical ionization (CI), fast atom bombardment (FAB) or electrospray ionization (ESI) techniques as stated for each compound.

Infra-red spectra were recorded on Perkin Elmer Spectrum One FT-IR spectrometer and are reported in frequency of absorption (cm^−−1^). Melting points (mp) of solid compounds were determined by Reichert melting point apparatus or Gallenkamp melting point apparatus and are uncorrected.

Proton NMR (^1^H) spectra were recorded at 400.13 MHz on a Bruker DPX400 or 400.13 MHz on a Bruker AMX400 or 400.20 MHz on a Bruker AV400 or 400.03 MHz on a Bruker AV400 NMR spectrometer. Carbon NMR (^13^C) spectra were similarly recorded at 100.61 MHz on a Bruker DPX400 or 100.63 MHz on a Bruker AV400 or 100.61 MHz on a Bruker AMX400 NMR spectrometer using a broadband decoupled mode. Using a DEPT or JMOD sequence the ^13^C NMR spectroscopic signals were assigned to CH_3_, CH_2_, CH and C. The two-dimensional (2D) homonuclear (^1^H-^1^H) shift correlation experiments were carried out at 400.13 MHz on a Bruker DPX400 or 400.13 MHz on a Bruker AMX400 NMR spectrometer. The 1D ^1^H NOE NMR spectroscopic data and two-dimensional ^1^H NOESY NMR spectroscopic data were acquired at 400.13 MHz on a Bruker AMX400 NMR spectrometer using a Dual (BBO) probehead at a temperature of 300 K. The NMR experiments were, in general, carried out in deuterochloroform (CDCl_3_), unless, otherwise specified. The chemical shifts (*δ*) are quoted in parts per million (ppm) relative to the signals attributed by the residual protonated solvent present in the deuterated solvent. Multiplicities are abbreviated as s, singlet; d, doublet; t, triplet; q, quartet; m, multiplet; br, broad. The coupling constants (*J*) are reported in Hertz (Hz). In cases, where superimposition of the signals of two, or more, isomers or rotamers occurred, the signals have been reported as multiplets (m), unless the coupling constants of each isomer or rotamer could be ascertained. All NMR data were processed offline on a Dell Precision 340 Workstation operating under Windows 2000 Professional or Windows XP Professional using XWIN-NMR 3.0 or XWIN-NMR 3.5 or TopSpin® 2.0 processing software packages.

The IUPAC names of some compounds were obtained using ChemDraw Ultra version 8.0 or Beilstein’s AutoNom Version 4.0 software.

**Experimental Procedure:**

**Experimental Section I**

**Tetrakis(dimethylamino)ethylene (TDAE, 1)** [1]

Tris(dimethylamino)methane (4.067 g, 28.0 mmol) was placed in a dry 10-ml, round-bottom flask equipped with a reflux condenser (flame-dried under vacuum and purged with argon prior to use). It was degassed by passing a stream of argon gas through it for 5 min at r.t. The reaction flask was immersed in an oil bath and the reaction was kept at reflux for 72 h under a small pressure of dry argon, by adjusting the temperature upward between 180 °C and 190 °C as the reaction proceeded and the reaction mixture turned dark-brown. When the reaction was over, the condenser was carefully exchanged for a small distilling head and the product was distilled at 50 °C at 1 mm Hg pressure to yield tetrakis(dimethylamino)ethylene [1] (**1**, 4.098 g, 73%) as a pale yellow-green liquid. The sample of tetrakis(dimethylamino)ethylene thus obtained was stored under an argon atmosphere; ^1^H NMR (400.13 MHz, C_6_D_6_) *δ* 2.66 (s, 12H); ^13^C NMR (100.61 MHz, C_6_D_6_) *δ* 41.6 (CH_3_), 131.9 (C). The physical characteristics and ^1^H NMR spectroscopic data of the sample of TDAE (**1**) coincided well with those reported in the literature [1].

**Preparation of the diazonium salt 16**

**Scheme 11:** Preparation of the arenediazonium salt **16.** *Reagents and conditions:* (i) MeSO_2_Cl, DMAP, pyridine, 116 °C, 27 h, 33% (**25a**), r.t., 18 h, 54% (**25b**); (ii) CH_3_-CH=CH-CH_2_OH, DIAD, PPh_3_, THF, 0 °C then r.t., 12 h, 99% **77**; (iii) NaBH_4_, Cu(acac)_2_, EtOH, 3 h, 87% **78**; (iv) NOBF_4_, CH_2_Cl_2_, −10 °C, 1.5 h, 100% **16**.

***N-*(2-Nitrophenyl)methanesulfonamide (25a)** [2,3]

Methanesulfonyl chloride (18.58 ml, 240.0 mmol, 1.2 equiv) was added dropwise under a nitrogen atmosphere at 0 °C over a period of 30 min, to a solution of 2-nitroaniline (**76a**, 27.625 g, 200.0 mmol, 1.0 equiv) and 4-(dimethylamino)pyridine (2.443 g, 20.0 mmol, 0.1 equiv) in pyridine (150 ml). The mixture was stirred at 0 °C for 30 min and then was warmed to r.t. and heated under reflux for 27 h at 116 °C. After cooling, the mixture was poured into hydrochloric acid (2 M, 150 ml) and further acidified with concentrated hydrochloric acid. The aqueous phase was then extracted with dichloromethane (3 × 150 ml) and then the combined organic phase was extracted with sodium hydroxide (2 M, 4 × 150 ml). The aqueous solution was acidified until pH 2 with concentrated hydrochloric acid (a yellow solid was precipitated), and extracted with dichloromethane (3 × 150 ml). The combined organic portions were dried over anhydrous magnesium sulfate and the solvent was removed to give a brown solid. Recrystallisation from ethyl acetate and hexane gave *N-*(2-nitrophenyl)methanesulfonamide (**25a**) [2,3] as a yellow solid (14.313 g, 33%); mp 102–103 °C (lit. [3] mp 101–103 °C) [Found: M^+•^ (EI), 216.0203. C_7_H_8_N_2_O_4_S requires *M*, 216.0199]; [Found: C, 39.0; H, 3.8; N, 12.8. C_7_H_8_N_2_O_4_S requires C, 38.9; H, 3.7; N, 13.0%]; IR (KBr disc) 3267, 3122, 3020, 1615, 1579, 1531, 1485, 1375, 1344, 1271, 1172, 1147, 968, 913, 847, 740 cm^−1^; ^1^H NMR (400.13 MHz, CDCl_3_) *δ* 3.16 (s, 3H), 7.25 (ddd, *J* = 8.5, 8.5, 1.2 Hz, 1H), 7.70 (ddd, *J* = 8.5, 8.5, 1.6 Hz, 1H), 7.91 (dd, *J* = 8.5, 1.2 Hz, 1H), 8.28 (dd, *J* = 8.5, 1.6 Hz, 1H), 9.76 (s, 1H); ^13^C NMR (100.61 MHz, CDCl_3_) *δ* 41.0 (CH_3_), 119.8 (CH), 123.8 (CH), 126.9 (CH), 134.6 (2 × C), 136.5 (CH); *m/z* (EI) 216 (M^+•^, 62%), 138 (100), 108 (36), 91 (22). The spectroscopic data of *N-*(2-nitrophenyl)methanesulfonamide (**25a**) were consistent with those reported in the literature [2,3].

***N*-(4-Methoxy-2-nitrophenyl)methanesulfonamide (25b)**

Methanesulfonyl chloride (9.29 ml, 120.0 mmol, 1.2 equiv) was added dropwise to a solution of 4-methoxy-2-nitroaniline (**76b**, 16.815 g, 100.0 mmol, 1.0 equiv) and 4-(dimethylamino)pyridine (1.222 g, 10.0 mmol, 0.1 equiv) in pyridine (75 ml) under a nitrogen atmosphere at 0 °C. The reaction mixture was slowly warmed to r.t. and stirred for 18 h. The reaction mixture was concentrated by removing the solvent under reduced pressure, diluted with ethyl acetate (250 ml) and sequentially washed with hydrochloric acid (2 M, 4 × 400 ml), aqueous sodium hydroxide (2 M, 4 × 500 ml). The combined aqueous extracts were slowly acidified with concentrated hydrochloric acid at 0 °C until pH 2 to obtain a bright yellow precipitate which was extracted with ethyl acetate (300 ml). The combined organic extracts were dried over anhydrous magnesium sulfate, filtered, and evaporated to dryness to obtain *N*-(4-methoxy-2-nitrophenyl)methanesulfonamide (**25b**) [4] as brilliant yellow needles (13.410 g, 54%); mp 123–123.5 °C (lit. [4] mp 125 °C) [Found: MNH_4_^+^ (ESI), 264.0656. C_8_H_10_N_2_O_5_S requires *MNH_4_*, 264.0649]; IR (KBr disc) 3246, 3111, 2964, 2929, 1577, 1526, 1500, 1443, 1417, 1341, 1304, 1281, 1161, 1140, 1035, 975, 902, 829, 784, 750 cm^−1^; ^1^H NMR (400.13 MHz, CDCl_3_) *δ* 3.06 (s, 3H), 3.89 (s, 3H), 7.27 (dd, *J* = 9.2, 3.0 Hz, 1H), 7.71 (d, *J* = 3.0 Hz, 1H), 7.82 (d, *J* = 9.2 Hz, 1H), 9.20 (s, 1H); ^13^C NMR (100.61 MHz, CDCl_3_) *δ* 40.9 (CH_3_), 56.6 (CH_3_), 109.9 (CH), 123.6 (CH), 124.2 (CH), 127.5 (C), 138.6 (C), 156.5 (C); *m/z* (EI) 246 (M^+•^, 79%), 168 (69), 167 (100), 151 (16), 137 (80), 122 (15), 109 (33), 106 (44), 95 (11), 80 (36), 79 (84), 77 (27), 68 (38), 63 (33), 52 (69).

***N*-(But-2-enyl)-*N*-(2-nitrophenyl)methanesulfonamide (77)**

Diisopropyl azodicarboxylate (5.95 ml, 30.0 mmol, 1.5 equiv) was added dropwise over a period of 30 min, to a suspension of *N-*(2-nitrophenyl)methanesulfonamide (**25a,** 4.324 g, 20.0 mmol, 1.0 equiv), crotyl alcohol (1.86 ml, 22.0 mmol, 1.1 equiv) and triphenylphosphine (7.868 g, 30.0 mol, 1.5 equiv) in freshly distilled THF (120 ml) at 0 °C. The reaction mixture was allowed to warm to r.t. and stirred under nitrogen overnight. The reaction mixture was then concentrated to dryness, dissolved in dichloromethane (150 ml) and washed sequentially with aqueous sodium hydroxide (2 M, 3 × 150 ml), hydrochloric acid (2 M, 75 ml), saturated sodium bicarbonate solution (150 ml) and saturated brine solution (3 × 150 ml). The organic extracts were then dried over anhydrous magnesium sulfate and evaporated under reduced pressure to dryness. Column chromatography on silica using toluene as the eluant gave the desired compound as a yellow solid. Recrystallisation from ethyl acetate and hexane gave *N*-(but-2-enyl)-*N*-(2-nitrophenyl)methanesulfonamide (**77**) as yellow needles (5.360 g, 99%). The product was observed to be a 1:5 mixture of geometrical isomers by ^1^H NMR spectroscopy; mp 41–42 °C [Found: MNH_4_^+^ (ESI), 288.1015. C_11_H_14_N_2_O_4_S requires *MNH_4_*, 288.1013]; IR (KBr disc) 3020, 2935, 1607, 1534, 1488, 1454, 1361, 1336, 1158, 1098, 1038, 960, 928, 869, 842, 782 cm^−1^; ^1^H NMR (400.13 MHz, CDCl_3_) *δ* 1.59 (major) and 1.67 (minor) (2 × d, *J* = 4.2 and 6.2 Hz, 3H), 3.00 (major) and 3.01 (minor) (2 × s, 3H), 4.00 (minor) and 4.15 (major) (2 × broad s, 2H), 4.89–4.95 and 5.45–5.63 (2 × m, major + minor, 2H), 7.47 (m, 2H), 7.61 (ddd, *J* = 7.0, 7.0, 1.4 Hz, 1H), 7.86 (dd, *J* = 8.0, 1.4 Hz, 1H); ^13^C NMR (100.61 MHz, CDCl_3_) *δ* 17.7 (CH_3_), 40.3 (CH_3_), 53.8 (CH_2_), 124.9 (CH), 125.3 (CH), 129.5 (CH), 132.2 (C), 132.4 (CH), 133.37 (CH), 133.39 (CH), 148.9 (C); *m/z* (CI) 288 [(M + NH_4_)^+^, 100%], 270 (2), 241 (21), 161 (16).

***N*-(2-Aminophenyl)-*N*-(but-2-enyl)methanesulfonamide (78)**

Sodium borohydride (0.378 g, 10.0 mmol, 1.0 equiv) suspended in ethanol (100 ml) was flushed into a stirred solution of copper(II) acetylacetonate (0.524 g, 2.0 mmol, 0.2 equiv) in ethanol (500 ml) at 0 °C. The mixture was slowly brought to r.t. and stirred under nitrogen for 1 h until black flakes appeared in the clear solution. The solution of *N*-(but-2-enyl)-*N*-(2-nitrophenyl)methanesulfonamide (**77**, 2.703 g, 10.0 mmol, 1.0 equiv) in ethanol (100 ml) was added dropwise, followed by the addition of sodium borohydride (0.757 g, 20.0 mmol, 2.0 equiv). The reaction mixture was stirred under nitrogen for 3 h and the progress of the reaction was checked by thin layer chromatography. The reaction was then stopped by quenching with distilled water (100 ml). The organic solvent was then removed *in* *vacuo* and the residue was extracted with diethyl ether (3 × 100 ml). The combined organic extracts were then washed with ammonia solution (2 M, 3 × 100 ml), followed by saturated brine solution (3 × 100 ml). The ethereal layer was dried over anhydrous magnesium sulfate and the evaporated under reduced pressure. The residue was subjected to flash chromatography over silica gel [eluant: dichloromethane/toluene = 1:1] to afford *N*-(2-aminophenyl)-*N*-(but-2-enyl)methanesulfonamide (**78**) as a pale yellow solid (2.100 g, 87%); mp 77–78 °C [Found: M^+•^ (EI), 240.0950. C_11_H_16_N_2_SO_2_ requires 240.0927]; IR (KBr disc) 3469, 3374, 2930, 1621, 1502, 1326, 1315, 1157, 967, 955, 741 cm^−1^; ^1^H NMR (400.13 MHz, CDCl_3_) *δ* 1.65 (dd, *J* = 5.8, 1.0 Hz, 3H), 2.97 (s, 3H), 4.00–4.20 (broad s, 2H), 4.13 (d, *J* = 6.3 Hz, 2H), 5.47–5.65 (m, 2H), 6.72 (ddd, *J* = 7.9, 7.3, 1.4 Hz, 1H), 6.78 (dd, *J* = 8.1, 1.4 Hz, 1H), 7.09 (dd, *J* = 7.9, 1.4, 1H), 7.14 (ddd, *J* = 8.1, 7.3, 1.4 Hz, 1H); ^13^C NMR (100.61 MHz, CDCl_3_) *δ* 18.2 (CH_3_), 39.0 (CH_3_), 53.7 (CH_2_), 117.4 (CH), 118.8 (CH), 125.3 (C), 125.6 (CH), 129.4 (CH), 130.2 (CH), 131.9 (CH), 147.0 (C); *m/z* (EI) 240 (M^+•^, 39%), 161 [(M − SO_2_CH_3_), 100%].

**2-[(But-2-enyl)methanesulfonylamino]benzenediazonium tetrafluoroborate (16)**

A solution of *N*-(2-aminophenyl)-*N*-(but-2-enyl)methanesulfonamide (**78**) (0.961 g, 4.0 mmol, 1.0 equiv) in dry dichloromethane (15 ml) was added to a stirred suspension of nitrosonium tetrafluoroborate (0.700 g, 6.0 mmol, 1.5 equiv) in dry dichloromethane (35 ml) under nitrogen at −10 °C. After 1 h, additional nitrosonium tetrafluoroborate (0.233 g, 2.0 mmol, 0.5 equiv) was added to the reaction mixture and after 30 min the dichloromethane was removed under reduced pressure. The residue was dissolved in a little acetone and recrystallised from cold and dry diethyl ether between −10 °C and 0 °C, then filtered under nitrogen to give the diazonium salt **16** as a pale yellow solid which immediately turned into a brown semi-solid upon exposure to atmosphere (1.350 g, 100%). The diazonium salt **16** was dried by passing a stream of nitrogen gas over it, and was immediately stored under argon at –20 to −10 °C; IR (CH_2_Cl_2_) 3055, 2987, 2305, 1422, 896, 737 cm^−1^; ^1^H NMR (400.13 MHz, acetone-*d*_6_) *δ* 1.54 (dd, *J* = 6.4, 1.5 Hz, 3H), 3.24 (s, 3H), 4.56 (dd, *J* = 7.0, 1.0 Hz, 2H), 5.54–5.61 (m, 1H), 5.75–5.82 (m, 1H), 8.05 (ddd, *J* = 8.3, 7.7, 1.0 Hz, 1H), 8.33 (dd, *J* = 8.3, 1.0 Hz, 1H), 8.46 (ddd, *J* = 8.3, 7.7, 1.5 Hz, 1H), 8.91 (dd, *J* = 8.3, 1.5 Hz, 1H); ^13^C NMR (100.61 MHz, acetone-*d*_6_) *δ* 17.4 (CH_3_), 36.9 (CH_3_), 53.7 (CH_2_), 117.4 (C), 124.4 (CH), 131.4 (CH), 131.5 (CH), 134.2 (CH), 135.5 (CH), 143.0 (C), 143.5 (CH).

Initial studies on cyclization of the arenediazonium salt 16 by TDAE under different conditions

**Table 1:** Reductive radical cyclization of arenediazonium salt **16** by TDAE.

| **Entry** | **General Procedure** | **Equivalents of TDAE** | **Solvent** | **Temperature (**°C**)** | **Time** | Yield (%) of | | |
| --- | --- | --- | --- | --- | --- | --- | --- | --- |
|  |  |  |  |  |  | **20a*** | **20b*** | **21**** |
| (i) | A | 1.0 | Acetonitrile | 25 | 24 h | 11 | 11 | 50 |
| (ii) | A | 1.0 | Methanol | −40 to 25 | 24 h | 4 | 5 | 64 |
| (iii) | B | 2.5 | Acetonitrile | 25 | 10 min | 12 | 17 | 0 |

* The yields were determined by calibrated ^1^H NMR spectroscopic analysis of an inseparable mixture of **20a** and **20b** in CDCl_3_

** Isolated yield

**Representative General Procedure A [Table 1, entry (i)]:** A 25 ml two-necked round bottomed flask equipped with a magnetic stirring bar and a septum was flame-dried in vacuo, backfilled with dry argon gas and loaded with freshly distilled dry acetonitrile (20 ml). The solvent was degassed by purging with a stream of argon gas for 30 min at 25 °C and a pure sample of 2-[(but-2-enyl)methanesulfonylamino]benzenediazonium tetrafluoroborate (**16**, 0.650 g, 1.92 mmol, 1.0 equiv) was added to the solvent at r.t. under an argon atmosphere. TDAE (0.44 ml, 1.92 mmol, 1.0 equiv) was then added to the diazonium salt solution dropwise for a period of 5 min under an argon atmosphere at 25 °C. The solution turned deep red in colour immediately with evolution of bubbles of nitrogen and gradually turned to orange with precipitation of brown precipitate. The reaction mixture was vigorously stirred at room temperature for 24 h under an argon atmosphere. After this time, the TLC analysis (petroleum ether/ethyl acetate = 1:1) indicated that the starting diazonium salt was totally consumed with the formation of several new compounds. The turbid-brown solution was filtered, washed with dry ethyl acetate (3 × 10 ml) under an argon atmosphere and dried *in vacuo* to obtain octamethyloxamidinium bis(tetrafluoroborate) (**21**, 0.181 g, 50%) as an off-white powder, mp 230 °C (dec) and the organic filtrate was evaporated to dryness under reduced pressure to yield a brown semi-solid substance. This crude product was purified by flash chromatography on silica gel to obtain a clear colourless oil (0.094 g) which was found to be an equimolar mixture of 3-ethyl-1-methanesulfonyl-2,3-dihydro-1*H*-indole [5,6] (**20b**, 11% NMR yield) and 1-methanesulfonyl-3-vinyl-2,3-dihydro-1*H-*indole [7] (**20a**, 11% NMR yield) (eluant: petroleum ether/ethyl acetate = 17:3). Although the two components **20a** and **20b** could not be separated, the signals attributed to each component, from NMR spectroscopic analysis of the mixture along with their GC–MS data were consistent with those of each of the original pure samples given in the later part of this experimental section.

**Octamethyloxamidinium bis(tetrafluoroborate) (21):** [Found: (M − BF_4_)^+^ (ESI), 287.2025. (C_10_H_24_N_4_)^2+^ 2(BF_4_^─^) requires 287.2025]; IR (KBr disc) 1672, 1473, 1406, 1084, 1033, 870 cm^−1^; ^1^H NMR (400.13 MHz, D_2_O) *δ* 3.35 (s, 12H), 3.63 (s, 12H); ^13^C NMR (100.61 MHz, D_2_O) *δ* 43.0 (CH_3_), 43.9 (CH_3_), 156.6 (C); *m/z* (ESI) 287 [(M − BF_4_)^+^, 16%], 219 (11), 199 (26), 100 (100), 85 (3).

**Representative General Procedure B [Table 1, entry (iii)]:** A 25 ml two-necked round bottomed flask equipped with a magnetic stirring bar and a rubber septum was flame-dried in vacuo, backfilled with dry argon gas and transported to a glove box (oxygen and moisture levels were maintained at 0–2 ppm at all times) under a nitrogen atmosphere. TDAE (0.601 g, 3.0 mmol, 2.5 equiv) was added to the reaction vessel, followed by addition of a solution of 2-[(but-2-enyl)methanesulfonylamino]benzenediazonium tetrafluoroborate (**16**, 0.407 g, 1.2 mmol, 1.0 equiv) in dry, freshly distilled and deoxygenated acetonitrile (3 ml, degassed by purging with a stream of argon gas for 30 min at 25 °C prior to its transportation to a glove box). The reaction mixture turned deep red in colour immediately with evolution of bubbles of nitrogen which gradually turned to orange with precipitation of brown precipitate. The reaction mixture was vigorously stirred at 25 °C for 10 min inside the glove box under a nitrogen atmosphere. After this time, the reaction vessel was transported outside the glove box and the TLC analysis (petroleum ether/ethyl acetate = 1:1) indicated that the starting diazonium salt was totally consumed with the formation of several new compounds. The turbid-brown reaction mixture was evaporated to dryness under reduced pressure to yield a reddish-brown semi-solid substance. This crude product was then suspended in dry and degassed dichloromethane (3 ml) and *p*-toluenesulfonic acid (0.228 g, 1.2 mmol, 1.0 equiv) was added. The reaction mixture was stirred for 4 h under an argon atmosphere at 25 °C. The reaction mixture was quenched by addition of sodium bicarbonate solution (saturated, 20 ml) and the contents were then extracted with dichloromethane (75 ml). The organic layer was further washed with sodium bicarbonate solution (saturated, 3 × 75 ml), separated, dried over anhydrous sodium sulfate, filtered and then evaporated under reduced pressure to obtain a brown semi-solid which was purified by flash chromatography on silica gel to obtain a clear colourless oil (0.091 g) which was found to be an equimolar mixture of 3-ethyl-1-methanesulfonyl-2,3-dihydro-1*H*-indole [5,6] (**20b**, 17% NMR yield) and 1-methanesulfonyl-3-vinyl-2,3-dihydro-1*H-*indole [7] (**20a**, 12% NMR yield) (eluant: hexane/ethyl acetate = 17:3).

The NMR yields of the products **20a** and **20b** were determined from standardised ^1^H NMR spectroscopic analysis of a solution of the total amount of the isolated mixture of **20a** and **20b** (0.091 g) in CDCl_3_ (0.8 ml) along with acetophenone (0.0246 g, 0.2047 mmol) as an internal standard. The ^1^H NMR chemical shifts and the ratio of the corresponding integrals attributed to each component of the mixture were in accordance with ^1^H NMR spectroscopic data of the authentic samples of each of the compounds **20a**, **20b** and acetophenone (for ^1^H NMR spectroscopic data see below). Also the GC–MS data of the mixture of **20a** and **20b** were consistent with those of the authentic samples of **20a** and **20b**.

**^1^H NMR spectroscopic data of a commercial sample of acetophenone (internal ^1^H NMR standard):** ^1^H NMR (400.13 MHz, CDCl_3_) *δ* 2.60 (s, 3H), 7.40–7.50 (m, 2H), 7.52–7.60 (m, 1H), 7.87–8.05 (m, 2H).

**Epoxidation of the mixture containing 1-methanesulfonyl-3-vinyl-2,3-dihydro-1*H-*indole (20a)**

The inseparable mixture (0.091 g) of 3-ethyl-1-methanesulfonyl-2,3-dihydro-1*H*-indole (**20b**) [5,6] and 1-methanesulfonyl-3-vinyl-2,3-dihydro-1*H-*indole [7] [**20a,** obtained from entry (iii) of Table 1] was dissolved in dichloromethane (5 ml), cooled to 0 °C and *m*CPBA (0.0897 g of 77% sample of commercial *m*CPBA, 0.4 mol) was added to the solution. The mixture was brought to r.t. and stirred for 24 h under an argon atmosphere. The progress of the reaction was monitored by ^1^H NMR spectroscopic analysis. After 24 h, the mixture was cooled to 0 °C and additional *m*CPBA (0.0897 g of 77% sample of commercial *m*CPBA, 0.4 mol) was added to the reaction mixture. The mixture was warmed to r.t. and continued to stir under an argon atmosphere for additional 12 h (total time = 36 h). The mixture was then diluted with additional dichloromethane (50 ml) and washed with aqueous sodium sulfite solution (2 M, 3 × 50 ml) until a negative reaction with starch-KI paper was observed. The separated organic phase was then sequentially washed with sodium bicarbonate solution (saturated, 3 × 100 ml) and water (150 ml). The organic phase was separated, dried over anhydrous sodium sulfate, filtered and evaporated to dryness under reduced pressure to obtain a pale yellow semi-solid. Flash chromatography of the crude material on silica gel afforded 3-ethyl-1-methanesulfonyl-2,3-dihydro-1*H*-indole [5,6] [**20b**, 0.034 g, 13% isolated yield based on the amount of the starting arenediazonium salt **16** for entry (iii) of Table 1] as a pale yellow semi-solid (eluant: hexane/ethyl acetate = 17:3) and 1-methanesulfonyl-3-oxiranyl-2,3-dihydro-1*H*-indole (**20c**) as a colorless semi-solid (eluant: hexane/ethyl acetate = 7:3) with a minor inseparable impurity. The oxirane **20c** was further purified by HPLC (eluant: ethyl acetate/dichloromethane = 3:97, HPLC retention time 23.2 min) to afford a pure sample of 1-methanesulfonyl-3-oxiranyl-2,3-dihydro-1*H*-indole [**20c**, 0.025 g, 9% yield based on the amount of the starting arenediazonium salt **16** for entry (iii) of Table 1] as a colourless semi-solid. The oxirane **20c** was identified to be a 9:25 mixture of diastereoisomers by ^1^H NMR spectroscopic analysis. Its structure was further confirmed by 2D [1H, 1H] COSY NMR and ^13^C DEPT NMR spectral analysis.

**3-Ethyl-1-methanesulfonyl-2,3-dihydro-1*H*-indole (20b)** [5,6]: [Found: M^+•^ (EI), 225.0826. C_11_H_15_NO_2_S requires 225.0818]; IR (CH_2_Cl_2_) 3029, 2965, 2933, 2877, 1601, 1480, 1459, 1347, 1230, 1159, 1108, 1053, 1028, 998, 960, 747 cm^−1^; ^1^H NMR (400.13 MHz, CDCl_3_) *δ* 1.07 (t, *J* = 7.4 Hz, 3H), 1.62–1.74 (m, 1H), 1.87–1.99 (m, 1H), 2.93 (s, 3H), 3.30–3.43 (m, 1H), 3.70 (dd, *J* = 10.2, 6.5 Hz, 1H), 4.14 (dd, *J* = 10.2, 9.2, 1H), 7.11 (ddd, *J* = 7.5, 7.5, 0.9 Hz, 1H), 7.22–7.31 (m, 2H), 7.46 (dd, *J* = 7.8, 0.9 Hz, 1H); ^13^C NMR (100.61 MHz, CDCl_3_) *δ* 11.5 (CH_3_), 27.8 (CH_2_), 34.6 (CH_3_), 41.7 (CH), 56.1 (CH_2_), 113.7 (CH), 123.8 (CH), 124.9 (CH), 128.4 (CH), 135.2 (C), 142.0 (C); GC–MS retention time 16.01 min, *m/z* (EI) 225 (M^+•^, 57%), 196 (48), 146 (40), 130 (42), 118 (100), 103 (5), 91 (21), 89 (19), 77 (10).

**1-Methanesulfonyl-3-oxiranyl-2,3-dihydro-1*H*-indole (20c)** [mixture of diastereoisomers (9:25)]: [Found: MNH_4_^+^ (ESI), 257.0952. C_11_H_13_NO_3_S requires *MNH_4_*, 257.0954]; IR (neat film, NaCl) 3009, 2930, 1601, 1480, 1460, 1347, 1245, 1160, 1106, 1053, 1028, 987, 960, 861, 772 cm^−1^; ^1^H NMR (400.13 MHz, CDCl_3_) *δ* 2.60 (major) and 2.69 (minor) (2 × dd, *J* = 4.6, 2.6 and 4.6, 2.6 Hz, 1H), 2.82 (minor) and 2.87 (major) (2 × dd, *J* = 4.6, 3.9 and 4.6, 3.9 Hz, 1H), 2.92 (major) and 2.93 (minor) (2 × s, 3H), 3.13 (minor) and 3.18 (major) (2 × ddd, *J* = 6.4, 3.9, 2.6 and 5.6, 3.9, 2.6 Hz, 1H), 3.36 (minor) and 3.45 (major) (2 × ddd, *J* = 9.6, 6.4, 5.2 and 9.5, 5.6, 5.3 Hz, 1H), 3.90 (minor) and 3.93 (major) (2 × dd, *J* = 11.0, 5.2 and 10.8, 5.3 Hz, 1H), 4.05 (minor) and 4.09 (major) (2 × dd, *J* = 11.0, 9.6 and 10.8, 9.5 Hz, 1H), 7.05–7.14 (2 × m, major + minor, 1H), 7.20–7.33 (2 × m, major + minor, 2H), 7.38–7.48 (2 × m, major + minor, 1H); ^13^C NMR (100.61 MHz, CDCl_3_) *δ* (major + minor) 35.2 (CH_3_), 35.3 (CH_3_), 42.69 (CH), 42.73 (CH_3_), 45.7 (CH_2_), 45.8 (CH_2_), 52.4 (CH_2_), 53.0 (CH_2_), 53.7 (CH), 54.1 (CH), 114.3 (CH), 114.4 (CH), 124.3 (CH), 124.4 (CH), 126.0 (CH), 126.4 (CH), 129.7 (CH), 129.8 (CH), 130.4 (C), 131.5 (C), 142.5 (C), 142.8 (C); GC–MS retention time 16.24 (minor) and 16.27 (major) min, *m/z* (EI) 239 (M^+•^, 24%), 208 (5), 196 (14), 160 (4), 159 (4), 144 (3), 142 (9), 132 (11), 130 (100), 118 (31), 117 (33), 116 (18), 103 (23), 89 (28), 78 (59), 77 (47), 65 (11), 63 (29).

**Experimental Section II**

**(*Z*)-4-(*tert*-Butyldiphenylsilanyloxy)but-2-en-1-ol (24)**

*cis-*2-Butene-1,4-diol (**23**, 3.304 g, 37.5 mmol, 1.0 equiv) was added dropwise to a suspension of washed sodium hydride (1.8 g of 60% NaH dispersed in mineral oil, 45.0 mmol, 1.2 equiv) in freshly distilled THF (75 ml) at 0 °C under a nitrogen atmosphere and stirred for 0.5 h. To the suspension was added *tert*-butyldiphenylsilyl chloride (9.75 ml, 37.5 mmol, 1.0 equiv) dropwise at 0 °C. The reaction mixture was allowed to stir for 4 h at r.t. When the reaction was judged to be complete by TLC analysis, the solvent was evaporated to obtain an oily mixture which was extracted with ethyl acetate (150 ml) and washed with brine (3 × 100 ml) and deionised water (3 × 100 ml). The ethyl acetate layer was dried over anhydrous magnesium sulfate and evaporated to dryness to obtain a colourless oil that was purified by flash chromatography on silica [eluant: ethyl acetate/petroleum ether = 1:3] to give (*Z*)-4-(*tert*-butyldiphenylsilanyloxy)but-2-en-1-ol (**24**) [7,8] as a clear oil (10.357 g, 85%); IR (neat film, NaCl) 3307, 3071, 2931, 2858, 1589, 1472, 1428, 1112, 1078, 1029, 823, 702 cm^−1^; ^1^H NMR (400.13 MHz, CDCl_3_) *δ* 1.10 (s, 9H), 1.81 (s, 1H), 4.04 (d, *J* = 3.4 Hz, 2H), 4.30 (dd, *J* = 6.0, 1.1 Hz, 2H), 5.62–5.70 (dm, *J* = 11.2 Hz, 1H), 5.72–5.78 (dm, *J* = 11.2 Hz, 1H), 7.40–7.50 (m, 6H), 7.71–7.76 (m, 4H); ^13^C NMR (100.61 MHz, CDCl_3_) *δ* 19.6 (C), 27.3 (CH_3_), 59.2 (CH_2_), 60.8 (CH_2_), 128.2 (CH), 130.3 (CH), 130.5 (CH), 131.4 (CH), 134.0 (C), 136.1 (CH).

***N*-[(*Z*)-4-(*tert*-Butyldiphenylsilanyloxy)but-2-enyl]-*N*-(2-nitrophenyl)methanesulfonamide (26a)**

Triphenylphosphine (9.794 g, 37.34 mmol, 1.2 equiv) and *N*-(2-nitrophenyl)methanesulfonamide (**25a**, 6.728 g, 31.12 mmol, 1.0 equiv) were dissolved in freshly distilled tetrahydrofuran (200 ml) under a nitrogen atmosphere and cooled to 0 °C. To this solution was added (*Z*)-4-(*tert*-butyldiphenylsilanyloxy)but-2-en-1-ol (**24**, 10.160 g, 31.12 mmol, 1.0 equiv) followed by diisopropyl azodicarboxylate (7.40 ml, 37.34 mmol, 1.2 equiv). The reaction mixture was allowed to warm to room temperature and stirred under nitrogen for 12 h. The reaction mixture was then concentrated to dryness, dissolved in dichloromethane (250 ml) and washed sequentially with sodium hydroxide (2 M, 3 × 400 ml) and saturated brine solution (3 × 150 ml). The organic extracts were then dried over anhydrous magnesium sulfate followed by removal of the solvent *in* *vacuo*. Column chromatography on silica using toluene as the eluant gave the desired compound **26a** [7] as a white solid (12.985 g, 80%); mp 91–92 °C [Found: MNH_4_^+^ (ESI), 542.2139. C_27_H_32_N_2_O_5_SiS requires *MNH_4_*, 542.2145]; [Found: C, 61.74; H, 6.09; N, 5.44. C_27_H_32_N_2_O_5_SiS requires C, 61.81; H, 6.15; N, 5.34%]; IR (KBr disc) 3067, 3050, 2960, 2931, 2856, 1604, 1532, 1428, 1350, 1343, 1233, 1154, 1107, 1067, 998, 966, 892, 870 cm^−1^; ^1^H NMR (400.13 MHz, CDCl_3_) *δ* 1.02 (s, 9H), 2.97 (s, 3H), 4.03 (d, *J* = 5.8 Hz, 2H), 4.17 (broad s, 2H), 5.54–5.62 (dm, *J* = 11.1 Hz, 1H), 5.72–5.80 (dm, *J* = 11.1 Hz, 1H), 7.35–7.47 (m, 8H), 7.54 (ddd, *J* = 7.7, 7.7, 1.6 Hz, 1H), 7.61 (m, 4H), 7.84 (dd, *J* = 8.0, 1.6 Hz, 1H); ^13^C NMR (100.61 MHz, CDCl_3_) *δ* 19.6 (C), 27.3 (CH_3_), 40.8 (CH_3_), 48.9 (CH_2_), 60.2 (CH_2_), 124.8 (CH), 125.7 (CH), 128.2 (CH), 129.9 (CH), 130.3 (CH), 132.6 (C), 133.7 (C), 133.8 (CH), 134.0 (CH), 134.8 (CH), 136.0 (CH), 149.0 (C); *m/z* (CI) 542 [(M + NH_4_)^+^, 5%], 274 (31), 109 (98), 52 (100).

***N*-[(*Z*)-4-(*tert*-Butyldiphenylsilanyloxy)but-2-enyl]-*N*-(4-methoxy-2-nitrophenyl)methane- sulfonamide (26d)**

Following the experimental procedure described previously for the preparation of *N*-[(*Z*)-4-(*tert*-butyldiphenylsilanyloxy)but-2-enyl]-*N*-(2-nitrophenyl)methanesulfonamide (**26a**), *N*-(4-methoxy-2-nitrophenyl)methanesulfonamide (**25b**, 6.156 g, 25.0 mmol, 1.0 equiv), triphenylphosphine (7.868 g, 30.0 mmol, 1.2 equiv), (*Z*)-4-(*tert*-butyldiphenylsilanyloxy)but-2-en-1-ol (**24**, 8.163 g, 25.0 mmol, 1.0 equiv) and diisopropyl azodicarboxylate (5.95 ml, 30.0 mmol, 1.2 equiv) were allowed to react in dry THF (200 ml) for 12 h at r.t. to afford N-[(*Z*)-4-(*tert*-butyldiphenylsilanyloxy)-but-2-enyl]-*N*-(4-methoxy-2-nitrophenyl)methanesulfonamide (**26d**, 8.816 g, 64%) as a viscous yellow liquid (purification by flash chromatography on silica; eluant: toluene) [Found: MNH_4_^+^ (ESI), 572.2245. C_28_H_34_N_2_O_6_SiS requires *MNH_4_*, 572.2245]; IR (CHCl_3_) 3073, 3034, 2933, 2859, 1617, 1538, 1502, 1346, 1154, 1112, 1083, 964, 870, 824 cm^−1^; ^1^H NMR (400.13 MHz, CDCl_3_) *δ* 1.06 (s, 9H), 2.95 (s, 3H), 3.84 (s, 3H), 4.09 (broad s, 2H), 4.28 (broad s, 2H), 5.58–5.68 (dm, *J* = 11.0 Hz, 1H), 5.76–5.85 (dm, *J* = 11.0 Hz, 1H), 7.06 (dd, *J* = 8.9, 3.0 Hz, 1H), 7.31–7.51 (m, 8H), 7.61–7.69 (m, 4H); ^13^C NMR (100.61 MHz, CDCl_3_) *δ* 19.1 (C), 26.8 (CH_3_), 40.1 (CH_3_), 48.4 (CH_2_), 56.0 (CH_3_), 59.7 (CH_2_), 110.3 (CH), 119.0 (CH), 124.2 (C), 124.6 (CH), 127.7 (CH), 129.8 (CH), 133.3 (C), 134.1 (CH), 134.2 (CH), 135.5 (CH), 149.2 (C), 159.6 (C); *m/z* (CI) 572 [(M + NH_4_)^+^, 59%], 555 (7), 525 (51), 497 (10), 445 (20), 328 (33), 309 (40), 274 (93), 256 (17), 234 (18), 219 (32), 217 (87), 196 (100), 191 (52), 187 (18), 149 (32), 139 (53), 124 (19).

***N*-[(*Z*)-4-Hydroxybut-2-enyl]-*N*-(2-nitrophenyl)methanesulfonamide (27a)**

A solution of tetrabutylammonium fluoride (20.0 ml of 1 M solution in THF, 20.0 mmol, 2.9 equiv) was added to a solution of *N*-[(*Z*)-4-(*tert*-butyldiphenylsilanyloxy)-but-2-enyl]-*N*-(2-nitrophenyl)methanesulfonamide (**26a**, 3.600 g, 6.86 mmol, 1.0 equiv) in THF (15 ml) under a nitrogen atmosphere. The resulting mixture turned wine-red, and was stirred for 1.5 h at r.t. and evaporated to dryness under reduced pressure. The mixture was extracted with ethyl acetate (150 ml). The organic layer was washed with brine solution (3 × 100 ml) and deionised water (3 × 100 ml). The organic layer was dried over anhydrous Na_2_SO_4_, filtered and evaporated. Column chromatography on silica [eluant: dichloromethane/ethyl acetate = 1:1] gave *N*-[(*Z*)-4-hydroxybut-2-enyl]-*N*-(2-nitrophenyl)methanesulfonamide [7] (**27a**, 1.660 g, 85%) as a yellow solid; mp 56–58 °C [Found: MH^+^ (ESI), 287.0702. C_11_H_14_N_2_O_5_S requires *MH*, 287.0701]; (Found: C, 46.17; H, 5.01; N, 9.93. C_11_H_14_N_2_O_5_S requires C, 46.15; H, 4.93; N, 9.78%); IR (KBr disc) 3549, 3382, 3020, 2930, 2874, 1605, 1582, 1533, 1484, 1462, 1359, 1335, 1221, 1156, 1143, 1020, 970 cm^−1^; ^1^H NMR (400.13 MHz, CDCl_3_) *δ* 1.65 (s, 1H), 3.04 (s, 3H), 4.09 (d, *J* = 6.6 Hz, 2H), 4.36 (broad s, 2H), 5.66 (dt, *J* = 11.0, 7.5 Hz, 1H), 5.82 (dt, *J* = 11.0, 6.6 Hz, 1H), 7.52–7.57 (m, 2H), 7.66 (ddd, *J* = 7.8, 7.5, 1.5 Hz, 1H), 7.90–7.95 (m, 1H); ^13^C NMR (100.61 MHz, CDCl_3_) *δ* 41.1 (CH_3_), 48.8 (CH_2_), 58.4 (CH_2_), 125.8 (CH), 125.9 (CH), 130.2 (CH), 132.7 (C), 134.01 (CH), 134.03 (CH), 134.4 (CH), 149.0 (C); *m/z* (CI) 304 [(M + NH_4_)^+^, 100%], 255 (30), 237 (67), 175 (47), 159 (52), 131 (30), 109 (29).

***N*-[(*Z*)-4-Hydroxybut-2-enyl]-*N*-(4-methoxy-2-nitrophenyl)methanesulfonamide (27d)**

Following the experimental procedure described previously for the preparation of *N*-[(*Z*)-4-hydroxybut-2-enyl]-*N*-(2-nitrophenyl)methanesulfonamide (**27a**), *N*-[(*Z*)-4-(*tert*-butyldiphenylsilanyl­oxy)but-2-enyl]-*N*-(4-methoxy-2-nitrophenyl)methanesulfonamide (**26d**, 6.900 g, 12.44 mmol, 1.0 equiv) in THF (150 ml) was reacted with tetrabutylammonium fluoride (18.66 ml of 1 M solution in THF, 18.66 mmol, 1.5 equiv) at r.t. for 1.5 h to afford a dark brown semi-solid. This was purified by flash chromatography on silica (eluant: dichloromethane/ethyl acetate = 2:3) to obtain *N*-[(*Z*)-4-hydroxybut-2-enyl]-*N*-(4-methoxy-2-nitrophenyl)methanesulfonamide (**27d**, 3.828 g, 97%) as a pale yellow solid; mp 87.5–88.5 °C; [Found: MNH_4_^+^ (ESI), 334.1064. C_12_H_16_N_2_O_6_S requires *MNH_4_*, 334.1067]; IR (KBr disc) 3290, 3102, 2936, 2843, 1617, 1535, 1500, 1447, 1367, 1335, 1282, 1214, 1151, 1090, 1065, 1034,1011, 960, 917, 881, 856, 805, 759 cm^−1^; ^1^H NMR (400.13 MHz, CDCl_3_) *δ* 1.95 (s, 1H), 2.96 (s, 3H), 3.85 (s, 3H), 4.04 (broad s, 2H), 4.40 (broad s, 2H), 5.59–5.67 (dm, *J* = 11.0 Hz, 1H), 5.73–5.81 (dm, *J* = 11.0 Hz, 1H), 7.11 (dd, *J* = 8.9, 3.0 Hz, 1H), 7.37 (d, *J* = 3.0 Hz, 1H), 7.39 (d, *J* = 8.9 Hz, 1H); ^13^C NMR (100.61 MHz, CDCl_3_) *δ* 40.9 (CH_3_), 48.8 (CH_2_), 56.7 (CH_3_), 58.4 (CH_2_), 110.9 (CH), 119.7 (CH), 124.8 (C), 126.0 (CH), 134.3 (CH), 134.9 (CH), 149.6 (C), 160.3 (C); *m/z* (CI) 334 [(M + NH_4_)^+^, 100%], 317 (7), 299 (4), 287 (9), 264 (4), 217 (4), 205 (8), 191 (12), 189 (22), 161 (12), 149 (4), 139 (16), 124 (4).

***N*-[(*Z*)-4-Bromobut-2-enyl]-*N*-(2-nitrophenyl)methanesulfonamide (28a)**

***Method I (using PBr_3_ reagent)***

Phosphorus tribromide (0.39 ml, 4.19 mmol, 0.6 equiv) was added dropwise to a stirred solution of *N*-[(*Z*)-4-hydroxybut-2-enyl]-*N*-(2-nitrophenyl)methanesulfonamide (**27a**, 2.000 g, 6.99 mmol, 1.0 equiv) in dry THF (100 ml) at 0 °C under nitrogen. A drop of pyridine was added and the solution was stirred at r.t. for 1 h under a nitrogen atmosphere. The solution was cooled to 0 °C and methanol (2 ml) was added slowly to quench the reaction. The reaction mixture was evaporated to dryness under reduced pressure. The residue was dissolved in ethyl acetate (100 ml) and the organic layer was washed with saturated sodium bicarbonate solution (3 × 100 ml) and brine solution (1 × 150 ml). The organic layer was dried over anhydrous Na_2_SO_4_, filtered and taken to dryness to obtain *N*-[(*Z*)-4-bromobut-2-enyl]-*N*-(2-nitrophenyl)methanesulfonamide [7] (**28a**, 2.420 g, 99%) as a pale yellow solid; mp 94–95 °C [Found: MNH_4_^+^ (ESI), 366.0123. C_11_H_13_BrN_2_O_4_S requires *MNH_4_*, 366.0123]; IR (KBr disc) 3024, 2930, 1605, 1581, 1533, 1485, 1461, 1360, 1337, 1216, 1154, 1142, 1060, 960 cm^−1^; ^1^H NMR (400.13 MHz, CDCl_3_) *δ* 3.04 (s, 3H), 3.82 (d, *J* = 8.5, 2H), 4.41 (broad s, 2H), 5.71 (dt, *J* = 10.7, 7.5 Hz, 1H), 5.86–5.95 (dm, *J* = 10.7, 1H), 7.51–7.57 (m, 2H), 7.66 (ddd, *J* = 7.8, 7.7, 1.5 Hz, 1H), 7.93 (dd, *J* = 8.0, 1.5 Hz, 1H); ^13^C NMR (100.61 MHz, CDCl_3_) *δ* 25.1 (CH_2_), 40.8 (CH_3_), 47.7 (CH_2_), 125.7 (CH), 128.1 (CH), 130.0 (CH), 130.7 (CH), 132.1 (C), 133.8 (CH), 134.0 (CH), 148.8 (C); *m/z* (CI) 368 [{M(^81^Br)+NH_4_}^+^, 23%], 366 [{M(^79^Br)+NH_4_}^+^, 22%], 256 (21), 239 (100), 161 (65), 125 (39), 109 (38).

***Method II (NBS-DMS bromination method)***

Methyl sulfide (0.32 ml, 4.31 mmol, 1.0 equiv) was added dropwise to a solution of *N*-bromosuccinimide (0.768 g, 4.31 mmol, 1.0 equiv) in dry dichloromethane (5 ml) under a nitrogen atmosphere and stirred for 30 min at −30 °C to form a yellow complex. To this suspension was added a solution of *N*-[(*Z*)-4-hydroxybut-2-enyl]-*N*-(2-nitrophenyl)methanesulfonamide (**27a**, 1.235 g, 4.31 mmol, 1.0 equiv) in dry dichloromethane (10 ml) and the reaction mixture was slowly brought to room temperature and stirred under a nitrogen atmosphere for 3 h. When the reaction was judged to be complete by TLC analysis, dichloromethane (100 ml) was added to the reaction mixture and the contents were washed with brine solution (3 × 100 ml) and water (3 × 100 ml). The organic layer was separated, dried over anhydrous magnesium sulfate and evaporated to dryness under reduced pressure to obtain a yellow residue which was purified by flash chromatography on silica (eluant: ethyl acetate/petroleum ether = 1:4) to obtain *N*-[(*Z*)-4-bromobut-2-enyl]-*N*-(2-nitrophenyl)­methane­sulfonamide [7] (**28a**, 0.771 g, 51%) as a pale yellow solid; mp 94–95°C. The spectral data of the sulfonamide **28a** obtained from this method were identical with those of the same compound prepared from the previous method.

***N*-[(*Z*)-4-Bromobut-2-enyl]-*N*-(4-methoxy-2-nitrophenyl)methanesulfonamide (28d)**

Following the experimental procedure described for the preparation of *N*-[(*Z*)-4-bromobut-2-enyl]-*N*-(2-nitrophenyl)methanesulfonamide (**28a**, method I), *N*-[(*Z*)-4-hydroxybut-2-enyl]-*N*-(4-methoxy-2-nitrophenyl)methanesulfonamide (**27d**, 0.089 g, 0.25 mmol, 1.0 equiv) was reacted with phosphorus tribromide (0.035 ml, 0.375 mmol, 1.5 equiv) in dry dichloromethane (20 ml) in the presence of pyridine (1 drop) at r.t. for 1 h to afford a yellow sticky semi-solid. This was purified by flash chromatography on silica gel (eluant: ethyl acetate/petroleum ether = 2:3) to obtain *N*-[(*Z*)-4-bromobut-2-enyl]-*N*-(4-methoxy-2-nitrophenyl)methanesulfonamide (**28d**, 0.088 g, 93%) as a pale yellow solid; mp 84.5–85.5 °C [Found: M^+•^ (EI), 377.9887. C_12_H_15_N_2_O_5_S^79^Br requires *M*, 377.9880]; IR (KBr disc) 3096, 3017, 2933, 2844, 1616, 1572, 1538, 1505, 1463, 1443, 1343, 1301, 1277, 1220, 1151, 1092, 1058, 1034, 961, 917, 859, 806 cm^−1^; ^1^H NMR (400.13 MHz, CDCl_3_) *δ* 2.98 (s, 3H), 3.83 (d, *J* = 8.4 Hz, 2H), 3.88 (s, 3H), 4.37 (broad s, 2H), 5.72 (dt, *J* = 10.6, 7.6 Hz, 1H), 5.89 (dt, *J* = 10.6, 8.4 Hz, 1H), 7.14 (dd, *J* = 8.8, 3.0 Hz, 1H), 7.37–7.42 (m, 2H); ^13^C NMR (100.61 MHz, CDCl_3_) *δ* 25.3 (CH_2_), 40.5 (CH_3_), 47.7 (CH_2_), 56.4 (CH_3_), 110.7 (CH), 119.4 (CH), 124.2 (C), 128.3 (CH), 130.5 (CH), 134.8 (CH), 149.4 (C), 160.1 (C); *m/z* (EI) 380 [{M (^81^Br)}^+•^, 100%], 378 [{M (^79^Br)}^+•^, 99%], 363 (51), 361 (50).

***N*-(2-Nitrophenyl)-*N*-[(*Z*)-4-phenylsulfanylbut-2-enyl]methanesulfonamide (29a)**

Sodium hydride (0.625 g of 60% NaH dispersed in mineral oil, 15.64 mmol, 1.20 equiv) was washed with dry *n*-hexane (2 × 25 ml) and suspended in dry THF (50 ml) under a nitrogen atmosphere. Thiophenol (1.47 ml, 14.33 mmol, 1.1 equiv) was added to this suspension at 0 °C under a nitrogen atmosphere. The resulting suspension was stirred for 10 min at 0 °C and another 50 min at r.t. To this creamy suspension was added a solution of *N*-[(*Z*)-4-bromobut-2-enyl]-*N*-(2-nitrophenyl)methanesulfonamide (**28a**, 4.550 g, 13.03 mmol, 1.0 equiv) in THF (50 ml) at 0 °C using a cannula. The reaction mixture was slowly brought to r.t. and stirred under a nitrogen atmosphere for 5 h. The reaction mixture and evaporated to dryness under reduced pressure and the resulting residue was extracted with ethyl acetate (150 ml). The organic layer was sequentially washed with a saturated solution of sodium bicarbonate (3 × 200 ml), brine (2 × 200 ml), dried over anhydrous Na_2_SO_4_, filtered and evaporated to dryness. Purification by flash chromatography on silica [eluant: ethyl acetate/petroleum ether = 1:4] to yield *N*-(2-nitrophenyl)-*N*-[(*Z*)-4-phenylsulfanylbut-2-enyl]methanesulfonamide [7] (**29a**, 4.831 g, 98%) as a highly viscous yellow oil which later crystallised into a low-melting pale yellow solid on prolonged storage at −23 °C; mp 39–40.5 °C [Found: MNH_4_^+^ (ESI), 396.1051. C_17_H_18_N_2_O_4_S_2_ requires *MNH_4_*, 396.1052]; (Found: C, 54.17; H, 4.82; N, 7.67. C_17_H_18_N_2_O_4_S_2_ requires C, 53.95; H, 4.79; N, 7.40%); IR (CHCl_3_) 3020, 2935, 1604, 1535, 1348, 1216, 1154 cm^−1^; ^1^H NMR (400.13 MHz, CDCl_3_) *δ* 2.98 (s, 3H), 3.37 (d, *J* = 7.7 Hz, 2H), 4.12 (broad s, 2H), 5.61 (dt, *J* = 10.8, 7.3 Hz, 1H), 5.70 (dt, *J* = 10.8, 7.7 Hz, 1H), 7.20–7.32 (m, 5H), 7.45 (dd, *J* = 7.9, 1.3 Hz, 1H), 7.51 (ddd, *J* = 8.0, 7.7, 1.3 Hz, 1H), 7.62 (ddd, *J* = 7.9, 7.7, 1.6 Hz, 1H), 7.89 (dd, *J* = 8.0, 1.6 Hz, 1H); ^13^C NMR (100.61 MHz, CDCl_3_) *δ* 31.7 (CH_2_), 41.0 (CH_3_), 48.2 (CH_2_), 125.8 (CH), 126.8 (CH), 127.5 (CH), 129.5 (CH), 130.1 (CH), 130.7 (CH), 131.6 (CH), 132.5 (C), 133.9 (CH), 134.2 (CH), 135.6 (C), 149.1 (C); *m/z* (CI) 396 [(M+NH_4_)^+^, 100%], 349 (30), 269 (16), 256 (28), 239 (75), 161 (48), 109 (25).

***N*-[(*Z*)-4-Benzenesulfinylbut-2-enyl]-*N*-(2-nitrophenyl)methanesulfonamide (29b)**

Sodium periodate (4.278 g, 20.0 mmol, 5.0 equiv) was added to a solution of *N*-(2-nitrophenyl)-*N*-[(*Z*)-4-phenylsulfanylbut-2-enyl]methanesulfonamide (**29a**, 1.514 g, 4.0 mmol, 1.0 equiv) in a 1:1 mixture of water (40 ml) and methanol (40 ml). This mixture was stirred for 75 min at r.t. under a nitrogen atmosphere. When the reaction was complete, much of the solvent was removed under reduced pressure, and the resulting mixture was poured into a separating funnel containing water (50 ml) and was extracted with ethyl acetate (4 × 50 ml). The combined organic phases were dried over anhydrous magnesium sulfate, filtered and evaporated to dryness under reduced pressure to afford a highly viscous yellow oil. This was purified using flash chromatography on silica [eluant: ethyl acetate/petroleum ether = 4:1] to afford *N*-[(*Z*)-4-benzenesulfinyl-but-2-enyl]-*N*-(2-nitrophenyl)­methanesulfonamide (**29b**) [7] as a sticky yellow semi-solid (1.448 g, 92%). The product was obtained as a 1:1 mixture of diastereoisomers (for more explanation see below) as observed in both ^1^H and ^13^C NMR spectra [Found: MH^+^ (ESI), 395.0736. C_17_H_18_N_2_O_5_S_2_ requires *MH*, 395.0735]; IR (neat film, NaCl) 3061, 3034, 2929, 2882, 1603, 1581, 1538, 1532, 1485, 1444, 1339, 1227, 1153, 1088, 1070, 1042, 962 cm^−1^; ^1^H NMR (400.13 MHz, CDCl_3_) *δ* 2.98 and 3.03 (2 × s, 3H), 3.35–3.43 (2 × m, 1H), 3.46–3.61 (2 × m, 1H), 4.10 and 4.22 (2 × broad s, 2H), 5.35–5.56 (2 × m, 1H), 5.75–5.96 (2 × m, 1H), 7.44–7.58 (2 × m, 7H), 7.58–7.70 (2 × m, 1H), 7.86–7.96 (2 × m, 1H); ^13^C NMR (100.61 MHz, CDCl_3_) *δ* (signals for 2 diastereoisomers) 40.7 (CH_3_), 41.0 (CH_3_), 48.5 (CH_2_), 53.9 (CH_2_), 54.7 (CH_2_), 59.6 (CH_2_), 121.8 (CH), 123.6 (CH), 124.6 (CH), 124.7 (CH), 125.9 (CH), 129.67 (CH), 129.69 (CH), 130.2 (CH), 131.7 (CH), 131.8 (CH), 132.45 (C), 132.48 (C), 132.7 (CH), 133.6 (CH), 134.0 (CH), 134.1 (CH), 143.2 (C), 149.1 (C); *m/z* (CI) 395 [(M+H)^+^, 2%], 190 (53), 124(92), 77 (100), 51 (73).

**Explanation for observation of diastereoisomers in sulfonamide 29b:**

The ^1^H NMR spectrum of sulfonamide **29b** (for ^1^H and ^13^C NMR spectroscopic data of **29b** see above) showed two closely related sets of signals indicating that it could be a 1:1 mixture of stereoisomers. Similarly two close set of signals were observed in its ^13^C NMR spectrum. The possibility of the presence of two geometrical isomers for **29b** was ruled out from the fact it was obtained from its precursor sulfide **29a,** which was predominantly a *Z*-isomer. The sulfone **29c** was isolated predominantly as a *Z*-isomer, by oxidation of the sulfoxide **29b**. Hence, it was concluded that the sulfoxide **29b** was isolated as a mixture of 1:1 diastereoisomers (Scheme 11).

## Scheme 11: Diastereoisomers [29b(I)–(II) and 29b(III)–(IV)] of the sulfonamide 29b.

The sulfoxide is chiral (the diastereotopic protons of the CH_2_SO group were distinctly observed at *δ* 2.98 and 3.03 in ^1^H NMR spectrum), and the molecule **29b** also exhibited an axial chirality due to restricted rotation of the 2-nitrophenyl group about the N-C(Ar) bond.

***N*-[(*Z*)-4-Benzenesulfonylbut-2-enyl]-*N*-(2-nitrophenyl)methanesulfonamide (29c)**

*N*-(2-Nitrophenyl)-*N*-[(*Z*)-4-phenylsulfanylbut-2-enyl]methanesulfonamide (**29a**, 1.514 g, 4.0 mmol, 1.0 equiv) was dissolved in a 1:1 mixture of methanol (40 ml) and water (40 ml) and sodium periodate (4.278 g, 20.0 mmol, 5.0 equiv) was added. The reaction mixture was stirred under a nitrogen atmosphere at r.t. for 72 h. When the reaction was complete, much of the solvent was removed under reduced pressure, and the resulting mixture was poured into a separating funnel containing water (50 ml) and was extracted with ethyl acetate (4 × 50 ml). The combined organic phases were dried over anhydrous magnesium sulfate, filtered and evaporated to dryness under reduced pressure to afford a highly viscous yellow oil. This was purified using flash chromatography on silica [eluant: ethyl acetate/petroleum ether = 1:1] to afford *N*-[(*Z*)-4-benzenesulfonylbut-2-enyl]-*N*-(2-nitrophenyl)methanesulfonamide (**29c**, 1.349 g, 82%) as a sticky yellow semi-solid [Found: MNH_4_^+^ (ESI), 428.0953. C_17_H_18_N_2_O_6_S_2_ requires *MNH_4_*, 428.0945]; IR (neat film, NaCl) 3028, 2933, 1604, 1583, 1533, 1485, 1448, 1345, 1307, 1144, 1085, 961, 907, 845 cm^−1^; ^1^H NMR (400.13 MHz, CDCl_3_) *δ* 2.94 (s, 3H), 3.80 (d, *J* = 8.0 Hz, 2H), 4.10 (broad s, 2H), 5.59 (dt, *J* = 10.8, 8.0 Hz, 1H), 5.95 (dt, *J* = 10.8, 7.3 Hz, 1H), 7.46 (dd, *J* = 7.9, 1.3 Hz, 1H), 7.50–7.59 (m, 3H), 7.60–7.69 (m, 2H), 7.84 (m, 2H), 7.89 (dd, *J* = 8.0, 1.5 Hz, 1H); ^13^C NMR (100.61 MHz, CDCl_3_) *δ* 40.7 (CH_3_), 48.4 (CH_2_), 55.1 (CH_2_), 120.7 (CH), 126.0 (CH), 128.8 (CH), 129.8 (CH), 130.3 (CH), 132.4 (C), 133.5 (CH), 133.6 (CH), 134.1 (CH), 134.5 (CH), 138.9 (C), 149.1 (C); *m/z* (CI) 428 [(M+NH_4_)^+^, 31%], 410 (4), 396 (4), 381 (9), 304 (6), 288 (8), 256 (8), 241 (37), 212 (100), 187 (23), 163 (48), 159 (72), 148 (31), 126 (68), 109 (98), 94 (68), 83 (42), 72 (19).

***N*-(4-Methoxy-2-nitrophenyl)-*N*-[(*Z*)-4-phenylsulfanylbut-2-enyl]methanesulfonamide (29d)**

Following the experimental procedure described previously for the preparation of *N*-(2-nitrophenyl)-*N*-[(*Z*)-4-phenylsulfanylbut-2-enyl]methanesulfonamide (**29a**), thiophenol (0.57 ml, 5.5 mmol, 1.1 equiv) was reacted with *N*-[(*Z*)-4-bromo-but-2-enyl]-*N*-(4-methoxy-2-nitrophenyl)methanesulfonamide (**28d**, 1.896 g, 5.0 mmol, 1.0 equiv) using sodium hydride (0.240 g of 60% NaH dispersed in mineral oil, 6.0 mmol, 1.2 equiv) as the base to afford *N*-(4-methoxy-2-nitrophenyl)-*N*-[(*Z*)-4-phenylsulfanylbut-2-enyl]methanesulfonamide (**29d**, 1.922 g, 94%) as a pale yellow solid [eluant: ethyl acetate/petroleum ether = 1:4]; mp 97.5–98.5 °C [Found: MNH_4_^+^ (ESI), 426.1166. C_18_H_20_N_2_O_5_S_2_ requires *MNH_4_*, 426.1152]; IR (KBr disc) 3077, 3012, 2984, 2940, 2896, 1614, 1583, 1548, 1493, 1438, 1341, 1299, 1267, 1259, 1229, 1149, 1093, 1058, 1031, 968, 955, 913 cm^−1^; ^1^H NMR (400.13 MHz, CDCl_3_) *δ* 2.94 (s, 3H), 3.38 (d, *J* = 7.7 Hz, 2H), 3.87 (s, 3H), 4.19 (broad s, 2H), 5.61 (dt, *J* = 10.8, 7.3 Hz, 1H), 5.71 (dt, *J* = 10.8, 7.7 Hz, 1H), 7.10 (dd, *J* = 8.9, 3.0 Hz, ArH), 7.17–7.32 (m, 5H), 7.34 (d, *J* = 8.9 Hz, 1H), 7.38 (d, *J* = 3.0 Hz, 1H); ^13^C NMR (100.61 MHz, CDCl_3_) *δ* 31.3 (CH_2_), 40.6 (CH_3_), 47.9 (CH_2_), 56.3 (CH_3_), 110.5 (CH), 119.3 (CH), 124.3 (C), 126.7 (CH), 127.1 (CH), 129.2 (CH), 130.3 (CH), 131.2 (CH), 135.0 (CH), 135.4 (C), 149.3 (C), 160.0 (C); *m/z* (CI) 426 [(M+NH_4_)^+^, 30%], 379 (8), 269 (45), 191 (71), 176 (20), 163 (100), 149 (38), 139 (70), 124 (76), 113 (19).

***N*-(2-Aminophenyl)-*N*-[(*Z*)-4-phenylsulfanylbut-2-enyl]methanesulfonamide (30a)**

Under nitrogen, copper(II) acetylacetonate (1.047 g, 4.0 mmol, 2.00 equiv) was added to ethanol (75 ml). To the suspension, sodium borohydride (0.151 g, 4.0 mmol, 2.00 equiv) was flushed in using ethanol (50 ml). The mixture was stirred at room temperature for 30 min, until the catalyst was ready (black granular solid in a clear solution) and hydrogen evolution had decreased. To this, a solution of *N*-(2-nitrophenyl)-*N*-[(*Z*)-4-phenylsulfanylbut-2-enyl]methanesulfonamide (**29a**, 0.757 g, 2.0 mmol, 1.0 equiv) in ethanol (75 ml) was added, followed by further sodium borohydride (0.151 g, 4.0 mmol, 2.0 equiv), again flushed in with ethanol (50 ml). The reaction was stirred overnight for 15 h and quenched with water (50 ml). The reaction mixture was filtered through Celite® 521, and the filtrate was evaporated to dryness under reduced pressure. The resulting residue was partitioned between water (150 ml) and diethyl ether (100 ml). The layers were separated and the aqueous layer was extracted with more diethyl ether (50 ml). The combined organic layers were washed sequentially with ammonia solution (2 M, 3 × 100 ml), brine solution (3 × 100 ml), deionised water (3 × 100 ml), dried over anhydrous sodium sulfate, filtered and evaporated to dryness to obtain a brown oil. This was purified by column chromatography [eluant: ethyl acetate/dichloromethane = 1:19] on silica to obtain *N*-(2-aminophenyl)-*N*-[(*Z*)-4-phenylsulfanylbut-2-enyl]methanesulfonamide [7] (**30a**, 0.486 g, 70%) as a brown-yellow oil [Found: MH^+^ (ESI), 349.1035. C_17_H_20_N_2_O_2_S_2_ requires *MH*, 349.1044]; IR (neat film, NaCl) 3476, 3380, 3055, 3029, 2930, 1617, 1583, 1500, 1481, 1458, 1439, 1332, 1210, 1149, 1088, 1053, 958 cm^−1^; ^1^H NMR (400.13 MHz, CDCl_3_) *δ* 3.00 (s, 3H), 3.47 (d, *J* = 7.5 Hz, 2H), 3.99 (broad s, 2H), 4.13 (d, *J* = 6.6 Hz, 2H), 5.58–5.68 (dm, *J* = 10.6 Hz, 1H), 5.68–5.78 (dm, *J* = 10.6 Hz, 1H), 6.74–6.80 (m, 1H), 6.82 (d, *J* = 8.0 Hz, 1H), 7.11 (dd, *J* = 7.9, 0.9 Hz, 1H), 7.15–7.22 (m, 1H), 7.23–7.38 (m, 5H); ^13^C NMR (100.61 MHz, CDCl_3_) *δ* 31.6 (CH_2_), 38.7 (CH_3_), 47.6 (CH_2_), 117.5 (CH), 119.0 (CH), 124.9 (C), 127.0 (CH), 127.2 (CH), 129.0 (CH), 129.4 (CH), 130.2 (CH), 130.4 (CH), 131.2 (CH), 136.0 (C), 147.0 (C); *m/z* (CI) 349 [(M+H)^+^, 85%], 241 (100), 187 (54), 119 (27), 109 (46), 52 (13).

***N*-(2-Amino-4-methoxyphenyl)-*N*-[(*Z*)-4-phenylsulfanylbut-2-enyl]methanesulfonamide (30d)**

*N*-(4-Methoxy-2-nitrophenyl)-*N*-[(*Z*)-4-phenylsulfanylbut-2-enyl]methanesulfonamide (**29d**, 0.6127 g, 1.5 mmol, 1.0 equiv) was dissolved in dichloromethane (2 ml) and methanol (25 ml) and stannous chloride dihydrate (1.6922 g, 7.5 mmol, 5.0 equiv) was added to the solution at room temperature. The reaction mixture was heated to reflux at 65 °C under a nitrogen atmosphere for 4 h. When the reaction was complete, much of the solvent was evaporated under reduced pressure and the pH of the reaction mixture was adjusted to 8.0 by addition of saturated sodium bicarbonate solution and the solution was extracted with ethyl acetate (3 × 50 ml). The combined organic phases were washed with brine solution (5 × 100 ml), separated, dried over anhydrous sodium sulfate, filtered and evaporated to dryness to obtain a yellow viscous oil. This was purified by flash chromatography on silica gel [eluant: ethyl acetate/petroleum ether = 2:3] to afford *N*-(2-amino-4-methoxyphenyl)-*N*-[(*Z*)-4-phenylsulfanylbut-2-enyl]methanesulfonamide (**30d** (0.517 g, 91%) as a pale yellow sticky semi-solid [Found: MH^+^ (ESI), 379.1140. C_18_H_22_N_2_O_3_S_2_ requires *MH*, 379.1145]; IR (neat film, NaCl) 3478, 3379, 3056, 3020, 2933, 2837, 1621, 1584, 1509, 1469, 1481, 1452, 1439, 1331, 1215, 1172, 1149, 1088, 1056, 1027, 958 cm^−1^; ^1^H NMR (400.13 MHz, CDCl_3_) *δ* 2.92 (s, 3H), 3.44 (dd, *J* = 7.2, 3.7 Hz, 2H), 3.74 (s, 3H), 3.99–4.15 (broad m, 4H), 5.58 (dt, *J* = 10.7, 7.2 Hz, 1H), 5.68 (dt, *J* = 10.7, 7.6 Hz, 1H), 6.26–6.31 (m, 2H), 6.97 (dd, *J* = 6.7, 2.4 Hz, 1H), 7.18–7.23 (m, 1H), 7.26–7.34 (m, 4H); ^13^C NMR (100.61 MHz, CDCl_3_) *δ* 31.1 (CH_2_), 38.1 (CH_3_), 47.2 (CH_2_), 55.4 (CH_3_), 101.5 (CH), 104.8 (CH), 117.5 (C), 126.69 (CH), 126.74 (CH), 129.0 (CH), 129.5 (CH), 129.7 (CH), 130.6 (CH), 135.6 (C), 147.6 (C), 160.7 (C); *m/z* (CI) 379 [(M+H)^+^, 100%], 300 (12), 271 (81), 234 (12), 217 (58), 191 (50), 176 (15), 163 (33), 149 (31), 139 (93), 124 (59), 113 (18), 110 (11), 98 (6), 84 (6).

***N*-(2-Aminophenyl)-*N*-[(*Z*)-4-benzenesulfinyl-but-2-enyl]methanesulfonamide (30b)**

Following the experimental procedure described previously for the preparation of *N*-(2-amino-4-methoxyphenyl)-*N*-[(*Z*)-4-phenylsulfanylbut-2-enyl]methanesulfonamide (**30d**), *N*-[(*Z*)-4-benzenesulfinyl but-2-enyl]-*N*-(2-nitrophenyl)methanesulfonamide (**29b**, 0.493 g, 1.25 mmol, 1.0 equiv) was reacted with stannous chloride dihydrate (1.410 g, 6.25 mmol, 5.0 equiv) in methanol (20 ml) at 65 °C for 4 h under a nitrogen atmosphere to afford *N*-(2-aminophenyl)-*N*-[(*Z*)-4-benzenesulfinylbut-2-enyl]methanesulfonamide [7] (**30b**, 0.325 g, 71%) as a yellow semi-solid [eluant: ethyl acetate/dichloromethane = 11:9] which later crystallised to a yellow solid on storage. The product was obtained as a 1:1 mixture of diastereoisomers as observed in both ^1^H and ^13^C NMR spectra; mp 135.5–136.5 °C; [Found: MH^+^ (ESI), 365.0998. C_17_H_20_N_2_O_3_S_2_ requires *MH*, 365.0993]; IR (KBr disc) 3466, 3375, 3058, 3013, 2928, 1620, 1500, 1458, 1444, 1332, 1213, 1148, 1087, 1039, 964 cm^−1^; ^1^H NMR (400.13 MHz, CDCl_3_) *δ* 2.95 and 2.99 (2 × s, 3H), 3.35–3.60 (2 × m, 2H), 3.85–4.28 (broad m, 4H), 5.34–5.45 (2 × m, 1H), 5.70–5.91 (2 × m, 1H), 6.69–6.81 (m, 2H), 7.05–7.17 (m, 2H), 7.44–7.55 (m, 5H); ^13^C NMR (100.61 MHz, CDCl_3_) *δ* (signals for 2 diastereoisomers) 38.5 (CH_3_), 38.7 (CH_3_), 47.6 (CH_2_), 52.9 (CH_2_), 54.6 (CH_2_), 64.2 (CH_2_), 117.2 (CH), 117.3 (CH), 118.7 (CH), 120.9 (CH), 122.1 (CH), 124.42 (CH), 124.45 (CH), 124.7 (2 × C), 128.87 (CH), 128.91 (CH), 129.30 (CH), 129.33 (CH), 129.4 (CH), 130.1 (CH), 130.2 (CH), 131.3 (CH), 131.4 (CH), 132.6 (CH), 134.1 (CH), 142.9 (2 × C), 146.7 (2 × C); *m/z* (CI) 365 [(M+H)^+^, 43%], 349 (43), 257 (56), 241 (87), 204 (23), 187 (100), 163 (29), 119 (21), 109 (35), 52 (21). For explanation regarding the observation of diastereoisomerism in sulfonamide **30b**, see a similar explanation in Scheme 11 for the sulfonamide **29b**.

***N*-(2-Aminophenyl)-*N*-[(*Z*)-4-benzenesulfonyl-but-2-enyl]methanesulfonamide (30c)**

Following the experimental procedure described for the preparation of *N*-(2-amino-4-methoxyphenyl)-*N*-[(*Z*)-4-phenylsulfanylbut-2-enyl]methanesulfonamide (**30d**), a solution of *N*-[(*Z*)-4-benzenesulfonylbut-2-enyl]-*N*-(2-nitrophenyl)methanesulfonamide (**29c**, 1.1904 g, 2.9 mmol, 1.0 equiv) dissolved in dichloromethane (2 ml) and methanol (25 ml) was reacted with stannous chloride dihydrate (3.2716 g, 14.5 mmol, 5.0 equiv) at 65 °C for 3.5 h to afford *N*-(2-aminophenyl)-*N*-[(*Z*)-4-benzenesulfonyl-but-2-enyl]methanesulfonamide (**30c**, 0.916 g, 83%) as a sticky yellow foam. [Found: MH^+^ (ESI), 381.0938. C_17_H_20_N_2_O_4_S_2_ requires *MH*, 381.0937]; IR (neat film, NaCl) 3479, 3380, 3063, 3029, 2931, 1621, 1585, 1501, 1458, 1447, 1413, 1331, 1307, 1239, 1213, 1147, 1085, 1054, 960, 904, 825 cm^−1^; ^1^H NMR (400.13 MHz, CDCl_3_) *δ* 2.95 (s, 3H), 3.81 (d, *J* = 8.0 Hz, 2H), 4.08 (broad s, 2H), 5.56 (dt, *J* = 10.7, 8.0 Hz, 1H), 5.90 (dt, *J* = 10.7, 7.3 Hz, 1H), 6.70–6.85 (m, 2H), 7.03–7.11 (m, 1H), 7.16 (ddd, *J* = 8.3, 8.2, 1.1 Hz, 1H), 7.54–7.59 (m, 2H), 7.64–7.70 (m, 1H), 7.85–7.90 (m, 2H); ^13^C NMR (100.61 MHz, CDCl_3_) *δ* 38.7 (CH_3_), 47.7 (CH_2_), 55.3 (CH_2_), 117.6 (CH), 119.1 (CH), 120.2 (CH), 124.7 (C), 128.9 (CH), 129.1 (CH), 129.8 (CH), 130.6 (CH), 133.8 (CH), 134.4 (CH), 139.0 (C), 146.9 (C); *m/z* (CI) 381 [(M+H)^+^, 14%], 380 (1), 303 (3), 241 (48), 212 (100), 204 (10), 187 (34), 161 (30), 159 (14), 146 (8), 126 (29), 109 (71), 96 (14), 94 (27), 84 (6).

**2-[Methanesulfonyl-{(*Z*)-4-phenylsulfanyl-but-2-enyl}amino]benzenediazonium tetrafluoroborate (31a)**

A solution of *N*-(2-aminophenyl)-*N*-[(*Z*)-4-phenylsulfanylbut-2-enyl]methanesulfonamide (**30a**, 0.390 g, 1.12 mmol, 1.00 equiv) in dry dichloromethane (10 ml) was added to a stirred suspension of nitrosonium tetrafluoroborate (0.196 g, 1.68 mmol, 1.50 equiv) in dry dichloromethane (10 ml) at −20 °C. The reaction mixture was stirred vigorously for 1.5 h under a nitrogen atmosphere between −20 to −10 °C. After 1.5 h, when the reaction was judged to be complete by TLC analysis, the dichloromethane was evaporated under reduced pressure. The yellow residue thus obtained was dissolved in a little acetone (1 ml, reagent grade) and recrystallised from cold dry diethyl ether between −10 °C and 0 °C, then filtered under nitrogen to give the arenediazonium salt **31a** [7] (0.491 g, 98%) as fine yellow needles which started to decompose into a yellow semi-solid when brought to r.t. and in contact with air; ^1^H NMR (400.13 MHz, acetone-*d*_6_) *δ* 3.23 (s, 3H), 3.68 (d, *J* = 7.1 Hz, 2H), 4.62 (d, *J* = 6.1 Hz, 2H), 5.60–5.72 (m, 2H), 7.17–7.23 (m, 1H), 7.26–7.37 (m, 3H), 7.50–7.60 (m, 1H), 8.04 (ddd, *J* = 8.4, 7.7, 0.9 Hz, 1H), 8.32 (dd, *J* = 8.3, 0.9 Hz, 1H), 8.44 (ddd, *J* = 8.3, 7.7, 1.5 Hz, 1H), 8.90 (dd, *J* = 8.4, 1.5 Hz, 1H).

**Table 2:** Reductive radical cyclization of arenediazonium salts **31a–d** by TDAE.

| **Entry** | **Diazonium**  **Salt** | **General**  **Procedure** | **Equivalents**  **of TDAE** | **Solvent** | **Temperature**  **(°C)** | **Time** | **Isolated Yield (%) of** | | |
| --- | --- | --- | --- | --- | --- | --- | --- | --- | --- |
|  |  |  |  |  |  |  | **20a/32** | **33** | **21** |
| (i) | **31a** | **A** | 1.0 | Acetonitrile | 25 | 5 min | 88 | 82 | 12 |
| (ii)^a^ | **31a** | A | 0 | Acetonitrile | 25 | 5 min | 0 | 0 | 0 |
| (iii)^b,d^ | **31a** | **C** | 3.0 | Acetonitrile | 25 | 5 min | 85 | 39 | –^c^ |
| (iv) | **31a** | A | 1.0 | Acetone | 25 | 5 min | 84 | 78 | 17 |
| (v) | **31a** | A | 1.0 | Methanol | 0 to 25 | 2 h | 81 | 34 | 71 |
| (vi)^d^ | **31b** | D | 1.0 | Acetone | 25 | 5 min | 81 | 0 | 24 |
| (vii)^d^ | **31c** | D | 1.0 | Acetone | 25 | 5 min | 63 | 0 | 41 |
| (viii)^d^ | **31d** | D | 1.0 | Acetone | 25 | 5 min | 88 | 55 | 35 |

^a^Control reaction performed in the absence of TDAE reagent. ^b^This experiment was conducted by adding a solution of the diazonium salt in dry MeCN to a solution of TDAE in dry MeCN, while the other experiments in this table all featured addition of the TDAE to the diazonium salt. ^c^[TDAE]^++^ 2[BF_4_^─^] salt **21** was not isolated from the reaction. ^d^In these experiments, the arenediazonium salts **31a–d** were made *in situ* from their corresponding amines **30a–d**, while all the other experiments in this table featured direct use of arenediazonium salt.

**Representative General Procedure A [Table 2, entry (i)]:** A 25 ml two-necked round bottomed flask equipped with a magnetic stirring bar and a septum was flame-dried *in vacuo*, backfilled with dry argon gas. This procedure was repeated at least thrice and the flask was loaded with freshly distilled dry acetonitrile (5 ml). The solvent was degassed by purging with a slow stream of argon gas for 30 min at r.t. and a pure sample of 2-[methanesulfonyl-{(*Z*)-4-phenylsulfanylbut-2-enyl}amino]benzene­diazonium tetrafluoroborate (**31a**, 0.100 g, 0.224 mmol, 1.0 equiv) was added to it, followed by addition of TDAE (0.05 ml, 0.224 mmol, 1.0 equiv) in one shot using a syringe [the sample of TDAE was handled inside a glove box under a nitrogen atmosphere prior to its use] under an argon atmosphere at 25 °C. The reaction mixture turned deep red in colour immediately with evolution of bubbles of nitrogen and gradually turned to orange with precipitation of off-white solid. The reaction mixture was stirred at 25 °C for 5 min under an argon atmosphere and the white precipitate was filtered, washed with ethyl acetate (3 × 25 ml) under argon and dried *in vacuo* to obtain the salt octamethyloxamidinium bis(tetrafluoroborate) (**21**, 0.005 g, 12%) as a white powder. The combined organic filtrate was washed with water (3 × 100 ml), separated and dried over anhydrous magnesium sulfate and evaporated to dryness under reduced pressure to obtain a yellow residue which was purified by flash chromatography on silica gel to obtain diphenyl disulfide (**33**) [7,10-12] (eluant: ethyl acetate/petroleum ether = 1:99) as a white waxy solid (0.02 g, 82%) and 1-methanesulfonyl-3-vinyl-2,3-dihydro-1*H*-indole [7] (**20a**, eluant: ethyl acetate/petroleum ether = 1:9) as a yellow solid (0.044 g, 88%).

**1-Methanesulfonyl-3-vinyl-2,3-dihydro-1*H-*indole (20a):** [7] mp 52–54 °C [Found: MNH_4_^+^ (ESI), 241.1011. C_11_H_13_NO_2_S requires *MNH_4_*, 241.1011]; IR (KBr disc) 3079, 3010, 2930, 1677, 1640, 1605, 1478, 1459, 1350, 1231, 1168, 1105, 1053 cm^−1^; ^1^H NMR (400.13 MHz, CDCl_3_) *δ* 2.89 (s, 3H), 3.68 (dd, *J* = 10.4, 7.8 Hz, 1H), 4.03 (ddd, *J* = 9.4, 7.9, 7.8 Hz, 1H), 4.21 (dd, *J* = 10.4, 9.4 Hz, 1H), 5.21–5.27 (m, 2H), 5.86 (ddd, *J* = 16.9, 10.1, 7.9 Hz, 1H), 7.08 (ddd, *J* = 7.4, 7.4, 0.8 Hz, 1H), 7.16 (d, *J* = 7.4 Hz, 1H), 7.22–7.28 (m, 1H), 7.43 (d, *J* = 8.0 Hz, 1H); ^13^C NMR (100.61 MHz, CDCl_3_) *δ* 35.1 (CH_3_), 45.3 (CH), 56.5 (CH_2_), 114.4 (CH), 118.1 (CH_2_), 124.4 (CH), 125.9 (CH), 129.1 (CH), 133.8 (C), 137.7 (CH), 142.2 (C); GC–MS retention time 15.68 min, *m/z* (EI) 223 (M^+•^, 30%), 144.1 (100), 143 (37), 128 (10), 115 (50), 103 (4), 91 (12), 89 (15).

**Diphenyl disulfide (33)** [7,10-12]: mp 55–56 °C (lit. [10] mp 55–56 °C) [Found: M^+•^ (EI), 218.0215. C_12_H_10_S_2_ requires *M*, 218.0218]; IR (KBr disc) 3070, 3055, 2925, 2854, 1575, 1474, 1437, 1072, 1022, 996, 738 cm^−1^; ^1^H NMR (400.13 MHz, CDCl_3_) *δ* 7.22–7.27 (2H, m, ArH), 7.29–7.35 (4H, m, ArH), 7.49–7.54 (4H, m, ArH); ^13^C NMR (100.61 MHz, CDCl_3_) *δ* 127.4 (CH), 127.8 (CH), 129.3 (CH), 137.3 (C); *m/z* (EI) 218 (M^+•^, 14%), 184 (10), 140 (6), 109 (100), 82 (14), 77 (52), 69 (40), 65 (38), 57 (21), 51 (53).

**Octamethyloxamidinium bis(tetrafluoroborate) (21):** mp 230 °C (dec). The melting point and the spectroscopic data of the salt **21** were identical with the data of the same salt reported previously in the experimental section.

**Representative General Procedure C [Table 2, entry (iii)]:** A 25 ml two-necked round bottomed flask furnished with a magnetic stirring bar and a rubber septum was flame-dried *in vacuo*, backfilled with dry argon gas. This procedure was repeated at least thrice and the reaction vessel was transported to a glove box (oxygen and moisture levels inside the glove box were maintained at 0–2 ppm at all times) under a nitrogen atmosphere. TDAE (0.140 g, 0.697 mmol, 3.0 equiv) was added to the reaction vessel, followed by addition of a solution of 2-[methanesulfonyl-{(*Z*)-4-phenylsulfanylbut-2-enyl}amino]benzenediazonium tetrafluoroborate (**31a**, 0.104 g, 0.2324 mmol, 1.0 equiv) in dry, freshly distilled and deoxygenated acetonitrile (3 ml, degassed by purging with a stream of argon gas for 30 min at 25 °C prior to its transportation to a glove box). Immediate evolution of nitrogen gas bubbles was observed whilst the reaction mixture turned deep red in colour which gradually turned to orange with precipitation of a brown precipitate. After vigorous stirring of the reaction mixture for 5 min at 25 °C under a nitrogen atmosphere, the reaction vessel was transported outside the glove box. The reaction mixture was evaporated to dryness under reduced pressure and the crude residue was dissolved in a mixture of deionised water (25 ml) and ethyl acetate (50 ml) and stirred in open air for 30 min. The organic layer was separated and the aqueous layer was washed with further ethyl acetate (25 ml). The combined organic phases were dried over anhydrous sodium sulfate, filtered, evaporated to dryness to obtain a brownish yellow residue. This was purified by flash chromatography (ethyl acetate/petroleum ether = 1:99) on silica to afford diphenyl disulfide (**33**) [7,10-12] as a white waxy solid (0.010 g, 39%) and 1-methanesulfonyl-3-vinyl-2,3-dihydro-1*H*-indole [7] (**20a**, eluant: ethyl acetate/petroleum ether = 1:9) as a yellow solid (0.044 g, 85%). The melting points and the spectroscopic data of diphenyl disulfide (**33**) [7,10-12] and 1-methanesulfonyl-3-vinyl-2,3-dihydro-1*H*-indole (**20a**) [7] were identical with those reported previously in this experimental section and with those reported in the literature.

**Representative General Procedure D [entry (vi)]:** A solution of *N*-(2-aminophenyl)-*N*-[(*Z*)-4-benzenesulfinylbut-2-enyl]methanesulfonamide (**30b**, 0.1203 g, 0.33 mmol, 1.0 equiv) dissolved in dry freshly distilled dichloromethane (10 ml) was loaded in to a dry 25 ml two-necked round bottomed flask furnished with a magnetic stirring bar and a rubber septum under an argon atmosphere. The solution was cooled to −10 °C and nitrosonium tetrafluoroborate (0.046 g, 0.396 mmol, 1.2 equiv) was added to it. The reaction mixture was stirred under an argon atmosphere for 1.5 h at −10 °C. When the reaction was judged to be complete by TLC analysis, the solvent was carefully removed under reduced pressure at 0 °C to afford 2-[{(*Z*)-4-benzenesulfinylbut-2-enyl}methanesulfonylamino]benzenediazonium tetrafluoroborate (**31b**) as a reddish brown sticky semi-solid. This was immediately dissolved in degassed acetone (5 ml, reagent grade, degassed by purging with a slow stream of argon gas for 30 min at r.t.) under an argon atmosphere and reacted with TDAE (0.077 ml, 0.33 mmol, 1.0 equiv) at 25 °C for 5 min under an argon atmosphere following general procedure A [as described previously for the cyclization of 2-[methanesulfonyl-{(*Z*)-4-phenylsulfanylbut-2-enyl}amino]benzenediazonium tetrafluoroborate (**31a**) by TDAE for entry (i) of Table 2] to afford octamethyloxamidinium bis(tetrafluoroborate) salt (**21**, 0.015 g, 24%) as a dull white powder, mp 230 °C (dec) and the cyclised product 1-methanesulfonyl-3-vinyl-2,3-dihydro-1*H*-indole [7] (**20a**, 0.060 g, 81%) as a yellow solid [eluant: ethyl acetate/petroleum ether = 1:9], mp 52–54°C. The melting points and the spectroscopic data of **21** and **20a** [7] were identical with those reported previously in this experimental section and with those reported in the literature.

Data for 1-methanesulfonyl-5-methoxy-3-vinyl-2,3-dihydro-1*H*-indole **32** [obtained by cyclization of arenediazonium salt **31d** by TDAE, entry (viii) of Table 2, following general procedure D]: mp 58–59 °C [Found: MNH_4_^+^ (ESI), 271.1116. C_12_H_15_NO_3_S requires *MNH_4_*, 271.1111]; IR (KBr disc) 3011, 2926, 2884, 2835, 1646, 1608, 1489, 1339, 1290, 1255, 1233, 1215, 1192, 1180, 1155, 1031, 988, 965, 929, 846, 829 cm^−1^; ^1^H NMR (400.13 MHz, CDCl_3_) *δ* 2.82 (s, 3H), 3.67 (dd, *J* = 10.8, 8.0 Hz, 1H), 3.78 (s, 3H), 3.95–4.04 (m, 1H), 4.19 (dd, *J* = 10.8, 9.2 Hz, 1H), 5.21–5.29 (m, 2H), 5.84 (ddd, *J* = 17.0, 10.0, 8.0 Hz, 1H), 6.71 (d, *J* = 2.2 Hz, 1H), 6.77 (dd, *J* = 8.7, 2.2 Hz, 1H), 7.34 (d, *J* = 8.7 Hz, 1H); ^13^C NMR (100.61 MHz, CDCl_3_) *δ* 34.7 (CH_3_), 45.7 (CH), 56.2 (CH_3_), 56.9 (CH_2_), 111.8 (CH), 114.1 (CH), 115.8 (CH), 118.3 (CH_2_), 135.6 (2 × C), 137.6 (CH), 157.5 (C); *m/z* (CI) 271 [(M+NH_4_)^+^, 100%], 254 (12), 253 (6), 218 (3), 177 (11), 175 (42), 160 (4), 148 (5), 131 (3), 52 (14).

**Experimental Section III**

**[(*Z*)-4-Bromobut-2-enyloxy]-*tert*-butyldiphenylsilane (34)** [13]

Methyl sulfide (1.32 ml, 18.0 mmol, 1.8 equiv) was added dropwise to a solution of *N*-bromosuccinimide (2.670 g, 15.0 mmol, 1.5 equiv) in dry dichloromethane (100 ml) at 0 °C under a nitrogen atmosphere and stirred for 30 min at −30 °C to form a yellow complex. To this suspension was added a solution of (*Z*)-4-(*tert*-butyldiphenylsilanyloxy)but-2-en-1-ol (**24**, 3.265 g, 10.0 mmol, 1.0 equiv) in dry dichloromethane (50 ml) and the reaction mixture was slowly brought to room temperature and stirred under nitrogen for 3 h. When the reaction was judged to be complete by TLC analysis, dichloromethane (100 ml) was added to the reaction mixture and the contents were washed with brine solution (3 × 100 ml) and deionised water (3 × 100 ml). The organic layer was separated, dried over anhydrous magnesium sulfate and evaporated to dryness under reduced pressure to obtain a colourless oil which was purified by flash chromatography (eluant: ethyl acetate/petroleum ether = 1:9) on silica gel to obtain [(*Z*)-4-bromobut-2-enyloxy]-*tert*-butyldiphenylsilane [7,13] (**34**, 3.695 g, 95%) as a clear colourless oil which was immediately carried on to the next reaction [Found: (M−Br)^+^ (ESI), 309.1671. C_20_H_25_OSiBr requires 309.1669]; IR (neat film, NaCl) 3071, 2959, 2931, 2858, 1472, 1428, 1207, 1112, 1063, 824, 739, 702 cm^−1^; ^1^H NMR (400.13 MHz, CDCl_3_) *δ* 1.12 (s, 9H), 3.86–3.91 (m, 2H), 4.37 (d, *J* = 3.9 Hz, 2H), 5.75–5.80 (m, 2H), 7.42–7.48 (m, 6H), 7.71–7.76 (m, 4H); ^13^C NMR (100.61 MHz, CDCl_3_) *δ* 19.3 (C), 26.97 (CH_2_), 26.99 (CH_3_), 59.9 (CH_2_), 126.5 (CH), 128.0 (CH), 130.0 (CH), 133.6 (C), 133.9 (CH), 135.8 (CH); *m/z* (CI) 389 [(M+H)^+^, 5%], 309 (100), 267 (7), 251 (4), 231 (6), 205 (5), 167 (3), 131 (24).

**(*Z*)-4-[(*Z*)-4-(*tert*-Butyldiphenylsilanyloxy)but-2-enyloxy]but-2-en-1-ol (35)**

Sodium hydride (2.296 g of 60% NaH dispersed in mineral oil, 57.4 mmol, 4.1 equiv) was washed with petroleum ether (2 × 10 ml) and suspended in dry THF (50 ml) at 0 °C under nitrogen. To this mixture was added *cis-*2-butene-1,4-diol (**23**, 4.61 ml, 56.0 mmol, 4.0 equiv) at 0 °C. Rapid hydrogen evolution occurred. The reaction mixture was brought to r.t. and stirred for 1 h and [(*Z*)-4-bromobut-2-enyloxy]-*tert*-butyldiphenylsilane (**34**, 5.4516 g, 14.0 mmol, 1.0 equiv) dissolved in dry tetrahydrofuran (50 ml) was added. The mixture was stirred at r.t. under nitrogen for 72 h. When the reaction was judged to be complete by TLC, the reaction mixture was evaporated to dryness to obtain a yellow-brown semi-solid, which was extracted with diethyl ether (3 × 200 ml) and water (3 × 250 ml). The combined organic extracts were collected and washed with brine solution (2 × 250 ml), dried over anhydrous sodium sulfate, filtered, evaporated to dryness under reduced pressure to obtain a colourless viscous oil. This was purified by flash chromatography on silica gel [eluant: ethyl acetate/petroleum ether = 3:7] to obtain (*Z*)-4-[(*Z*)-4-(*tert*-butyldiphenylsilanyloxy)but-2-enyloxy]but-2-en-1-ol [7] (**35**, 3.368 g, 61%) as a colourless oil [Found: MH^+^ (ESI), 397.2199. C_24_H_32_O_3_Si requires *MH*, 397.2199]; IR (neat film, NaCl) 3424, 3071, 3026, 2958, 2931, 2858, 1742, 1472, 1463, 1428, 1235, 1112, 1075, 1030, 824, 741, 703 cm^−1^; ^1^H NMR (400.20 MHz, CDCl_3_) *δ* 0.97 (s, 9H),1.18 (s, 1H), 3.81 (d, *J* = 6.4 Hz, 2H), 3.86 (d, *J* = 6.3 Hz, 2H), 4.06 (d, *J* = 6.4 Hz, 2H), 4.18 (dd, *J* = 6.0, 0.6 Hz, 2H), 5.49 (dtt, *J* = 11.2, 6.4, 1.6 Hz, 1H), 5.55 (dtt, *J* = 11.2, 6.3, 1.3 Hz, 1H), 5.66–5.74 (dm, *J* = 10.2 Hz, 2H), 7.29–7.36 (m, 6H), 7.59–7.63 (m, 4H); ^13^C NMR (100.63 MHz, CDCl_3_) *δ* 19.2 (C), 26.8 (CH_3_), 58.7 (CH_2_), 60.4 (CH_2_), 65.5 (CH_2_), 66.0 (CH_2_), 126.9 (CH), 127.7 (CH), 128.3 (CH), 129.7 (CH), 132.37 (CH), 132.42 (CH), 133.6 (C), 135.6 (CH); *m/z* (ESI) 414 [(M+NH_4_)^+^, 100%], 397 (31), 327 (4), 249 (10), 131 (4), 71 (3).

***N*-[(*Z*)-4-{(*Z*)-4-(*tert*-Butyldiphenylsilanyloxybut-2-enyloxy}but-2-enyl]-*N*-(2-nitrophenyl)meth-anesulfonamide (36)**

Following the experimental procedure described for the preparation of *N*-[(*Z*)-4-(*tert*-butyldiphenylsilanyloxy)but-2-enyl]-*N*-(2-nitrophenyl)methanesulfonamide (**26a**), *N*-(2-nitrophenyl)-methanesulfonamide (**25a**, 0.865 g, 4.0 mmol, 1.0 equiv) was reacted with triphenylphosphine (1.574 g, 6.0 mmol, 1.5 equiv), (*Z*)-4-[(*Z*)-4-(*tert*-butyldiphenylsilanyloxy)but-2-enyloxy]but-2-en-1-ol (**35**, 1.586 g, 4.0 mmol, 1.0 equiv) and diisopropyl azodicarboxylate (1.19 ml, 6.0 mmol, 1.5 equiv) in freshly distilled dry tetrahydrofuran (100 ml) at r.t. for 1 h under a nitrogen atmosphere to afford *N*-[(*Z*)-4-{(*Z*)-4-(*tert*-butyldiphenylsilanyloxy)but-2-enyloxy}but-2-enyl]-*N*-(2-nitrophenyl)methanesulfonamide [7] (**36**, 2.325 g, 98%) [purification by flash chromatography on silica; eluant: ethyl acetate/toluene = 1:3] as a yellow viscous liquid [Found: MNH_4_^+^ (ESI), 612.2569. C_31_H_38_N_2_O_6_SSi requires *MNH_4_*, 612.2564]; IR (neat film, NaCl) 3071, 3028, 2931, 2857, 1604, 1581, 1534, 1485, 1473, 1428, 1347, 1224, 1156, 1111, 1069, 1008, 961, 873, 845, 825, 783, 743, 704 cm^−1^; ^1^H NMR (400.13 MHz, CDCl_3_) *δ* 1.06 (s, 9H), 3.01 (s, 3H), 3.71 (d, *J* = 6.3 Hz, 2H), 3.75 (d, *J* = 4.4 Hz, 2H), 4.22 (d, *J* = 6.0 Hz, 2H), 4.31 (broad s, 2H), 5.47 (dtt, *J* = 11.3, 6.3, 1.6 Hz, 1H), 5.63–5.70 (m, 2H), 5.76 (dtt, *J* = 11.3, 6.0, 1.5 Hz, 1H), 7.38–7.50 (m, 8H), 7.56 (ddd, *J* = 8.0, 7.5, 1.5 Hz, 1H), 7.67–7.72 (m, 4H), 7.84 (dd, *J* = 8.0, 1.5 Hz, 1H); ^13^C NMR (100.61 MHz, CDCl_3_) *δ* 19.6 (C), 27.3 (CH_3_), 41.0 (CH_3_), 48.8 (CH_2_), 60.8 (CH_2_), 65.7 (CH_2_), 66.6 (CH_2_), 125.7 (CH), 126.8 (CH), 127.2 (CH), 128.2 (CH), 130.0 (CH), 130.2 (CH), 132.2 (CH), 132.5 (C), 132.78 (CH), 133.82 (CH), 134.1 (C), 134.2 (CH), 136.1 (CH), 149.1 (C); *m/z* (FAB) 617 [(M+Na)^+^, 29%], 595 [(M+H)^+^, 7%], 309 (33), 269 (81), 190 (100), 135 (73).

***N*-[(*Z*)-4-{(*Z*)-4-Hydroxybut-2-enyloxy}but-2-enyl]-*N*-(2-nitrophenyl)methanesulfonamide (37)**

Following an experimental procedure identical to that described for the preparation of *N*-[(*Z*)-4-hydroxybut-2-enyl]-*N*-(2-nitrophenyl)methanesulfonamide (**27a**), *N*-{(*Z*)-4-[(*Z*)-4-(*tert*butyldiphenyl-silanyloxy)but-2-enyloxy]but-2-enyl}-*N*-(2-nitrophenyl)methanesulfonamide (**36**, 2.2305 g, 3.75 mmol, 1.0 equiv) was reacted with tetrabutylammonium fluoride (5.63 ml of 1 M solution in THF, 5.625 mmol, 1.5 equiv) in tetrahydrofuran (100 ml) at r.t. for 20 min under a nitrogen atmosphere to afford *N*-[(*Z*)-4-{(*Z*)-4-hydroxybut-2-enyloxy}but-2-enyl]-*N*-(2-nitrophenyl)methanesulfonamide [7] (**37**, 1.207 g, 90%) [purification by flash chromatography on silica; eluant: dichloromethane/ethyl acetate = 2:3] as a pale yellow viscous oil [Found: MH^+^ (ESI), 357.1116. C_15_H_20_N_2_O_6_S requires *MH*, 357.1120]; (Found: C, 50.24; H, 5.87; N, 7.78. C_15_H_20_N_2_O_6_S requires C, 50.55; H, 5.66; N, 7.86%); IR (neat film, NaCl) 3409, 3025, 2931, 2871, 1604, 1581, 1533, 1485, 1411, 1343, 1224, 1155, 1094, 1065, 1027, 963, 874, 846, 784, 730, 706 cm^−1^; ^1^H NMR (400.13 MHz, CDCl_3_) *δ* 2.08 (s, 1H), 3.01 (s, 3H), 3.89 (d, *J* = 4.7 Hz, 2H), 3.91 (d, *J* = 6.4 Hz, 2H), 4.14 (d, *J* = 6.4 Hz, 2H), 4.34 (broad s, 2H), 5.57 (dtt, *J* = 11.3, 6.4, 1.3 Hz, 1H), 5.65–5.81 (m, 3H), 7.48–7.54 (m, 2H), 7.64 (ddd, *J* = 7.7, 7.7, 1.5 Hz, 1H), 7.90 (dd, *J* = 8.0, 1.5 Hz, 1H); ^13^C NMR (100.61 MHz, CDCl_3_) *δ* 40.9 (CH_3_), 48.8 (CH_2_), 59.0 (CH_2_), 65.9 (CH_2_), 66.4 (CH_2_), 125.8 (CH), 127.1 (CH), 128.3 (CH), 130.1 (CH), 132.0 (CH), 132.5 (C), 133.0 (CH), 133.96 (CH), 134.03 (CH), 149.1 (C); *m/z* (FAB), 379 [(M+Na)^+^, 60%], 357 [(M+H)^+^, 63%], 269 (89), 190 (95), 186 (100).

***N*-[(*Z*)-4-{(*Z*)-4-Bromo-but-2-enyloxy}but-2-enyl]-*N*-(2-nitrophenyl)methanesulfonamide (38)**

*N*-Bromosuccinimide (1.2175 g, 6.84 mmol, 1.2 equiv) was added to a solution of *N*-[(*Z*)-4-{(*Z*)-4-hydroxybut-2-enyloxy}but-2-enyl]-*N*-(2-nitrophenyl)methanesulfonamide (**37**, 2.0315 g, 5.7 mmol, 1.0 equiv) and triphenylphosphine (1.6445 g, 6.27 mmol, 1.1 equiv) in dry dichloromethane (100 ml) at −25 °C. The reaction mixture was stirred under nitrogen for 20 min at −25 °C, then warmed to r.t. and stirring was continued for further 40 min. When the reaction was complete, the reaction mixture turned pale violet from yellow and the contents were extracted with dichloromethane (3 × 50 ml) and water (3 × 100 ml). The combined organic phases was dried over anhydrous sodium sulfate and evaporated to dryness to obtain a violet semi-solid which was purified by flash chromatography on silica gel using dichloromethane as the eluant to afford *N***-**[(*Z*)-4-{(*Z*)-4-bromo-but-2-enyloxy}but-2-enyl]-*N*-(2-nitrophenyl)methanesulfonamide [7] (**38**, 2.304 g, 96%) as a viscous pale yellow oil [Found: MNH_4_^+^ (ESI), 436.0541. C_15_H_19_N_2_O_5_S^79^Br requires *MNH_4_*, 436.0542]; IR (neat film, NaCl) 3031, 2930, 2859, 1603, 1581, 1533, 1485, 1448, 1405, 1344, 1209, 1155, 1095, 1064, 1009, 961, 873, 846, 783, 731, 707 cm^−1^; ^1^H NMR (400.13 MHz, CDCl_3_) *δ* 3.03 (s, 3H), 3.88 (d, *J* = 4.6 Hz, 2H), 3.94–3.99 (m, 4H), 4.35 (broad s, 2H), 5.61 (dt, *J* = 11.1, 6.2 Hz, 1H), 5.68–5.76 (m, 2H), 5.81–5.92 (dm, *J* = 10.8 Hz, 1H), 7.49–7.56 (m, 2H), 7.64 (ddd, *J* = 7.8, 7.7, 1.5 Hz, 1H), 7.91 (dd, *J* = 8.0, 1.5 Hz, 1H); ^13^C NMR (100.61 MHz, CDCl_3_) *δ* 26.9 (CH_2_), 41.0 (CH_3_), 48.9 (CH_2_), 65.7 (CH_2_), 66.1 (CH_2_), 125.8 (CH), 127.2 (CH), 129.0 (CH), 130.1 (CH), 131.2 (CH), 131.9 (CH), 132.5 (C), 133.9 (CH), 134.3 (CH), 149.1 (C); *m/z* (FAB) 443 [{M(^81^Br)+Na}^+^, 17%], 441 [{M(^79^Br)+Na}^+^, 17%], 419 (21), 269 (52), 190 (64), 186 (100), 135 (25).

***N*-(2-Nitrophenyl)-*N*-[(*Z*)-4-{(*Z*)-4-phenylsulfanylbut-2-enyloxy}but-2-enyl]methanesulfonamide (39)**

Following an experimental procedure identical to that described for the preparation of *N*-(2-nitrophenyl)-*N*-[(*Z*)-4-phenylsulfanylbut-2-enyl]methanesulfonamide (**29a**), thiophenol (0.51 ml, 4.95 mmol, 1.1 equiv) was treated with sodium hydride [0.216 g of 60% NaH dispersed in mineral oil, 5.4 mmol, 1.2 equiv, washed with dry hexane (2 × 10 ml) prior to use] in dry tetrahydrofuran (25 ml) at 0 °C to r.t. for 1 h, and reacted with *N***-**[(*Z*)-4-{(*Z*)-4-bromobut-2-enyloxy}but-2-enyl]-*N*-(2-nitrophenyl)methanesulfonamide (**38**, 1.8868 g, 4.5 mmol, 1.00 equiv) in dry tetrahydrofuran (75 ml) for 12 h at r.t. to afford a brown semi-solid which after work-up yielded a yellow-brown oil. This was purified by flash chromatography on silica gel [eluant: ethyl acetate/petroleum ether = 3:7] to afford *N*-(2-nitrophenyl)-*N*-[(*Z*)-4-{(*Z*)-4-phenylsulfanylbut-2-enyloxy}but-2-enyl]methanesulfonamide [7] (**39**, 1.741 g, 86%) as a viscous yellow oil [Found: MNH_4_^+^ (ESI), 466.1484. C_21_H_24_N_2_O_5_S_2_ requires *MNH_4_*, 466.1470]; (Found: C, 55.93; H, 5.2 7; N, 6.50. C_21_H_24_N_2_O_5_S_2_ requires C, 56.23; H, 5.39; N, 6.25%); IR (neat film, NaCl) 3025, 2930, 2860, 1603, 1582, 1532, 1482, 1439, 1410, 1344, 1224, 1155, 1095, 1025, 1010, 961, 873, 846, 783, 742 cm^−1^; ^1^H NMR (400.13 MHz, CDCl_3_) *δ* 3.02 (s, 3H), 3.54 (d, *J* = 7.7 Hz, 2H), 3.78 (m, 4H), 4.33 (broad s, 2H), 5.55 (dt, *J* = 10.9, 6.4 Hz, 1H), 5.63–5.75 (m, 3H), 7.18–7.25 (m, 1H), 7.26–7.32 (m, 2H), 7.33–7.40 (m, 2H), 7.47–7.55 (m, 2H), 7.62 (ddd, *J* = 8.0, 7.7, 1.5 Hz, 1H), 7.89 (dd, *J* = 8.0, 1.6 Hz, 1H); ^13^C NMR (100.61 MHz, CDCl_3_) *δ* 31.8 (CH_2_), 40.7 (CH_3_), 48.5 (CH_2_), 65.5 (CH_2_), 65.8 (CH_2_), 125.5 (CH), 126.6 (CH), 126.9 (CH), 128.4 (CH), 129.1 (CH), 129.2 (CH), 129.7 (CH), 130.7 (CH), 131.8 (CH), 132.2 (C), 133.6 (CH), 133.9 (CH), 135.8 (C), 148.7 (C); *m/z* (CI) 466 [(M+NH_4_)^+^, 33%], 163 (100), 119 (26), 109 (15).

***N*-[4-(4-Benzenesulfonylbut-2-enyloxy)but-2-enyl]-*N*-(2-nitrophenyl)methanesulfonamide (40)**

Following the experimental procedure identical to that described for the preparation of *N*-[(*Z*)-4-benzenesulfonylbut-2-enyl]-*N*-(2-nitrophenyl)methanesulfonamide (**29c**), *N*-(2-nitrophenyl)-*N*-[4-{4-phenylsulfanylbut-2-enyloxy}but-2-enyl]methanesulfonamide (**39**, 0.9868**,** 2.2 mmol, 1.0 equiv) dissolved in methanol (60 ml) and deionised water (30 ml) was reacted with sodium periodate (2.353 g, 11.0 mmol, 5.0 equiv) at r.t. under a nitrogen atmosphere for 76 h to afford *N*-[4-{4-benzenesulfonyl-but-2-enyloxy}but-2-enyl]-*N*-(2-nitrophenyl)methanesulfonamide [7] [**40**, purification by flash chromatography on silica; eluant: ethyl acetate/dichloromethane = 3:7] as a viscous yellow oil (0.771 g, 73%), identified as a mixture of geometrical isomers from its ^13^C NMR spectrum [Found: MNH_4_^+^ (ESI), 498.1365. C_21_H_24_N_2_O_7_S_2_ requires *MNH_4_*, 498.1369]; (Found: C, 52.51; H, 4.98; N, 5.74. C_21_H_24_N_2_O_7_S_2_ requires C, 52.49; H, 5.03; N, 5.83%); IR (neat film, NaCl) 3067, 3028, 2931, 2863, 1604, 1583, 1533, 1485, 1448, 1410, 1344, 1307, 1233, 1144, 1086, 962, 888, 874, 846, 784, 759, 729 cm^−1^; ^1^H NMR (400.13 MHz, CDCl_3_) *δ* 2.99 (s, 3H), 3.66 (dd, *J* = 6.0, 1.6 Hz, 2H), 3.74 (d, *J* = 5.7 Hz, 2H), 3.88 (d, *J* = 8.0 Hz, 2H), 4.30 (broad s, 2H), 5.51 (dtt, *J* = 11.2, 8.0, 1.6 Hz, 1H), 5.58–5.72 (m, 2H), 5.79 (dt, *J* = 11.2, 6.0 Hz, 1H), 7.46–7.58 (m, 4H), 7.60–7.67 (m, 2H), 7.84–7.90 (m, 3H); ^13^C NMR (100.61 MHz, CDCl_3_) *δ* (major + minor) 40.2 (CH_3_), 40.3 (CH_3_), 48.4 (CH_2_), 55.4 (CH_2_), 59.7 (CH_2_), 65.3 (CH_2_), 65.6 (CH_2_), 66.0 (CH_2_), 69.6 (CH_2_), 69.8 (CH_2_), 118.3 (CH), 118.5 (CH), 125.5 (CH), 126.7 (CH), 126.8 (CH), 128.4 (CH), 128.5 (CH), 129.3 (CH), 129.7 (CH), 129.8 (CH), 131.18 (CH), 131.24 (CH), 132.1 (C), 133.2 (CH), 133.3 (CH), 133.6 (CH), 134.0 (CH), 135.6 (CH), 137.0 (CH), 138.5 (C), 148.7 (C); *m/z* (CI) 498 [(M+NH_4_)^+^, 4%], 311 (30), 212 (98), 187 (100), 119 (50), 109 (41).

***N*-(2-Aminophenyl)-*N*-[(*Z*)-4-{(*Z*)-4-phenylsulfanylbut-2-enyloxy}but-2-enyl]methanesulfonamide (41)**

Following an experimental procedure identical to that described for the preparation of *N*-(2-amino-4-methoxyphenyl)-*N*-[(*Z*)-4-phenylsulfanylbut-2-enyl]methanesulfonamide (**30d**), *N*-(2-nitrophenyl)-*N*-[(*Z*)-4-{(*Z*)-4-phenylsulfanylbut-2-enyloxy}but-2-enyl]methanesulfonamide (**39**, 1.570 g, 3.5 mmol, 1.0 equiv) dissolved in dichloromethane (2 ml), methanol (125 ml) and water (1 ml) was reacted with stannous chloride (3.3183 g, 17.5 mmol, 5.0 equiv) at 65 °C for 4 h under a nitrogen atmosphere to provide *N*-(2-aminophenyl)-*N*-[(*Z*)-4-{(*Z*)-4-phenylsulfanylbut-2-enyloxy}but-2-enyl]methane­sulfon­amide [7] [**41**, purification by flash chromatography on silica; eluant: hexane/dichloromethane = 1:4 to 100% dichloromethane] as a pale yellow sticky semi-solid (1.397 g, 95%) [Found: MH^+^ (ESI), 419.1467. C_21_H_26_N_2_O_3_S_2_ requires *MH*, 419.1463]; IR (neat film, NaCl) 3474, 3378, 3057, 3025, 2928, 2859, 1619, 1583, 1500, 1481, 1459, 1439, 1334, 1269, 1229, 1209, 1151, 1089, 1072, 1025, 1009, 959, 881, 854, 825, 747 cm^−1^; ^1^H NMR (400.13 MHz, CDCl_3_) *δ* 2.99 (s, 3H), 3.54 (d, *J* = 7.7 Hz, 2H), 3.76 (d, *J* = 6.3 Hz, 2H), 3.83 (d, *J* = 4.1 Hz, 2H), 3.99 (broad s, 2H), 4.25 (d, *J* = 5.2 Hz, 2H), 5.60 (dt, *J* = 11.0, 6.3 Hz, 1H), 5.64–5.74 (m, 3H), 6.72 (m, 1H), 6.78 (d, *J* = 8.0 Hz, 1H), 7.07–7.17 (m, 2H), 7.19–7.23 (m, 1H), 7.26–7.32 (m, 2H), 7.36 (d, *J* = 7.9 Hz, 2H); ^13^C NMR (100.61 MHz, CDCl_3_) *δ* 32.1 (CH_2_), 38.8 (CH_3_), 48.2 (CH_2_), 65.9 (2 × CH_2_), 117.5 (CH), 119.0 (CH), 125.0 (C), 127.1 (CH), 127.2 (CH), 128.7 (CH), 129.2 (CH), 129.4 (CH), 129.6 (CH), 130.4 (CH), 131.0 (CH), 131.5 (CH), 136.2 (C), 147.0 (C); *m/z* (CI) 419 [(M+H)^+^, 20%], 311 (45), 257 (27), 187 (100), 109 (36), 52 (16).

***N*-(2-Aminophenyl)-*N*-[4-(4-benzenesulfonylbut-2-enyloxy)but-2-enyl]methanesulfonamide (43)**

Following an experimental procedure identical to that described for the preparation of *N*-(2-amino-4-methoxyphenyl)-*N*-[(*Z*)-4-phenylsulfanylbut-2-enyl]methanesulfonamide (**30d**), *N*-[4-{4-benzene­sulfonylbut-2-enyloxy}but-2-enyl]-*N*-(2-nitrophenyl)methanesulfonamide (**40**, 0.6247 g, 1.3 mmol, 1.0 equiv) dissolved in dichloromethane (1 ml), methanol (15 ml) was reacted with stannous chloride dihydrate (1.4666 g, 6.5 mmol, 5.0 equiv) at 65 °C for 3.5 h under a nitrogen atmosphere at r.t. to afford *N*-(2-aminophenyl)-*N*-[(*Z*)-4-{(*Z*)-4-benzenesulfonylbut-2-enyloxy}but-2-enyl]methanesulfonamide [7] [**43**, purification by flash chromatography on silica; eluant: ethyl acetate/dichloromethane = 3:7] as a yellow sticky semi-solid (0.457 g, 78%), identified as a 1:9 mixture of geometrical isomers from its ^1^H and ^13^C NMR spectra [Found: MH^+^ (ESI), 451.1361. C_21_H_26_N_2_O_5_S_2_ requires *MH*, 451.1361]; IR (neat film, NaCl) 3479, 3378, 3063, 3028, 2929, 2858, 1621, 1585, 1501, 1457, 1447, 1410, 1333, 1314, 1307, 1237, 1210, 1148, 1085, 960, 883, 825, 752, 729 cm^−1^; ^1^H NMR (400.13 MHz, CDCl_3_) *δ* 2.97 (major) and 2.98 (minor) (each s, 3H), 3.65 (dd, *J* = 6.0, 1.3 Hz, 2H), 3.75 (d, *J* = 5.4 Hz, 2H), 3.89 (d, *J* = 8.0 Hz, 2H), 3.98 (broad s, 2H), 4.21 (d, *J* = 6.9 Hz, 2H), 5.48–5.71 (m, 3H), 5.80–5.83 (m, 1H), 6.72 (ddd, *J* = 8.0, 7.4, 1.1 Hz, 1H), 6.78 (dd, *J* = 8.0, 1.3 Hz, 1H), 7.07–7.16 (m, 2H), 7.52–7.59 (m, 2H), 7.61–7.68 (m, 1H), 7.84–7.89 (m, 2H); ^13^C NMR (100.61 MHz, CDCl_3_) *δ* (major + minor) 38.6 (CH_3_), 38.7 (CH_3_), 48.0 (CH_2_), 48.1 (CH_2_), 55.8 (CH_2_), 60.1 (CH_2_), 65.6 (CH_2_), 66.0 (CH_2_), 66.1 (CH_2_), 69.7 (CH_2_), 117.5 (CH), 118.5 (CH), 118.7 (CH), 118.9 (CH), 124.8 (C), 127.5 (CH), 127.7 (CH), 128.7 (CH), 128.9 (CH), 129.1 (CH), 129.2 (CH), 129.6 (CH), 130.29 (CH), 130.34 (CH), 130.9 (CH), 131.0 (CH), 134.3 (CH), 136.0 (CH), 137.5 (CH), 138.9 (C), 147.0 (C); *m/z* (CI) 451 [(M+H)^+^, 55%], 311 (68), 204 (35), 187 (100).

**Cascade radical cyclization of 2-{methanesulfonyl-[(*Z*)-4-{(*Z*)-4-phenylsulfanylbut-2-enyloxy}but-2-enyl]amino}benzenediazonium tetrafluoroborate (42) by TDAE (1).**

Following an experimental procedure [General procedure D, Table 2, entry (vi)] identical to that described for the cyclization of 2-[{(*Z*)-4-benzenesulfinylbut-2-enyl}methanesulfonylamino]benzene­diazonium tetrafluoroborate (**31b**) by TDAE, *N*-(2-aminophenyl)-*N*-[(*Z*)-4-{(*Z*)-4-phenylsulfanylbut-2-enyloxy}but-2-enyl]methanesulfonamide (**41**, 0.3495 g, 0.835 mmol, 1.0 equiv) was first diazotized with nitrosonium tetrafluoroborate (0.1072 g, 0.919 mmol, 1.1 equiv) in dichloromethane (20 ml) at −10 °C for 1.5 h under an argon atmosphere to obtain the diazonium salt **42** as a reddish brown semi-solid. The diazonium salt **42** was immediately dissolved in degassed acetone (5 ml) and reacted with TDAE (0.19 ml, 0.835 mmol, 1.0 equiv) at r.t. under an argon atmosphere for 10 min, to afford octamethyloxamidinium bis(tetrafluoroborate) (**21**, 0.081 g, 52%) as a pale white powder, mp 230 °C (dec) and a yellow semi-solid (after work-up), which was purified by flash chromotography on silica gel to afford diphenyl disulfide [7,10-12] (**33**, 0.0422 g, 46%) as a pale white waxy solid [eluant: ethyl acetate/petroleum ether = 1:99], mp 55–56 °C (lit. [10] mp 55–56 °C) along with the expected bicyclised product, 1-methanesulfonyl-3-(4-vinyltetrahydrofuran-3-yl)-2,3-dihydro-1*H*-indole [7] (**47**, 0.208 g, 85% in two steps, from **41**) [eluant: ethyl acetate/petroleum ether = 3:7] as a yellow semi-solid. The bicylised product **47** was isolated as a mixture of 4 diastereoisomers in the ratio 1:4:1.25:3, determined from its ^1^H NMR spectrum.

**1-Methanesulfonyl-3-(4-vinyltetrahydrofuran-3-yl)-2,3-dihydro-1*H*-indole (47)** [7]: [Found: MNH_4_^+^ (ESI), 311.1422. C_15_H_19_NO_3_S requires *MNH_4_*, 311.1429]; IR (neat film, NaCl) 3075, 2930, 2861, 1639, 1600, 1480, 1459, 1348, 1241, 1160, 1106, 1057, 1031, 997, 965, 923, 771, 755 cm^−1^; ^1^H NMR (400.13 MHz, CDCl_3_) *δ* 2.31–2.75 (3 × m, total 1H), 2.88 (s, 3H), 2.94–3.40 (3 × m, total 1H), 3.45–3.59 (m, 1H), 3.60–3.72 (m, 1H), 3.75–3.88 (m, 2H), 3.89–4.09 (2 × m, total 3H), 4.98–5.33 (4 × m, total 2H), 5.55–5.98 (4 × m, total 1H), 6.95–7.44 (3 × m, total 4H); ^13^C NMR (100.61 MHz, CDCl_3_) *δ* (signals for 4 diastereoisomers) 34.4 (CH_3_), 34.52 (CH_3_), 34.53 (CH_3_), 34.6 (CH_3_), 39.3 (CH), 40.1 (CH), 40.2 (CH), 42.3 (CH), 46.0 (CH), 46.5 (CH), 47.4 (CH), 48.1 (CH), 48.4 (CH), 48.5 (CH), 48.6 (CH), 49.6 (CH), 52.8 (CH_2_), 54.3 (CH_2_), 54.5 (CH_2_), 55.4 (CH_2_), 69.7 (CH_2_), 69.9 (CH_2_), 70.5 (CH_2_), 71.9 (CH_2_), 73.44 (CH_2_), 73.46 (CH_2_), 73.70 (CH_2_), 73.74 (CH_2_), 113.47 (CH), 113.54 (CH), 113.66 (CH), 113.73 (CH), 116.9 (CH_2_), 117.4 (CH_2_), 117.9 (CH_2_), 118.2 (CH_2_), 123.7 (CH), 123.82 (CH), 123.87 (CH), 123.9 (CH), 125.02 (CH), 125.05 (CH), 125.2 (CH), 125.6 (CH), 128.76 (CH), 128.84 (CH), 128.89 (CH), 128.95 (CH), 132.9 (C), 133.0 (C), 133.7 (C), 134.1 (C), 136.1 (CH), 136.2 (CH), 137.4 (CH), 138.1 (CH), 141.99 (C), 142.03 (C), 142.3 (C), 142.4 (C); *m/z* (CI) 311 [(M+NH_4_)^+^, 100%], 216 (21), 118 (14).

The melting points and spectroscopic data of octamethyloxamidinium bis(tetrafluoroborate) **21** and diphenyl disulfide (**33**) [7,10-12] were identical to those reported earlier in this experimental section and with those reported in the literature cited.

**Cascade radical cyclization of 2-{[4-(4-benzenesulfonylbut-2-enyloxy)but-2-enyl] methanesulfonylamino}benzenediazonium tetrafluoroborate (44) by TDAE**

Following an experimental procedure [General procedure D, Table 2, entry (vi)] identical to that described for the cyclization of 2-[{(*Z*)-4-benzenesulfinylbut-2-enyl}methanesulfonylamino]benzene­diazonium tetrafluoroborate (**31b**) by TDAE, *N*-(2-aminophenyl)-*N*-[4-(4-benzenesulfonylbut-2-enyloxy)but-2-enyl]methanesulfonamide (**43**, 0.1352 g, 0.3 mmol, 1.0 equiv) was first reacted with nitrosonium tetrafluoroborate (0.0421 g, 0.36 mmol, 1.2 equiv) in dichloromethane (10 ml) at −10 °C for 1.5 h under an argon atmosphere to obtain the diazonium salt **44** as a reddish brown semi-solid. The diazonium salt **44** was immediately dissolved in degassed acetone (2 ml) and reacted with TDAE (0.07 ml, 0.3 mmol, 1.0 equiv) at r.t. under an argon atmosphere for 10 min, to afford octamethyloxamidinium bis(tetrafluoroborate) (**21**, 0.026 g, 46%) as a pale white powder, mp 230 °C (dec) and a yellow semi-solid (after work-up), which upon purification by flash chromotography on silica gel afforded the expected bicyclised product, 1-methanesulfonyl-3-(4-vinyltetrahydrofuran-3-yl)-2,3-dihydro-1*H*-indole [7] (**47**, 0.068 g, 77% in two steps, from **43**) [eluant: ethyl acetate/petroleum ether = 3:7] as a yellow semi-solid. The bicylised product **47** was isolated as a mixture of 4 diastereoisomers in the ratio 1:4:1.5:3.5 as determined from its ^1^H NMR spectrum. The physical and spectroscopic data of octamethyloxamidinium bis(tetrafluoroborate) **21** and bicyclised product **47** [7] were identical to those reported earlier in this experimental section and with those reported in the literature cited.

**Experimental Section IV**

**Preparation of the amines 48b–d:**

The amine **48a** was prepared from *N*-(2-nitrophenyl)methanesulfonamide (**25a**) and amines **48b–d** were prepared following literature [3] procedures (Scheme 12).

**Scheme 12:** Preparation of the amines **48a–d.** *Reagents and conditions*: (i) *N*-(2-Nitrophenyl)methanesulfonamide (**25a**), NaH, anhydrous DMF, 0 °C to r.t., 1 h, 2,3-dibromopropene, reflux, 24 h, 89%; (ii) Cu(acac)_2_, NaBH_4_, EtOH, 25 °C, 2.5 h, 73%; (iii) Bromine, CH_2_Cl_2_, −10 °C to r.t., 2 h, then NEt_3_, 0.5 h, HCl (2 N), 61% (**81b**), 49% (**81c**); (iv) NaBH_4_, CeCl_3_·7H_2_O, CH_3_OH, 0 °C, 2 h, 64% (**82b**), 68% (**82c**); (v) *N*-(2-Nitrophenyl)methanesulfonamide **25a**, DIAD, PPh_3_, THF, 0 °C to r.t., 16 h, 56% (**79b**), 67% (**79c**); (vi) SnCl_2_·2H_2_O, CH_3_OH, reflux, 5 h, 86% (**48b**), 87% (**48c**); (vii) CHBr_3_, KO*t*-Bu, hexane, 0 °C, 81%; (viii) AgClO_4_, H_2_O, acetone, 0 °C, 82%; (ix) DEAD, Ph_2_MeP, THF, *N*-(2-nitrophenyl)methanesulfonamide (**25a**), 0 °C then reflux, 41%; (x) Cu(acac)_2_, NaBH_4_, EtOH, 0 °C to r.t., 74%.

***N*-(2-Bromoallyl)-*N*-(2-nitrophenyl)methanesulfonamide (79a)**

Sodium hydride (0.960 g of 60% NaH dispersed in mineral oil, 24.0 mmol, 1.2 equiv) was washed with dry hexane (2 × 10 ml) at 0 °C under nitrogen and suspended in anhydrous DMF (50 ml). To this suspension was added *N*-(2-nitrophenyl)methanesulfonamide (**25a**, 4.324 g, 20.0 mmol, 1.0 equiv) at 0 °C and evolution of hydrogen gas bubbles was observed. The mixture was brought to r.t. and stirred under nitrogen for 1 h followed by addition of 2,3-dibromopropene (5.996 g of 80% composition, 24.0 mmol, 1.2 equiv). The reaction mixture was heated to reflux under a nitrogen atmosphere for 24 h. When the reaction was complete, the reaction mixture was extracted with diethyl ether (250 ml) and washed with brine solution (4 × 150 ml) and water (4 × 150 ml). The organic layer was separated, dried over anhydrous magnesium sulfate and evaporated to dryness to obtain a yellow semi-solid which upon purification by flash chromatography on silica gel (eluant: ethyl acetate/petroleum ether = 3:7) afforded the *N*-(2-bromoallyl)-*N*-(2-nitrophenyl)methanesulfonamide [7] (**79a**, 5.931 g, 89%) as a yellow semi-solid [Found: MNH_4_^+^ (ESI), 351.9961. C_10_H_11_N_2_O_4_S^79^Br requires *MNH_4_*, 351.9967]; (Found: C, 35.69; H, 3.36; N, 8.34; Br, 23.54; C_10_H_11_N_2_O_4_SBr requires C, 35.83; H, 3.31; N, 8.36; Br, 23.84%); IR (neat film, NaCl) 3106, 3036, 2935, 1629, 1603, 1581, 1533, 1483, 1350, 1231, 1155, 1098, 1078, 1010, 960, 875, 845, 784 cm^−1^; ^1^H NMR (400.13 MHz, CDCl_3_) *δ* 3.11 (s, 3H), 4.59 (broad s, 2H), 5.59 (d, *J* = 2.0 Hz, 1H), 5.75–5.80 (m, 1H), 7.52–7.61 (m, 1H), 7.64–7.72 (m, 2H), 7.98 (dd, *J* = 6.9, 1.4 Hz, 1H); ^13^C NMR (100.61 MHz, CDCl_3_) *δ* 41.8 (CH_3_), 60.3 (CH_2_), 123.5 (CH_2_), 126.3 (CH), 128.2 (C), 130.6 (CH), 132.1 (C), 134.3 (CH), 134.6 (CH), 148.9 (C); *m/z* (CI) 354 [{M (^81^Br) + NH_4_}^+^, 100%], 352 [{M (^79^Br) + NH_4_}^+^, 99%], 305 (79), 303 (79), 223 (46), 225 (58), 39 (147), 145 (32).

***N*-(2-Aminophenyl)-*N*-(2-bromoallyl)methanesulfonamide (48a)**

Following an experimental procedure identical to that described for the preparation of *N*-(2-aminophenyl)-*N*-but-2-enylmethanesulfonamide (**78**), copper(II) acetylacetonate (0.576 g, 2.2 mmol, 0.2 equiv) was first reacted with sodium borohydride (0.416 g, 11.0 mmol, 1.0 equiv) in ethanol (300 ml) at r.t. for 1 h under nitrogen to form black flakes in clear solution. This catalyst was then reacted with *N*-(2-bromoallyl)-*N*-(2-nitrophenyl)methanesulfonamide (**79a**, 3.687 g, 11.0 mmol, 1.0 equiv) and sodium borohydride (0.832 g, 22.0 mmol, 2.0 equiv) in ethanol (580 ml) for 2.5 h at r.t. under a nitrogen atmosphere to afford *N*-(2-aminophenyl)-*N*-(2-bromoallyl)methanesulfonamide [7] (**48a**, 2.455 g, 73%) [purification by flash chromatography on silica gel; eluant: hexane/ethyl acetate = 7:3] as a pale yellow solid; mp 106–106.5 °C [Found: MH^+^ (ESI), 304.9952. C_10_H_13_N_2_O_2_S^79^Br requires *MH*, 304.9954]; IR (KBr disc) 3484, 3387, 3035, 3014, 2993, 2920, 2904, 1619, 1500, 1455, 1434, 1416, 1384, 1331, 1265, 1213, 1160, 1084, 1063, 1037, 1007, 962, 914, 875, 855, 827, 765, 747 cm^−1^; ^1^H NMR (400.13 MHz, CDCl_3_) *δ* 3.21 (s, 3H), 4.27 (broad s, 2H), 4.55 (broad s, 2H), 5.60 (d, *J* = 2.1 Hz, 1H), 5.77–5.81 (m, 1H), 6.83 (ddd, *J* = 7.7, 7.6, 1.3 Hz, 1H), 6.88 (dd, *J* = 8.0, 1.3 Hz, 1H), 7.21–7.29 (m, 2H); ^13^C NMR (100.61 MHz, CDCl_3_) *δ* 39.7 (CH_3_), 58.8 (CH_2_), 118.0 (CH), 119.2 (CH), 122.4 (CH_2_), 124.2 (C), 128.4 (C), 130.1 (CH), 130.7 (CH), 146.8 (C); *m/z* (EI) 306 [{M (^81^Br)}^+•^, 5%], 304 [{M (^79^Br)}^+•^, 5%], 227 (52), 225 (53), 209 (10), 207 (10), 145 (100), 129 (20), 119 (52), 104 (19), 92 (15), 91 (11), 79 (53), 65 (32), 52 (19).

**Table 3:** Initial optimization studies for cyclization of arenediazonium salt **49a.**

| **Entry** | **Diazonium Salt** | **Equivalents of TDAE** | **Solvent** | **Temperature**  **(°C)** | **Time** | **Isolated Yield**^a^ **(%) of** | |
| --- | --- | --- | --- | --- | --- | --- | --- |
|  |  |  |  |  |  | **51a** | **52a** |
| (i) | **49a** | 1.0 | Acetone | 25 | 30 min | 39^b^ | 40 |
| (ii) | **49a** | 1.5 | DMF (anhydrous) | 25 | 10 min | 68^c^ | 0 |

^a^All isolated yields were calculated on the basis of the quantity of the starting aryl amine **48a.** ^b^The product **51a** was isolated directly from the reaction mixture after auto-tautomerisation of **50a** to indole during storage of reaction mixture prior to purification by flash chromatography. ^c^The product **51a** was obtained by treatment of the intermediate exocyclic alkene **50a** with *p*-toluenesulfonic acid monohydrate in dichloromethane at r.t. for 12 h.

**Representative Procedure [entry (ii)]:** *N*-(2-Aminophenyl)-*N*-(2-bromoallyl)methanesulfonamide (**48a**, 0.1526 g, 0.5 mmol, 1.0 equiv) was dissolved in dry and degassed dichloromethane (5 ml) and the solution was cooled to −10 °C under an argon atmosphere. Nitrosonium tetrafluoroborate (0.073 g, 0.625 mmol, 1.25 equiv) was added to the solution and the reaction mixture was stirred under an argon atmosphere at −10 °C for 1.5 h. When the reaction was judged to be complete by TLC analysis, the reaction mixture was warmed to 0 °C and dichloromethane was removed from the reaction mixture under reduced pressure to afford the 2-[(2-bromoallyl)methanesulfonylamino]benzenediazonium tetrafluoroborate (**49a**) [7] as a reddish brown sticky semi-solid. This was immediately dissolved in degassed anhydrous DMF (5 ml, degassed by purging with a stream of argon gas for 30 min at r.t.) and was treated with TDAE (0.18 ml, 0.75 mmol, 1.5 equiv) at r.t. under an argon atmosphere. The reaction mixture turned deep red immediately with effervescence of nitrogen bubbles and the mixture was stirred under argon at r.t. After 10 min, the reaction mixture turned reddish orange turbid solution and the reaction was complete. The mixture was then extracted with diethyl ether (2 × 50 ml) and washed sequentially with saturated brine solution (5 × 50 ml) and water (3 × 50 ml). The organic phase was separated, dried over anhydrous sodium sulfate, evaporated to dryness to obtain a brown semi-solid which was purifed by flash chromatography on silica gel (eluant: ethyl acetate/petroleum ether = 3:17) to afford 1-methanesulfonyl-3-methylene-2,3-dihydro-1*H*-indole [7,14] (**50a**, 0.076 g, 73% from **48a**) as a colourless solid. The indoline **50a** (0.076 g) was immediately dissolved in dichloromethane (5 ml) and *p*-toluenesulfonic acid monohydrate (0.114 g, 0.6 mmol, 1.64 equiv) was added to the solution. The mixture was stirred under an argon atmosphere for 12 h at r.t. The reaction mixture was extracted with diethyl ether (50 ml) and washed with saturated sodium bicarbonate solution (2 × 50 ml) and water (1 × 50 ml). The ethereal layer was separated, dried over anhydrous sodium sulfate, filtered and concentrated *in vacuo* to afford a pale white semi-solid which upon purification by flash chromatography on silica gel (ethyl acetate/petroleum ether = 3:17) provided 1-methanesulfonyl-3-methyl-1*H*-indole [15] (**51a**, 0.071 g, 68% in three steps, from **48a**) as a white solid.

**1-Methylsulfonyl-3-methyleneindoline (50a)** [7,14]: mp 113–115 °C (lit. [14] mp 114–116 °C); [Found: (M+NH_4_)^+^ (ESI), 227.0851. C_10_H_11_NO_2_S requires *MNH_4_*, 227.0854]; IR (KBr disc) 3115, 3020, 2930, 1606, 1477, 1448, 1360, 1277, 1208, 1171, 1122, 1003, 979 cm^−1^; ^1^H NMR (400.13 MHz, CDCl_3_) *δ* 2.88 (s, 3H), 4.61–4.63 (m, 2H), 5.12 (t, *J* = 3.0 Hz, 1H), 5.53 (t, *J* = 3.0 Hz, 1H), 7.09 (ddd, *J* = 8.4, 7.0, 0.9 Hz, 1H), 7.28 (ddd, *J* = 8.4, 7.0, 1.1 Hz, 1H), 7.47–7.51 (m, 2H); ^13^C NMR (100.61 MHz, CDCl_3_) *δ* 35.3 (CH_3_), 55.4 (CH_2_), 103.0 (CH_2_), 114.6 (CH), 119.8 (CH), 121.4 (CH), 124.1 (CH), 129.8 (C), 140.2 (C), 144.5 (C); *m/z* (CI) 227 [(M+NH_4_)^+^, 100%], 210 (28), 146 (25), 132 (77), 130 (97), 118(15).

**1-Methanesulfonyl-3-methyl-1*H*-indole (51a)** [15]: mp 49–50 °C [Found: MNH_4_^+^ (ESI), 227.0850. C_10_H_11_NO_2_S requires *MNH_4_*, 227.0849]; IR (KBr disc) 3119, 3065, 3009, 2920, 2859, 1609, 1587, 1452, 1351, 1276, 1205, 1164, 1117, 1004, 979, 797, 785, 759 cm^−1^; ^1^H NMR (400.13 MHz, CDCl_3_) *δ* 2.36 (d, *J* = 1.3 Hz, 3H), 3.09 (s, 3H), 7.27 (m, 1H), 7.39 (ddd, *J* = 7.6, 7.3, 1.4 Hz, 1H), 7.44 (ddd, *J* = 7.8, 7.3, 1.6 Hz, 1H), 7.60–7.65 (m, 1H), 7.94–8.00 (m, 1H); ^13^C NMR (100.61 MHz, CDCl_3_) *δ* 10.1 (CH_3_), 40.7 (CH_3_), 113.7 (CH), 119.1 (C), 120.2 (CH), 123.4 (CH), 123.8 (CH), 125.4 (CH), 132.3 (C), 135.9 (C); *m/z* (CI) 227 [(M+NH_4_)^+^, 100%], 210 (8), 209 (14), 130 (20), 52 (19).

**(1-Methanesulfonyl-1*H*-indol-3-yl)methanol (52a)** [7,14]: Colourless solid (eluant: ethyl acetate/petroleum ether = 1:1); mp 117–119°C (lit. [14] mp 117–120°C) [Found: MNH_4_^+^ (ESI), 243.0801. C_10_H_11_NO_3_S requires *MNH_4_*, 243.0803]; IR (KBr disc) 3407, 3056, 2992, 1454, 1366, 1267, 1173, 1128 cm^−1^; ^1^H NMR (400.13 MHz, CDCl_3_) *δ* 1.66 (s, 1H), 3.11 (s, 3H), 4.89 (d, *J* = 0.9 Hz, 2H), 7.35 (ddd, *J* = 7.8, 7.3, 1.0 Hz, 1H), 7.41 (ddd, *J* = 8.3, 7.3, 1.2 Hz, 1H), 7.46 (s, 1H), 7.68–7.75 (m, 1H), 7.90–7.95 (m, 1H); ^13^C NMR (100.61 MHz, CDCl_3_) *δ* 41.2 (CH_3_), 57.6 (CH_2_), 113.7 (CH), 120.7 (CH), 122.7 (C), 124.1 (CH), 124.2 (CH), 125.8 (CH), 129.9 (C), 136.0 (C); *m/z* (EI) 225 (M^+•^, 56%), 146 (30), 118 (100), 91(55).

**Cyclization of the Arenediazonium Salts 49b–d by TDAE**

**4-Methanesulfonyl-1,2,3,4-tetrahydrocyclopenta[*b*]indole (51b):** The indole **51b** [3,7,16] was obtained as white needles (0.089 g, 63% from **48b**), in three steps from the amine [3] **48b** (0.1987 g, 0.6 mmol) following the procedure identical to that described for the cyclization of the arenediazonium salt **49a** by TDAE; (purification by flash chromatography on silica gel; eluant: ethyl acetate/petroleum ether = 1:9); mp 135–136 °C (lit. mp [3] 136–137 °C); (Found: C, 61.35; H, 5.54; N, 5.84. C_12_H_13_NO_2_S requires C, 61.25; H, 5.57; N, 5.95%); IR (KBr disc) 3023, 2964, 2916, 2863, 1612, 1477, 1448, 1401, 1362, 1343, 1324, 1248, 1163, 1114, 1001, 963, 831, 772, 741 cm^−1^; ^1^H NMR (400.13 MHz, CDCl_3_) *δ* 2.48–2.62 (m, 2H), 2.75–2.85 (m, 2H), 3.03 (s, 3H), 3.04–3.12 (m, 2H), 7.22–7.31 (m, 2H), 7.38–7.49 (m, 1H), 7.85–7.97 (m, 1H); ^13^C NMR (100.61 MHz, CDCl_3_) *δ* 24.6 (CH_2_), 28.0 (CH_2_), 28.2 (CH_2_), 41.0 (CH_3_), 114.5 (CH), 119.8 (CH), 124.0 (CH), 124.1 (CH), 127.0 (C), 127.8 (C), 140.9 (C), 144.3 (C); *m/z* (EI) 235 (M^+•^, 46%), 156 (100), 128 (25).

**9-Methanesulfonyl-1,2,3,4-tetrahydro-9*H*-carbazole 51c:** The indole **51c** [3,7,16] was obtained as an off-white solid (0.031 g, 43% from **48c**), in three steps starting from *N***-**(2**-**aminophenyl)-*N***-**(2-bromocyclohex-2-enyl)methanesulfonamide [3] (**48c**, 0.1001 g, 0.29 mmol) following the procedure identical to that described for the cyclization of the arenediazonium salt **49a** by TDAE; (purification by flash chromatography on silica gel; eluant: ethyl acetate/petroleum ether = 1:9); mp 103–104 °C (lit. [3] mp 104–105 °C); [Found: M^+•^ (EI), 249.0815. C_13_H_15_NO_2_S requires 249.0818]; (Found: C, 62.53; H, 5.86; N, 5.29. C_13_H_15_NO_2_S requires C, 62.62; H, 6.06; N, 5.62%); IR (KBr disc) 3031, 2931, 2842, 1612, 1453, 1361, 1223, 1174, 1162, 1142, 1023, 999, 985, 960, 786, 759, 744 cm^−1^; ^1^H NMR (400.13 MHz, CDCl_3_) *δ* 1.84–1.89 (m, 2H), 1.90–1.98 (m, 2H), 2.64–2.71 (m, 2H), 2.91–2.97 (m, 2H), 2.98 (s, 3H), 7.27–7.32 (m, 2H), 7.40–7.47 (m, 1H), 7.96–8.03 (m, 1H); ^13^C NMR (100.61 MHz, CDCl_3_) *δ* 21.4 (CH_2_), 22.3 (CH_2_), 23.5 (CH_2_), 24.7 (CH_2_), 40.5 (CH_3_), 114.1 (CH), 118.4 (CH), 118.8 (C), 123.7 (CH), 124.3 (CH), 130.6 (C), 135.7 (C), 136.3 (C); *m/z* (EI) 249 (M^+•^, 63%), 170 (100).

**5-Methanesulfonyl-5,6,7,8,9,10,11,12-octahydrocyclonona[*b*]indole (51d):** The indole **51d** [3,16] was obtained as an off-white solid (0.024 g, 64% from **48d**), in three steps starting from *N***-**(2**-**aminophenyl)-*N***-**(2-bromocyclonon-2-enyl)methanesulfonamide [3] (**48d**, 050 g, 0.129 mmol) following the procedure identical to that described for the cyclization of the arenediazonium salt **49a** by TDAE; (purification by flash chromatography on silica gel; eluant: ethyl acetate/petroleum ether = 1:9); mp 99–100 °C (lit. [3] mp 101–102 °C); [Found: M^+•^ (EI), 291.1293. C_16_H_21_NO_2_S requires 291.1288]; IR (KBr disc) 3014, 2928, 2857, 1474, 1456, 1362, 1225, 1169, 1151, 1121, 987, 961, 768, 746 cm^−1^; ^1^H NMR (400.13 MHz, CDCl_3_) *δ* 1.25–1.31 (m, 2H), 1.40–1.55 (m, 4H), 1.67–1.77 (m, 2H), 1.78–1.87 (m, 2H), 2.77–2.83 (m, 2H), 2.95 (s, 3H), 3.13 (t, *J* = 6.2 Hz, 2H), 7.27–7.33 (m, 2H), 7.46–7.51 (m, 1H), 8.01–8.06 (m, 1H); ^13^C NMR (100.61 MHz, CDCl_3_) *δ* 23.2 (CH_2_), 24.3 (CH_2_), 24.6 (CH_2_), 25.8 (CH_2_), 25.9 (CH_2_), 26.7 (CH_2_), 27.4 (CH_2_), 40.5 (CH_3_), 115.0 (CH), 119.0 (CH), 122.7 (C), 124.0 (CH), 124.7 (CH), 131.0 (C), 137.1 (C), 137.6 (C); *m/z* (EI) 291 (M^+•^, 64%), 212 (100).

**Experimental Section V**

1-(2-Nitrobenzenesulfonyl)indoline (60)

Indoline (**59**, 2.81 ml, 25.0 mmol, 1.0 equiv) was added to a solution of 4-(dimethylamino)pyridine (0.305 g, 27.5 mmol, 1.1 equiv), triethylamine (3.49 ml, 25.0 mmol, 1.0 equiv) in pyridine (100 ml) under nitrogen at 0 °C. To this solution was added 2-nitrobenzenesulfonyl chloride (6.094 g, 27.5 mmol, 1.1 equiv) dissolved in pyridine (25 ml) dropwise at 0 °C. The reaction mixture was stirred for 0.5 h at 0 °C and then warmed to r.t. The mixture was then heated under reflux at 110 °C for 27 h. After cooling, the mixture was poured into concentrated hydrochloric acid. The aqueous phase was then extracted with dichloromethane (3 × 75 ml) and the combined organic phases were washed with saturated sodium bicarbonate solution (3 × 75 ml) and then with saturated brine solution (3 × 75 ml). The organic extract was then dried over anhydrous magnesium sulfate. The solvent was then removed under reduced pressure to give a dark brown solid. This was then subjected to column chromatography over silica using toluene as the eluant to afford 1-(2-nitrobenzene)sulfonylindoline (**60**) as a pale yellow solid (4.320 g, 57%); mp 107–108 °C [Found: M^+•^ (EI), 304.0519. C_14_H_12_N_2_SO_4_ requires *M*, 304.0513]; IR (KBr disc) 3102, 3033, 2937, 1542, 1483, 1462, 1330, 1243, 1165, 851, 783, 746 cm^−1^; ^1^H NMR (400.13 MHz, CDCl_3_) *δ* 3.16 (t, *J* = 8.4 Hz, 2H), 4.24 (t, *J* = 8.4 Hz, 2H), 7.11 (ddd, *J* = 8.1, 7.4, 0.8 Hz, 1H), 7.24–7.31 (m, 2H), 7.48–7.60 (m, 1H), 7.65–7.72 (m, 2H), 7.77 (ddd, *J* = 8.1, 7.2, 1.3 Hz, 1H), 8.03 (dd, *J* = 8.0, 1.0 Hz, 1H); ^13^C NMR (100.61 MHz, CDCl_3_) *δ* 28.3 (CH_2_), 50.8 (CH_2_), 114.8 (CH), 124.60 (CH), 124.64 (CH), 125.9 (CH), 128.1 (CH), 130.3 (CH), 131.7 (C), 132.1 (C), 132.2 (CH), 134.6 (CH), 141.4 (C), 148.7 (C); *m/z* (EI) 304 (M^+•^, 73%), 149 (7), 118 (100), 91 (92), 84 (46), 65 (25), 50 (23).

**1-(2-Aminobenzenesulfonyl)indoline (61)**

1-(2-Nitrobenzenesulfonyl)indoline (**60**, 3.043 g, 10.0 mmol, 1.0 equiv) was dissolved in ethyl acetate (100 ml) under a nitrogen atmosphere. The solution was cooled to 0 °C and palladium on activated carbon (10% Pd, 0.750 g) was added in small portions and the solution was stirred well. The solution was then brought to r.t. and stirred vigorously under a pressure of hydrogen gas (balloon) for a period of 3 h. The progress of the reaction was constantly monitored by TLC. After 3 h, when the reaction was judged to be complete by TLC, the reaction mixture was filtered through Celite® 521 and evaporated at reduced pressure to get a white solid which was purified by column chromatography on silica [eluant: petroleum ether/diethyl ether = 1:1] to obtain 1-(2-aminobenzene)sulfonylindoline (**61**) as fine white needles (2.675 g, 98%); mp 113 °C [Found: M^+•^ (EI), 274.0775. C_14_H_14_N_2_SO_2_ requires *M*, 274.0771]; IR (KBr disc) 3468, 3371, 1619, 1481, 1337, 1323, 1239, 1159, 1055, 974, 761, 752 cm^−1^; ^1^H NMR (400.13 MHz, CDCl_3_) *δ* 2.97 (t, *J* = 8.4 Hz, 2H), 4.08 (t, *J* = 8.4 Hz, 2H), 5.10 (s, 2H), 6.65–6.71 (m, 2H), 7.01 (ddd, *J* = 7.4, 6.4, 0.9 Hz, 1H), 7.09–7.15 (m, 1H), 7.17–7.22 (m, 1H), 7.25–7.31 (m, 1H), 7.52–7.67 (m, 2H); ^13^C NMR (100.61 MHz, CDCl_3_) *δ* 28.6 (CH_2_), 50.5 (CH_2_), 115.7 (CH), 117.8 (CH), 118.2 (CH), 119.9 (C), 124.2 (CH), 125.6 (CH), 128.1 (CH), 130.3 (CH), 132.6 (C), 134.9 (CH), 142.8 (C), 146.9 (C); *m/z* (EI) 274 (M^+•^, 66%), 118 (100), 91 (90), 65 (52), 51 (8).

**2-(2,3-Dihydroindole-1-sulfonyl)benzenediazonium tetrafluoroborate (62)**

A solution of 1-(2-aminobenzenesulfonyl)indoline (**61**, 0.823 g, 3.0 mmol, 1.0 equiv) in dry dichloromethane (15 ml) was added to a stirred suspension of nitrosonium tetrafluoroborate (0.526 g, 4.5 mmol, 1.5 equiv) in dry dichloromethane (35 ml) under nitrogen at −10 °C. After 1 h, further nitrosonium tetrafluoroborate (0.175 g, 1.5 mmol, 0.5 equiv) was added to the reaction mixture and after 0.5 h the dichloromethane was removed under reduced pressure. The residue was dissolved in little acetone (2 ml) and recrystallised from cold diethyl ether between −10 °C and 0 °C, then filtered under nitrogen to give 2-(2,3-dihydroindole-1-sulfonyl)benzenediazonium tetrafluoroborate (**62**) as a red solid which immediately turned into a red semi-solid on exposure to atmosphere (1.062 g, 95%); ^1^H NMR (400.13 MHz, DMSO-*d*_6_) 3.00 (t, *J* = 8.2 Hz, 2H), 4.22 (t, *J* = 8.2 Hz, 2H), 7.10–7.21 (m, 1H), 7.22–7.37 (m, 2H), 7.57 (d, *J* = 8.1 Hz, 1H), 8.21 (dd, *J* = 7.9, 1.2 Hz, 1H), 8.27 (ddd, *J* = 8.0, 7.9, 1.2 Hz, 1H), 8.36 (ddd, *J* = 7.8, 7.7, 1.3 Hz, 1H), 9.08 (dd, *J* = 8.1, 1.3 Hz, 1H); ^13^C NMR (100.61 MHz, DMSO-*d*_6_) 28.3 (CH_2_), 51.4 (CH_2_), 115.3 (C), 115.5 (CH), 126.4 (CH), 127.0 (CH), 129.0 (CH), 131.3 (CH), 133.8 (C), 136.8 (CH), 137.5 (CH), 137.7 (C), 140.5 (C), 142.8 (CH).

**Cyclization of arenediazonium salt 62 by TDAE**

The diazonium salt **62** (0.125 g, 0.335 mmol, 1.0 equiv) was dissolved in degassed acetone (10 ml) and methanol (10 ml) under a nitrogen atmosphere and to this, TDAE (0.671 g, 0.335 mmol, 1.0 equiv) was added. Rapid evolution of nitrogen gas was observed and the solution turned deep-red to orange. The reaction was stirred vigorously for 10 min under a nitrogen atmosphere at r.t. and slowly the solution decolourised to pale red-orange turbid solution. The reaction mixture was evaporated to dryness under reduced pressure to obtain a red semi-solid which was dissolved in a mixture of ethyl acetate (50 ml) and water (100 ml). The layers were separated and the aqueous phase was extracted with ethyl acetate (50 ml). The combined organic layers were washed with deionised water (3 × 100 ml), dried over anhydrous magnesium sulfate, filtered and evaporated to dryness to give a brown solid, which was chromatographed [eluant: ethyl acetate/petroleum ether = 1:9] on silica gel to yield indole [17] (**63**, 0.013 g, 33%) along with 1-(benzenesulfonyl)indole (**64**) [18-20] as a brown solid (0.004 g, 5%) and 4,5-dihydro-6-thia-5a-aza-acephenanthrylene-6,6-dioxide (**65**) as a brown solid (0.052 g, 60%).

**Indole (63):** mp 48–49.5 °C (lit. [17] mp 49.5–50.5 °C) [Found: MH^+^ (ESI), 118.0653. C_8_H_7_N requires *MH*, 118.0651]; IR (CHCl_3_) 3480, 3423, 3011, 1511, 1456, 1413, 1353, 1334, 1277, 1246, 1225, 1206, 1091, 1065, 929, 792, 723 cm^−1^; ^1^H NMR (400.13 MHz, CDCl_3_) 6.68 (t, *J* = 2.1 Hz, 1H), 7.23–7.25 (m, 1H), 7.26–7.30 (m, 1H), 7.32 (ddd, *J* = 8.1, 8.0, 1.0 Hz, 1H), 7.48 (d, *J* = 8.0 Hz, 1H), 7.78 (d, *J* = 7.8 Hz, 1H), 8.12 (broad s, 1H); ^13^C NMR (100.61 MHz, CDCl_3_) 102.8 (CH), 111.2 (CH), 120.0 (CH), 120.9 (CH), 122.2 (CH), 124.3 (CH), 128.1 (C), 136.0 (C); *m/z* (CI) 118 [(M+H)^+^, 100%]. The spectroscopic data of the indole **63** were consistent with those of an authentic commercial sample.

**1-(Benzenesulfonyl)indole (64)** [18-20]: mp 71–73 °C (lit. [19] mp 76–78 °C) [Found: M^+•^ (EI), 257.0509. C_14_H_11_NSO_2_ requires *M*, 257.0506]; IR (KBr disc) 3143, 3124, 3005, 1529, 1447, 1372, 1262, 1189, 1175, 1090, 989, 880, 765, 727 cm^−1^; ^1^H NMR (400.13 MHz, CDCl_3_) 6.71 (d, *J* = 3.7 Hz, 1H), 7.24–7.30 (m, 1H), 7.35 (ddd, *J* = 8.4, 8.3, 1.1 Hz, 1H), 7.44–7.49 (m, 2H), 7.52–7.59 (m, 2H), 7.59 (d, *J* = 3.7 Hz, 1H), 7.90–7.95 (m, 2H), 8.05 (d, *J* = 8.3 Hz, 1H); ^13^C NMR (100.61 MHz, CDCl_3_) 109.8 (CH), 114.0 (CH), 121.9 (CH), 123.9 (CH), 125.2 (CH), 126.8 (CH), 127.2 (CH), 129.8 (CH), 131.3 (C), 134.3 (CH), 135.4 (C), 138.8 (C); *m/z* (EI) 257 (M^+•^, 100%), 141 (25), 116 (99), 85 (61), 84 (96), 77 (53), 51 (44).

**4,5-Dihydro-6-thia-5a-aza-acephenanthrylene-6,6-dioxide (65):** mp 135-137 °C [Found: M^+•^ (EI), 257.0513, C_14_H_11_NSO_2_ requires *M*, 257.0506]; IR (KBr disc) 3062, 2995, 1593, 1467, 1424, 1315, 1170, 1134, 1101, 1029, 954, 761 cm^−1^; ^1^H NMR (400.13 MHz, CDCl_3_) 3.34 (t, *J* = 8.4 Hz, 2H), 4.28 (t, *J* = 8.4 Hz, 2H), 7.13–7.19 (m, 1H), 7.26–7.30 (m, 1H), 7.51–7.59 (m, 1H), 7.65–7.77 (m, 2H), 7.97 (d, *J* = 8.0 Hz, 1H), 8.12 (d, *J* = 8.0 Hz, 1H); ^13^C NMR (100.61 MHz, CDCl_3_) 28.8 (CH_2_), 45.2 (CH_2_), 118.8 (C), 122.3 (CH), 124.1 (CH), 124.5 (2 × CH), 126.4 (CH), 128.8 (CH), 131.6 (C), 132.9 (C), 133.3 (CH), 133.6 (C), 142.7 (C); *m/z* (EI) 257 (M^+•^, 100%), 192 (70), 165 (13), 96 (13), 84 (31), 51 (13). The structure of the compound was further characterised by 2D [1H, 1H] COSY NMR and 1D NOE experiments.

**Structure elucidation of the tetracyclic sulfonamide 65 by NMR studies**

The tetracyclic sulfonamide **65** formed as the major product during the course of the reaction of the arenediazonium salt **62** with TDAE involved the formation of the aryl-aryl C-C bond and this was confirmed by performing a series of NMR experiments on **65** (Figure 1).

The aromatic region in the 1D ^1^H-NMR spectrum of **65** consisted of seven signals, A–G indicated in the Figure 1-(ii). Four of these signals, A, B, C and F were large doublets indicating that the protons giving rise to each of these signals would have a single aromatic proton as neighbour. (Note: The apparent appearance of F being triplet is due to the residual proton signal from the solvent, CDCl_3_, resonates at δ 7.25 ppm and appears next to the doublet signal F). The aromatic region also showed three triplet signals, D, E, G. Thus the correct number and type of resonances were present in the 1D ^1^H-NMR spectrum of the molecule **65** to work out the basic skeleton of the molecule **65**, in which two aromatic rings I and II were joined by a covalent linkage as shown in the Figure 1 (vii). We wished to confirm this supposition. The confirmation of the covalent linkage between the two aromatic rings would come from the observation of a NOE between aromatic proton resonances on different aromatic rings. Thus the molecule became a candidate for a NOE experiment.

**(vii) Four possible signal assignments for sulfonamide 65**

**(vi) Irradiating at F**

**(v) Irradiating at C**

**(iv) Irradiating at B**

**(iii) Irradiating at A**

**(ii) 1D ^1^H NMR Spectrum**

**(i) 2D [^1^H, ^1^H] COSY NMR Spectrum**

**Figure 1:** (i) 2D [1H, 1H] COSY NMR spectrum for the aromatic region of **65**; (ii) 1D ^1^H NMR spectrum for the aromatic region of sulfonamide **65**; (iii)-(vi) NOE NMR data for the aromatic region of sulfonamide **65**; (vii) Four possible signal assignments for the molecule **65**.

The key piece of NOE information that we were looking for was the exact locations of the protons which give rise to doublet signals. Hence it was necessary to work out which of the 4 doublets in the 1D ^1^H-NMR spectrum arose from the 7 aromatic protons of the structure **65**.

The logical next step was to link together the resonances that belonged to protons in the same ring. The quickest and easiest way of achieving this piece of information was to acquire a 2D [1H, 1H] COSY NMR data on the molecule **65** [see Figure 1 (i)]. The protons in an aromatic ring separated by three bonds would show large couplings to one another (^3^*J*_HH_ = 8–12 Hz) and therefore show strong correlations in ^1^H homonuclear COSY NMR data sets. The COSY NMR data clearly highlighted the presence of two distinct spin systems – **CGF** (Ring I) and **AEDB** (Ring II). However, from the COSY data alone it was not possible to distinguish between four possible assignment scenarios **65(i)–(iv)** shown in Figure 1(vii).

It becomes important to know whether an NOE occurs between any of the following pairs of resonances: (i) **F**–**B**, (ii) **C–A**, (iii) **F–A** or (iv) **B–C**. This would give us the key information both for assignment and the solution to the total puzzle. If an NOE does occur, then only one of the four possibilities of **65 (i)–(iv)** shown in the Figure 1(vii) will be satisfied. On the other hand, if no NOE was observed between any of the four possible resonances, then the molecule was not what was expected.

Irradiating at signal **A** [Figure 1 (iii)] resulted in an enhancement of the triplet **E** signal. From the information obtained from COSY experiment it was already known that **A** and **E** were neighbours in the ring (I) and so NOE confirms this. Similarly NOE confirms that protons **F** and **G** are neighbours by showing an enhancement of the triplet signal **G** [Figure 1 (vi)] when the signal **F** was irradiated.

Irradiating at the signal **B** [Figure 1 (iv)] results in an enhancement of both the doublet **C** and the triplet **D**. Further, on carrying out the NOE in reverse, *i.e.*, by irradiating at the doublet **C** resulted in enhancement of the doublet **B** and also additionally resulted in the enhancement of the triplet at **G** [Figure 1 (v)]. These two pieces of information provided the key to the total puzzle. Among the four different arrangements of atoms and assignments shown in the Figure 1(vii), the structure **65 (iv)** would satisfy all the NOE results and proved to be the expected correct structure of the sulfonamide **65**.

The structure of the sulfonamide **65** was also later confirmed by performing normal ^13^C decoupled, DEPT-135 and DEPT-90 NMR experiments.

**References for Experimental Section**

1. Weingarten, H.; White, W. A. *J. Org. Chem.* **1966,** *31,* 3427. doi:[10.1021/jo01348a520](http://dx.doi.org/10.1021/jo01348a520)
2. Fletcher, R.; Kizil, M.; Lampard, C.; Murphy, J. A.; Roome, S. J. *J. Chem. Soc., Perkin Trans. 1* **1998,** 2341. doi:[10.1039/a802974a](http://dx.doi.org/10.1039/a802974a)
3. Murphy, J. A.; Scott, K. A.; Sinclair, R. S.; Martin, C. G.; Kennedy, A. R.; Lewis, N. *J. Chem. Soc., Perkin Trans. 1* **2000,** 2395. doi:[10.1039/b002565h](http://dx.doi.org/10.1039/b002565h)
4. Reverdin, F. *Helv. Chim. Acta* **1929,** *12,* 1053. doi:[10.1002/hlca.192901201116](http://dx.doi.org/10.1002/hlca.192901201116)
5. Murphy, J. A.; Khan, T. A.; Zhou, S.-z.; Thomson, D. W.; Mahesh, M. *Angew. Chem., Int. Ed.* **2005,** *44,* 1356. doi:[10.1002/anie.200462038](http://dx.doi.org/10.1002/anie.200462038)
6. Khan, T. A.; Tripoli, R.; Crawford, J. J.; Martin, C. G.; Murphy, J. A. *Org. Lett.* **2003,** *5,* 2971. doi:[10.1021/ol035173i](http://dx.doi.org/10.1021/ol035173i)
7. LeStrat, F.; Murphy, J. A.; Hughes, M. *Org. Lett.* **2002,** *4,* 2735. doi:[10.1021/ol0262643](http://dx.doi.org/10.1021/ol0262643)
8. Roush, W. R.; Straub, J. A.; VanNieuwenhze, M. S. *J. Org. Chem.* **1991,** *56,* 1636. doi:[10.1021/jo00004a053](http://dx.doi.org/10.1021/jo00004a053)
9. Corlay, H.; Motherwell, W. B.; Pennell, A. M. K.; Shipman, M.; Slawin, A. M. Z.; Williams, D. J. *Tetrahedron* **1996,** *52,* 4883. doi:[10.1016/0040-4020(96)00160-3](http://dx.doi.org/10.1016/0040-4020(96)00160-3)
10. Pasto, D. J.; Smorada, R. L.; Turini, B. L.; Wampfler, D. J. *J. Org. Chem.* **1976,** *41,* 432. doi:[10.1021/jo00865a004](http://dx.doi.org/10.1021/jo00865a004)
11. Tanaka, H.; Ogawa, H.; Suga, H.; Torii, S.; Jutand, A.; Aziz, S.; Suarez, A. G.; Amatore, C. *J. Org. Chem.* **1996,** *61,* 9402. doi:[10.1021/jo960741b](http://dx.doi.org/10.1021/jo960741b)
12. Iranpoor, N.; Firouzabadi, H.; Pourali, A.-R. *Tetrahedron* **2002,** *58,* 5179. doi:[10.1016/S0040-4020(02)00390-3](http://dx.doi.org/10.1016/S0040-4020(02)00390-3)
13. Lemieux, R. M.; Devine, P. N.; Mechelke, M. F.; Meyers, A. I. *J. Org. Chem.* **1999,** *64,* 3585. doi:[10.1021/jo982426q](http://dx.doi.org/10.1021/jo982426q)
14. Tanaka, H.; Murakami, Y.; Okamoto, K.; Aizawa, T.; Torii, S. *Bull. Chem. Soc. Jpn.* **1988,** *61,* 310. doi:[10.1246/bcsj.61.310](http://dx.doi.org/10.1246/bcsj.61.310)
15. Wenkert, E.; Moeller, P. D. R.; Piettre, S. R. *J. Org. Chem.* **1987,** *52,* 3404. doi:[10.1021/jo00391a044](http://dx.doi.org/10.1021/jo00391a044)
16. Murphy, J. A.; Scott, K. A.; Sinclair, R. S.; Lewis, N. *Tetrahedron Lett.* **1997,** *38,* 7295. doi:[10.1016/S0040-4039(97)01695-X](http://dx.doi.org/10.1016/S0040-4039(97)01695-X)
17. Gassman, P. G.; van Bergen, T. J.; Gilbert, D. P.; Cue, B. W., Jr. *J. Am. Chem. Soc.* **1974,** *96,* 5495. doi:[10.1021/ja00824a028](http://dx.doi.org/10.1021/ja00824a028)
18. Ottoni, O.; Cruz, R.; Alves, R. *Tetrahedron* **1998,** *54,* 13915. doi:[10.1016/S0040-4020(98)00865-5](http://dx.doi.org/10.1016/S0040-4020(98)00865-5)
19. Liu, S.-F.; Wu, Q.; Schmider, H. L.; Aziz, H.; Hu, N.-X.; Popović, Z.; Wang, S. *J. Am. Chem. Soc.* **2000,** *122,* 3671. doi:[10.1021/ja9944249](http://dx.doi.org/10.1021/ja9944249)
20. Uchiyama, M.; Matsumoto, Y.; Nakamura, S.; Ohwada, T.; Kobayashi, N.; Yamashita, N.; Matsumiya, A.; Sakamoto, T. *J. Am. Chem. Soc.* **2004,** *126,* 8755. doi:[10.1021/ja039674a](http://dx.doi.org/10.1021/ja039674a)
